# *In vitro* antitumor activity, molecular dynamics simulation, DFT study, ADME prediction, and Eg5 binding of enastron analogues†

**DOI:** 10.1039/d3ra02904b

**Published:** 2023-06-28

**Authors:** Abdeslem Bouzina, Yousra Ouafa Bouone, Omar Sekiou, Mohamed Aissaoui, Tan-Sothea Ouk, Abdelhak Djemel, Rachida Mansouri, Malika Ibrahim-Ouali, Zihad Bouslama, Nour-Eddine Aouf

**Affiliations:** a Laboratory of Applied Organic Chemistry, Bioorganic Chemistry Group, Department of Chemistry, Sciences Faculty, Badji Mokhtar Annaba University Box 12 23000 Annaba Algeria abdeslem.bouzina@univ-annaba.dz bouzinaabdeslem@yahoo.fr; b Environmental Research Center (CRE) 23000 Annaba Algeria; c Laboratoire Peirene, EA7500 Université de Limoges 123 Avenue Albert Thomas 87000 Limoges Cedex France; d Research Unit in Medicinal Plants, URPM 3000 Laghouat Algeria; e Research Center of Biotechnology, CRBt 25000 Constantine Algeria; f Aix Marseille Univ, CNRS, Centrale Marseille, iSm2 Marseille F-13397 France

## Abstract

The objective of this study is to evaluate a series of molecules based on cyclosulfamide as potential anticancer agents. Additionally, the study aims to analyze the obtained results through *in silico* studies; by conducting experiments and utilizing theoretical methods. In this context, we investigated the cytotoxic activity of enastron analogues on three human cell lines PRI (lymphoblastic cell line) derived from B-cell lymphoma. JURKAT (ATCC TIB-152) acute T cell leukaemia and K562 (ATCC CLL-243) is a chronic myelogenous leukaemia. Most of the tested compounds showed good inhibitory activity compared with the reference ligand (chlorambucil). The 5a derivative demonstrated the strongest effect against all cancer cells used. Furthermore, molecular docking simulations of the Eg5–enastron analogue complex revealed that studied molecules have the ability to inhibit the Eg5 enzyme, as evidenced by their calculated docking score. Following the promising results from the molecular docking study, the complex Eg5–4a underwent a 100 ns molecular dynamics simulation using Desmond. During the simulation, the receptor-ligand pairing demonstrated substantial stability after the initial 70 ns. In addition, we used DFT calculations to analyze the electronic and geometric characteristics of the studied compounds. The HOMO and LUMO band gap energies, and the molecular electrostatic potential surface were also deducted for the stable structure of each compound. Also, we studied the prediction of absorption, distribution, metabolism and excretion (ADME) of the compounds.

## Abstract 2

The objective of this study is to evaluate a series of molecules based on cyclosulfamide as potential anticancer agents.

## Graphical abstract

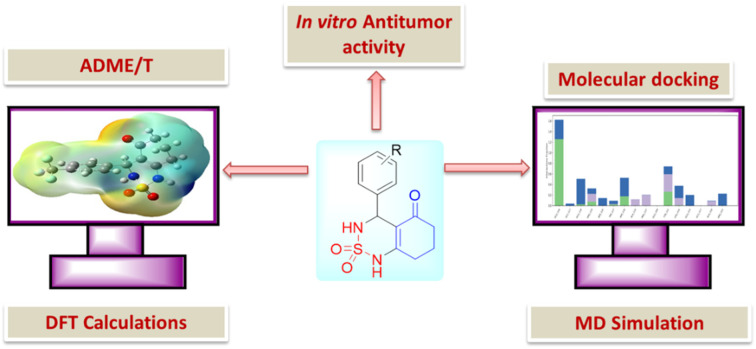

## Introduction

Cancerous diseases represent one of the biggest issues threatening global health, and are the second leading causes of mortality worldwide after cardiovascular diseases, accounting for 10 million deaths in 2020.^1^

Cancer is a disease occurring in different parts of the body by changes in cell functions caused by successive genes mutations that lead to abnormal cell growth, which generally results in the formation of a solid mass of cells called a tumor.^2,3^ In the past, the main ways to manage cancers were essentially radiotherapy and surgery.^2,4^ Then chemotherapy emerged as an important tool to face cancer cell proliferation especially after the effectiveness showed by nitrogen mustards in the treatment of malignant lymphoma in the 1940s.^5^ Afterward, a large number of chemotherapeutic agents were developed and proved their role against cancerous diseases with different mechanisms of actions citing alkylating agents, antimetabolites, antimicrotubular agents, DNA-interactive agents, molecular targeting agents, hormones, monoclonal antibodies and other biological agents.^6,7^ Despite the fact that there are several anticancer drugs effectively used in cancer treatment, the drug development in cancer chemotherapy is still an ongoing necessity in order to overcome the existing treatments harmful side effects.

**Fig. 1:**
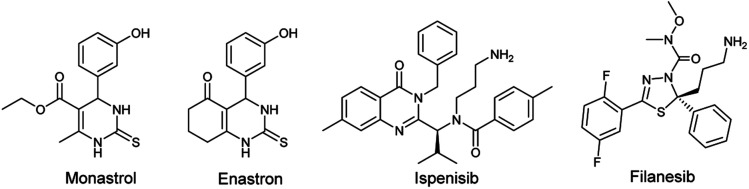
Chemical structure of some Eg5 inhibitors.

One of the human enzymes that are considered as interesting targets for drug development of cancer chemotherapy the kinesin spindle protein (KSP); a member of kinesin superfamily present in many tissues including testis, thymus, tonsils, and bone marrow.^8^ KSP also known as Eg5 plays a key role during the mitotic phase of cell division by forming bipolar spindles.^9^ An overexpression of this enzyme is observed in solid tumors and leukemia. Eg5 inhibition can result in stopping mitosis and causing apoptosis of cancer cell lines.^10^ The first small molecule showing inhibitory activity against Eg5 was the pyrimidine-based compound known as monastrol. Since its identification by Mayer *et al.* in 1999, Monastrol was considered as the prototype of KSP/Eg5 inhibitors and the research for new molecules targeting the mitotic kinesin Eg5 has emerged.^11^ Subsequently, a great number of heterocyclic-based Eg5 inhibitors were reported as anticancer and antitumor agents including other DHPMs such as enastron,^12^ quinazolines such as ispinesib,^13^ thiadiazoles such as filanesib,^14^ thiazolopyrimidines, isoquinolines, and many others (Fig. 1).^15^

On the other hand, benzothiadiazinone dioxides (also called cyclosulfamides) are important heterocyclic-based compounds bearing the sulfamide moiety that is recognized as a remarkable pharmacophore with a high number of applications in the fields of medicinal chemistry and drug development.^16^ Different pharmacological activities of cyclic sulfamides were reported in the literature comprising antibacterial,^17^ antiviral,^18^ anti-inflammatory,^19^ and anticancer activities.^20^ Further, the high capacity of cyclosulfamides in inhibiting many enzymes and target proteins such as phosphodiesterase 7 (PDE7),^21^ aspartic proteases,^22^ and serine proteases^23^ turned interest of scientists to conceive more molecules containing this scaffold.

In this context, our study focused on the evaluation of a series of cyclosulfamide-based derivatives exclusively as antitumor agents with the aim of developing new candidate molecules against cancer disease. To analyze the obtained results from the biological activities, *in silico* investigations against Eg5 enzyme were also performed to verify the antitumor activities. In addition, we studied the chemical reactivity of studied compounds using DFT. Furthermore, ADME analyses have been executed to evaluate the performance of physico-chemical properties of tested compounds.

## Materials and methods

### Chemistry

#### Chemical methods

All chemicals and solvents were purchased from common commercial sources and were used as received without any further purification. All reactions were monitored by TLC on silica Merck 60 F_254_ percolated aluminum plates and were developed by spraying with ninhydrin solution. Proton nuclear magnetic resonance (^1^H NMR) spectra were recorded on a Brücker spectrometer at 400 MHz. Chemical shifts are reported in *δ* units (ppm) with TMS as reference (*δ* 0.00). All coupling constants (*J*) are reported in hertz. Multiplicity is indicated by one or more of the following: s (singlet), brs (broad singlet), d (doublet), dd (doublet of doublets), t (triplet), td (triplet of doublets), q (quartet), m (multiplet). Carbon nuclear magnetic resonance (^13^C NMR) spectra were recorded on a Brücker at 100 MHz. Chemical shifts are reported in *δ* units (ppm) relative to DMSO-*d*_6_ (*δ* 39.52). Infrared spectra were recorded on a PerkinElmer 600 spectrometer. The purity of the final compounds (greater than 95%) was determined by uHPLC/MS on an Agilent 1290 system using a Agilent 1290 Infinity ZORBAX Eclipse Plus C18 column (2.1 mm × 50 mm, 1.8 μm particle size) with a gradient mobile phase of H_2_O/CH_3_CN (90 : 10, v/v) with 0.1% of formic acid to H_2_O/CH_3_CN (10 : 90, v/v) with 0.1% of formic acid at a flow rate of 0.5 mL min^−1^, with UV monitoring at the wavelength of 254 nm with a run time of 10 min. Microanalysis spectra were performed by Elementar Analyzer (Euro E.A. 3000-V3.0-single-2007) and the determined values were within the acceptable limits of the calculated values. Melting points were recorded on a Büchi B-545 apparatus in open capillary tubes. Microwave assisted reactions were carried out using a Biotage Initiator Microwave Synthesizer 2.0 with a nominal power of 400 W. The reactions were carried out in a reactor to MW (volume: 10 mL) under pression at room temperature.

#### General procedure for the synthesis of benzothiadiazinone dioxide

The following method for the synthesis of studied compounds in this work is previously described by our group.^24^

In a reactor MW (volume: 10 mL) taken a mixture of aldehyde (1 mmol), sulfamide (1 mmol), and cyclohexane-1,3-dione (1 mmol) in the presence of H_2_SO_4_ (0.1 mmol)/CH_3_COOH (0.9 mmol) as catalyst under solvent-free at room temperature. The reaction mixture was subjected to microwave irradiation for appropriate time. After completion of the reaction (5–10 min), as indicated by TLC, silica gel; dichloromethane : methanol (9 : 1), mixture of ethanol and acetone (9 : 1) or mixture of ethyl acetate and *n*-hexane (5/5) was added to the reaction and pure product was crystallized to 6 °C overnight. The products were finally filtered and dried.

##### 4-Phenyl-4,6,7,8-tetrahydro-1*H*-benzo[*c*][1,2,6]thiadiazin-5(3*H*)-one 2,2-dioxide (1a, C_13_H_14_N_2_O_3_S)

Cristal; 92% yield; *R*_f_ = 0.20 (CH_2_Cl_2_/CH_3_OH:85/15); ^1^H NMR (400 MHz, DMSO-*d*_6_): *δ* = 1.95–2.02 (m, 2H, CH_2_), 2.20–2.29 (m, 1H, CH_2_), 2.38–2.43 (m, 1H, CH_2_), 2.43–2.50 (m, 2H, CH_2_), 5.35 (d, *J* = 6.0 Hz, 1H, CH*), 7.20–7.24 (m, 5H, H–Ar), 7.93 (d, *J* = 4.4 Hz, 1H, NH–CH*), 10.84 (brs, 1H, NH) ppm; ^13^C NMR (100 MHz, DMSO-*d*_6_): *δ* = 22.98, 27.55, 36.99, 55.69, 107.90, 126.78, 127.45, 127.81, 139.36, 156.00, 194.42 ppm; IR (KBr): *ν* = 3286 (NH), 3121 (NH), 1632 (C=O), 1598 (C=C), 1356 and 1171 (SO_2_) cm^−1^; MS: (*m*/*z*) = 279.0 (M + 1); anal. calc. for C_13_H_14_N_2_O_3_S: C, 56.10; H, 5.07; N, 10.07; found: C, 56.13; H, 5.02; N, 10.03.

##### 4-(2-Fluorophenyl)-4,6,7,8-tetrahydro-1*H*-benzo[*c*][1,2,6]thiadiazin-5(3*H*)-one 2,2-dioxide (2a, C_13_H_13_FN_2_O_3_S)

White powder; 91% yield; *R*_f_ = 0.19 (CH_2_Cl_2_/CH_3_OH:85/15); ^1^H NMR (400 MHz, DMSO-*d*_6_): *δ* = 1.93–2.05 (m, 2H, CH_2_), 2.21–2.29 (m, 1H, CH_2_), 2.30–2.40 (m, 1H, CH_2_), 2.52–2.65 (m, 2H, CH_2_), 5.60 (d, *J* = 8.0 Hz, 1H, CH*), 7.02 (t, *J* = 8.0 Hz, 1H, H–Ar_*ortho*_), 7.13 (t, *J* = 12.0 Hz, 2H, H–Ar_*para*_,_*meta*_), 7.27 (q, *J* = 8.1 Hz, 1H, H–Ar_*meta*_), 8.01 (d, *J* = 8.0 Hz, 1H, NH–CH*), 10.91 (brs, 1H, NH) ppm; ^13^C NMR (100 MHz, DMSO-*d*_6_): *δ* = 20.75, 27.83, 36.30, 49.75, 106.45, 115.13, 113.11, 126.31, 129.04, 129.65, 158.78, 164.73, 195.92 ppm; IR (KBr): *ν* = 3261 (NH), 3108 (NH), 1606 (C=O), 1488 (C=C), 1353 and 1171 (SO_2_) cm^−1^; MS: (*m*/*z*) = 297.1 (M + 1); anal. calc. for C_13_H_13_FN_2_O_3_S: C, 52.69; H, 4.42; N, 9.45; found: C, 52.60; H, 4.37; N, 9.49.

##### 4-(3-Fluorophenyl)-4,6,7,8-tetrahydro-1*H*-benzo[*c*][1,2,6]thiadiazin-5(3*H*)-one 2,2-dioxide (3a, C_13_H_13_FN_2_O_3_S)

White powder; 92% yield; *R*_f_ = 0.18 (CH_2_Cl_2_/CH_3_OH:85/15); ^1^H NMR (400 MHz, DMSO-*d*_6_): *δ* = 1.92–2.03 (m, 2H, CH_2_), 2.21–2.27 (m, 1H, CH_2_), 2.29–2.38 (m, 1H, CH_2_), 2.50–2.57 (m, 2H, CH_2_), 5.59 (d, *J* = 7.01 Hz, 1H, CH*), 7.02 (td, *J*_1_ = 7.60, *J*_2_ = 1.0 Hz, 1H, H–Ar_*meta*_), 7.02–7.22 (m, 2H, H–Ar_*ortho*,*para*_), 7.28 (ddd, *J*_1_ = 15.2, *J*_2_ = 5.4, *J*_3_ = 1.8 Hz, 1H, H–Ar_*meta*_), 8.05 (d, *J* = 7.20 Hz, 1H, NH–CH*), 10.98 (brs, 1H, NH) ppm; ^13^C NMR (100 MHz, DMSO-*d*_6_): *δ* = 20.99, 28.05, 38.60, 49.96, 106.66, 114.93, 123.33, 126.53, 129.29, 129.99, 158.94, 161.46, 196.19 ppm; IR (KBr): *ν* = 3429 (NH), 3268 (NH), 1632 (C=O), 1606 (C=C), 1340 and 1168 (SO_2_) cm^−1^; MS: (*m*/*z*) = 297.0 (M + 1); anal. calc. for C_13_H_13_FN_2_O_3_S: C, 52.69; H, 4.42; N, 9.45; found: C, 52.64; H, 4.48; N, 9.52.

##### 4-(4-Hydroxyphenyl)-4,6,7,8-tetrahydro-1*H*-benzo[*c*][1,2,6]thiadiazin-5(3*H*)-one 2,2-dioxide (4a, C_13_H_14_N_2_O_4_S)

White powder; 90% yield; *R*_f_ = 0.20 (CH_2_Cl_2_/CH_3_OH:85/15); ^1^H NMR (400 MHz, DMSO-*d*_6_): *δ* = 1.92–2.05 (m, 2H, CH_2_), 2.24–2.26 (m, 1H, CH_2_), 2.28–2.30 (m, 1H, CH_2_), 2.40–2.54 (m, 2H, CH_2_), 5.42 (d, *J* = 6.8 Hz, 1H, CH*), 6.38 (s, 1H, OH), 7.40 (d, *J* = 8.4 Hz, 2H, H–Ar), 7.60 (d, *J* = 8.0 Hz, 2H, H–Ar), 8.11 (d, *J* = 6.8 Hz, 1H, NH–CH*), 11.01 (brs, 1H, NH) ppm; ^13^C NMR (100 MHz, DMSO-*d*_6_): *δ* = 20.01, 28.07, 36.49, 55.17, 107.03, 124.40, 124.44, 125.72, 127.31, 128.69, 144.33, 193.69 ppm; IR (KBr): *ν* = 3475 (OH), 3261 (NH), 1702 (C=O), 1639 (C=C), 1356 and 1171 (SO_2_) cm^−1^; MS: (*m*/*z*) = 311.1 (M + H_2_O); anal. calc. for C_13_H_14_N_2_O_4_S: C, 52.05; H, 4.79; N, 9.52; found: C, 52.12; H, 4.84; N, 9.49.

##### 4-(4-(Trifluoromethyl)phenyl)-4,6,7,8-tetrahydro-1*H*-benzo[*c*][1,2,6]thiadiazin-5(3*H*)-one 2,2-dioxide (5a, C_14_H_13_F_3_N_2_O_3_S)

White powder; 88% yield; *R*_f_ = 0.21 (CH_2_Cl_2_/CH_3_OH:85/15); ^1^H NMR (400 MHz, DMSO-*d*_6_): *δ* = 1.68–1.82 (m, 2H, CH_2_), 1.83–1.86 (m, 1H, CH_2_), 1.89–2.02 (m, 1H, CH_2_), 2.15–2.48 (m, 2H, CH_2_), 5.07 (s, 1H, CH*), 6.75–7.08 (m, 3H, H–Ar), 7.10–7.13 (m, 1H, H–Ar), 7.14–7.21 (m, 1H, NH–CH*), 10.47 (brs, 1H, NH) ppm; ^13^C NMR (100 MHz, DMSO-*d*_6_): *δ* = 20.40, 27.26, 36.67, 48.11, 101.16, 111.58, 115.52, 121.38, 124.24, 129.27, 150.12, 166.99, 196.00 ppm; IR (KBr): *ν* = 3225 (NH), 3116 (NH), 1702 (C=O), 1598 (C=C), 1354 and 1170 (SO_2_) cm^−1^; MS: (*m*/*z*) = 324.0 (M + 1); anal. calc. for C_13_H_13_N_3_O_5_S: C, 48.55; H, 3.78; N, 8.09; found: C, 48.62; H, 3.84; N, 8.13.

##### 4-(2-Hydroxy-4-nitrophenyl)-4,6,7,8-tetrahydro-1*H*-benzo[*c*][1,2,6]thiadiazin-5(3*H*)-one 2,2-dioxide (6a, C_13_H_13_N_3_O_6_S)

Colorless powder: insoluble. 88% yield; *R*_f_ = 0.29 (CH_2_Cl_2_/CH_3_OH:85/15), IR (KBr): *ν* = 3435 (NH), 3267 (NH), 1640 (C=O), 1606 (C=C), 1341 and 1172 (SO_2_) cm^−1^; anal. calc. for C_13_H_13_N_3_O_6_S: C, 46.02; H, 3.86; N, 12.38; found: C, 46.09; H, 3.89; N, 12.33.

##### 4-(4-Bromophenyl)-4,6,7,8-tetrahydro-1*H*-benzo[*c*][1,2,6]thiadiazin-5(3*H*)-one 2,2-dioxide (7a, C_13_H_13_BrN_2_O_3_S)

Yellow powder; 90% yield; *R*_f_ = 0.20 (CH_2_Cl_2_/CH_3_OH:85/15); ^1^H NMR (400 MHz, DMSO-*d*_6_): *δ* = 1.92–2.15 (m, 2H, CH_2_), 2.27–2.30 (m, 1H, CH_2_), 2.37–2.43 (m, 1H, CH_2_), 2.50–2.57 (m, 2H, CH_2_), 5.32 (d, *J* = 6.4 Hz, 1H, CH*), 7.14 (d, *J* = 8.4 Hz, 2H, H–Ar), 7.42 (d, *J* = 7.2 Hz, 2H, H–Ar), 8.02 (d, *J* = 6.8 Hz, 1H, NH–CH*), 10.97 (brs, 1H, NH) ppm; ^13^C NMR (100 MHz, DMSO-*d*_6_): *δ* = 21.16, 27.06, 36.28, 55.08, 107.32, 119.97, 130.14, 130.39, 139.03, 156.60, 193.73 ppm; IR (KBr): *ν* = 3253 (NH), 3128 (NH), 1703 (C=O), 1602 (C=C), 1354 and 1170 (SO_2_) cm^−1^; MS: (*m*/*z*) = 357.0 (M + 1), 359.0 (M + 2); anal. calc. for C_13_H_13_BrN_2_O_3_S: C, 43.71; H, 3.67; N, 7.84; found: C, 43.67; H, 3.70; N, 7.86.

##### 4-(4-Chlorophenyl)-4,6,7,8-tetrahydro-1*H*-benzo[*c*][1,2,6]thiadiazin-5(3*H*)-one 2,2-dioxide (8a, C_13_H_13_ClN_2_O_3_S)

White powder; 92% yield; *R*_f_ = 0.20 (CH_2_Cl_2_/CH_3_OH:85/15); ^1^H NMR (400 MHz, DMSO-*d*_6_): *δ* = 1.92–2.03 (m, 2H, CH_2_), 2.22–2.24 (m, 1H, CH_2_), 2.25–2.30 (m, 1H, CH_2_), 2.37–2.57 (m, 2H, CH_2_), 5.31 (d, *J* = 6.8 Hz, 1H, CH*), 7.14 (d, *J* = 8.4 Hz, 2H, H–Ar_*ortho*_), 7.42 (d, *J* = 8.4 Hz, 2H, H–Ar_*meta*_), 8.02 (d, *J* = 6.8 Hz, 1H, NH–CH*), 10.97 (brs, 1H, NH) ppm; ^13^C NMR (100 MHz, DMSO-*d*_6_): *δ* = 20.99, 28.04, 36.54, 55.08, 107.32, 119.57, 130.14, 130.39, 139.02, 156.35, 193.72 ppm; IR (KBr): *ν* = 3431 (NH), 3254 (NH), 1701 (C=O), 1601 (C=C), 1352 and 1169 (SO_2_) cm^−1^; MS: (*m*/*z*) = 313.0 (M + 1); anal. calc. for C_13_H_13_ClN_2_O_3_S: C, 49.92; H, 4.19; N, 8.96; found: C, 49.84; H, 4.23; N, 8.88.

##### 4-(2-Chlorophenyl)-4,6,7,8-tetrahydro-1*H*-benzo[*c*][1,2,6]thiadiazin-5(3*H*)-one 2,2-dioxide (9a, C_13_H_13_ClN_2_O_3_S)

White powder; 91% yield; *R*_f_ = 0.19 (CH_2_Cl_2_/CH_3_OH:85/15); ^1^H NMR (400 MHz, DMSO-*d*_6_): *δ* = 1.91–2.07 (m, 2H, CH_2_), 2.27–2.32 (m, 1H, CH_2_), 2.34–2.40 (m, 1H, CH_2_), 2.53–2.64 (m, 2H, CH_2_), 5.58 (d, *J* = 6.4 Hz, 1H, CH*), 7.02 (td, *J*_1_ = 7.5, *J*_2_ = 1.1 Hz, 2H, H–Ar), 7.11 (ddd, *J*_1_ = 16.4, *J*_2_ = 8.5, *J*_3_ = 1.3 Hz, 1H, H–Ar), 7.27 (ddd, *J*_1_ = 15.3, *J*_2_ = 5.4, *J*_3_ = 1.8 Hz, 1H, H–Ar), 8.02 (d, *J* = 6.8 Hz, 1H, NH–CH*), 10.97 (brs, 1H, NH) ppm; ^13^C NMR (100 MHz, DMSO-*d*_6_): *δ* = 20.98, 28.04, 36.53, 49.89, 106.52, 114.68, 114.90, 123.30, 126.38, 126.51, 129.25, 129.88, 158.98, 193.85 ppm; IR (KBr): *ν* = 3272 (NH), 3126 (NH), 1702 (C=O), 1606 (C=C), 1353 and 1171 (SO_2_) cm^−1^; MS: (*m*/*z*) = 313.0 (M + 1); anal. calc. for C_13_H_13_ClN_2_O_3_S: C, 49.92; H, 4.19; N, 8.96; found: C, 49.97; H, 4.25; N, 8.89.

### Antitumor activity

#### HeLa cell culture

The continuous human cell lines HeLa (epithelial cervical cancer cell line) (ATCC, Manassas, VA, USA) were used to investigate the cytotoxicity effect of new products. This adherent cell line was grown in RPMI 1640 medium (Gibco, Grand Island, NY, USA) supplemented with 10% (v/v) foetal calf serum (FCS) (Gibco) and 2 mM l-glutamine (Sigma-Aldrich) in tissue culture flasks (Nunc, Roskilde, Denmark). It was sub-cultured twice a week and kept at 37 °C in a humidified and controlled atmosphere of 95% air and 5% CO_2_.

#### MTT cell proliferation assay

The MTT [3-(4,5-dimethylthiazolyl-2)-2,5-diphenyltetrazolium-bromide] (Sigma-Aldrich) cell proliferation assay measures the cell proliferation rate and conversely, the reduction in cell viability when metabolic events lead to apoptosis or necrosis. The yellow compound MTT (Sigma) is reduced by mitochondrial dehydrogenases to the water insoluble blue formazan compound, depending on the viability of the cells.^25^

Cells (3 × 10^4^ cells per mL) were grown on microtiter plates (200 mL of cell suspension per well) in 96 well microplates with serial dilutions of the compounds. 48 h later, 10 mL of a MTT solution (5 mg mL^−1^ in PBS) were added in each well. The plate was incubated for 4 h at 37 °C in a CO_2_ incubator. Then, 180 mL of medium were removed from each well and 180 mL of DMSO were added to each sample. When all the crystals were dissolved, absorbance was measured at 570 nm with a microplate reader (Elx 800 microplate reader).

### Computational methods

#### Molecular docking

The X-ray crystal structure of Eg5–enastron complex (PDB ID: 2X7C) was obtained from the Protein Data Bank,^26^ and was prepared with protein preparation wizard in Schrodinger suites. The three-dimensional structures of the derivatives were constructed using Maestro software, and prepared with Ligprep using OPLS3e force field.^27^

The final prepared PDB file of the protein and synthesized analogues were submitted in order to run docking process. Docking studies were performed by Glide software^28^ at extra precision.^29^ Output files of docked analogues along with Eg5 protein were visualised on Chimera software.^30^

#### Molecular dynamics simulation

Molecular dynamics simulations (MD) were performed using Desmond.^31^ The best docking complex for the identified inhibitors from the biological experiments were taken as the initial coordinates for MD simulations. A 10 Å cubic water box employing the TIP3P water model was used for the solvation of the system with OPLS 2005 force field. Sodium ions were added as counter ions to neutralize the systems. We have then subjected the system to minimization using an energy gradient convergence threshold of 1 kcal mol^−1^ Å^−1^ and pre-equilibration using the default six-step relaxation protocol implemented in Desmond. The first two steps are minimization steps, one in which the solute is kept restrained and another without restraints. Further, steps three to six are short MD simulations of 12 ps, 12 ps, and 24 ps each using the NPT ensemble at 10, 10, 300, and 300 K, respectively. Subsequently, a 100 nanosecond (ns) MD simulation production run was performed. Remaining parameters were kept at the Desmond default values. We have visualized the protein–ligand complexes and analyzed the MD trajectory using Maestro. The detailed analyses were performed using the Simulation Event Analysis tool of Desmond.

#### Density functional theory (DFT) analysis

Molecular geometry the gas phase structure optimization of benzothiadiazinone derivatives (1a–9a) is optimized using DFT at B3LYP method,^32^ with the basis set of 6-31G(d,p) implemented by Gaussian 09 package.^33^ Frontier molecular orbitals and global reactivity descriptors the highest occupied molecular orbital (HOMO) and lowest un-occupied molecular orbital (LUMO),^34^ energy gap and chemical reactivity descriptors are calculated at DFT/B3LYP/6-31G(d,p) method.

#### Pharmacokinetics analysis (ADME)

Various *in silico* methods aim to predict ADME parameters based on the molecular structure of compounds. One significant contribution in this field was made by Lipinski *et al.* who studied orally active compounds to establish physicochemical ranges that increase the probability of a compound being an oral drug. This approach, known as the rule-of-five, established a correlation between pharmacokinetic and physicochemical parameters.^35^

Regarding this part of the work, we used the SwissADME web tool *via* the link: https://www.swissadme.ch, which provides free access to a pool of quick yet reliable predictive models for physicochemical properties, pharmacokinetics, drug-likeness, and medicinal chemistry friendliness, including in-house effective techniques like the BOILED-Egg, iLOGP, and Bioavailability Radar.^36^

The Drug Likeness Score (DLS) results were determined using the Molsoft web tool *via* the link: https://www.molsoft.com.^37^

## Results and discussion

### Chemistry

The used compounds in this study were described by our team previously.^24^

A single step of the Biginelli cyclo-condensation reaction was used to create the investigated benzothiadiazinone dioxides.

**Scheme 1:**
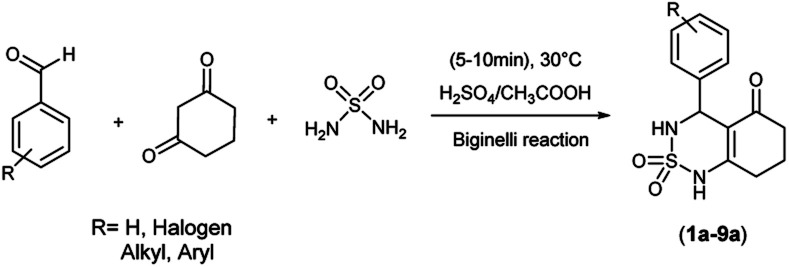
Multicomponent reaction synthesis of benzothiadiazinone dioxides.

The reaction involved combining different aromatic aldehydes, cyclohexanedione, and sulfamide with a catalytic amount of H_2_SO_4_/CH_3_COOH (1/9) under microwave irradiation and solvent-free conditions at room temperature to produce the desired derivatives (Scheme 1).

Table 1 displays the structures of synthesized benzothiadiazinone dioxides (1a–9a).

Structures of synthesized derivatives of cyclosulfamide

**Table:** 

Code	1a	2a	3a	4a	5a
Structure	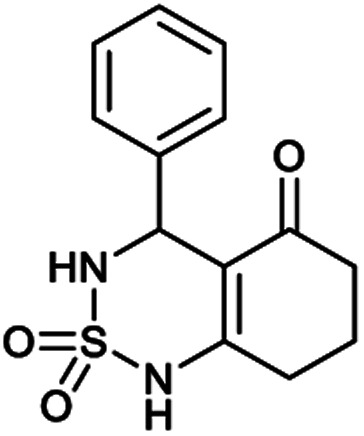	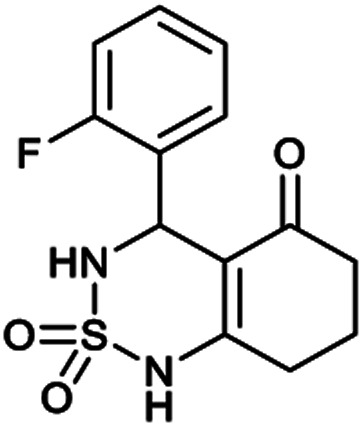	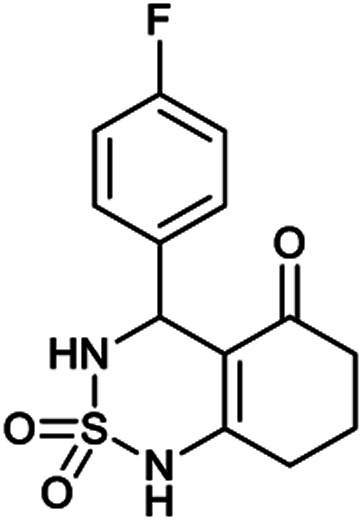	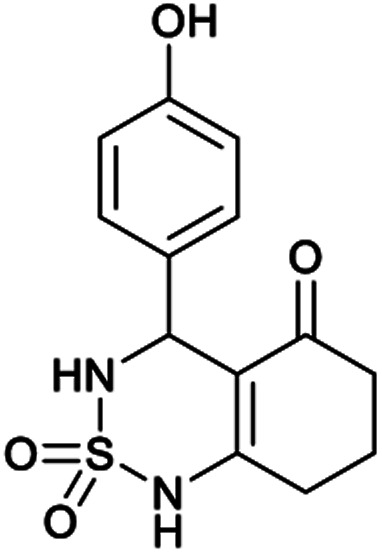	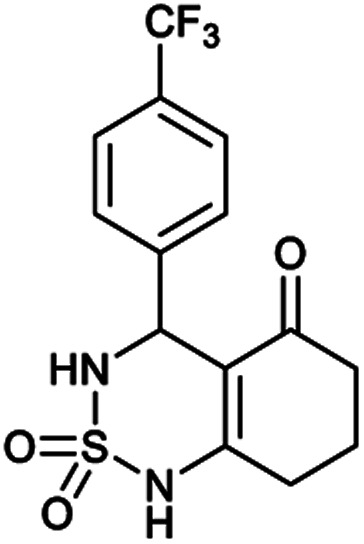

**Table:** 

Code	6a	7a	8a	9a
Structure	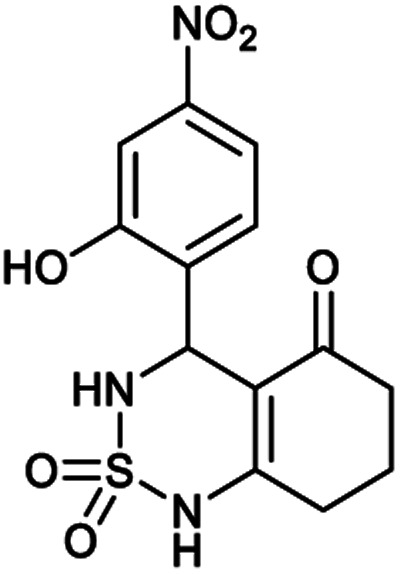	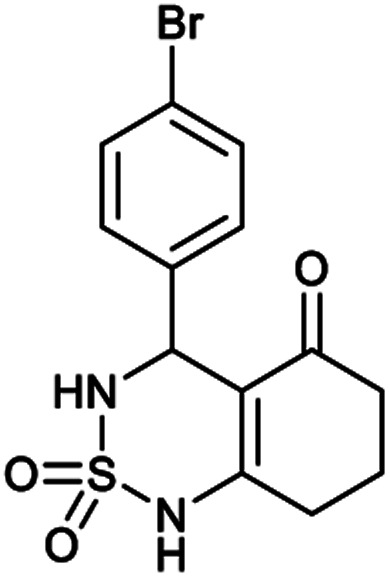	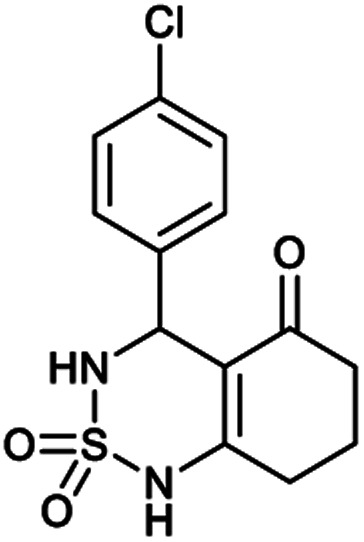	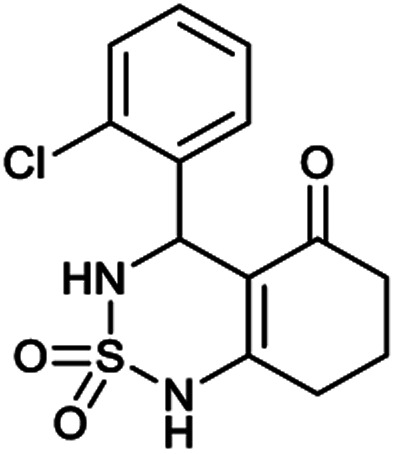

### Mechanistic proposal

**Scheme 2:**
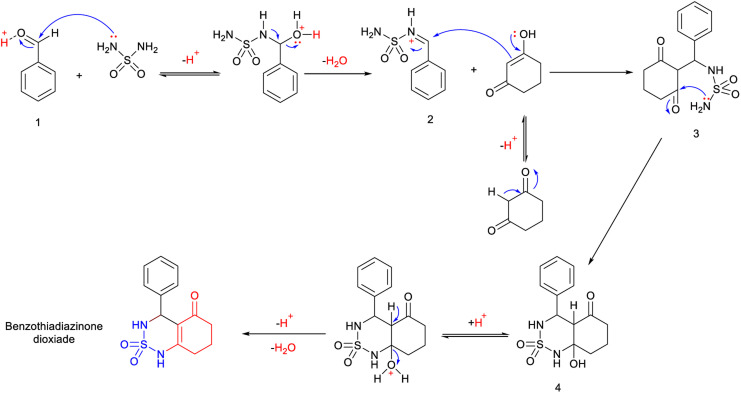
Mechanistic proposal for the synthesis of benzothiadiazinone dioxide.

A possible mechanism for this reaction is presented in Scheme 2. The reaction begins with the aldehyde being protonated and the formation of benzylideneoxonium cation 1, facilitated by the exchange of its proton with H_2_SO_4_/CH_3_COOH. Furthermore, microwave irradiation assists in accelerating this process and renders the carbonyl group more susceptible to nucleophilic attack by the sulfamide. This step is succeeded by the formation of activated *N*-iminium ion intermediate 2. Subsequently, the latter undergoes nucleophilic attack by the tautomer of cyclohexane-1,3-dione on the double bond of *N*-iminium ion 2, resulting in the formation of open-chain intermediate 3. Eventually, intermediate 3 cyclizes to yield hydrobenzothiadiazinone dioxide 4. The final benzothiadiazinone dioxide product is obtained through acid-catalyzed and microwave irradiation-induced elimination of water.

### Antitumor results

Antimitotic drugs have the potential to be an interesting target for medicinal chemists due to their possible therapeutic use in cancer chemotherapy. These drugs prevent the proliferation of cancer cells by interfering with the polymerization or depolymerization of microtubules.^38^ Several families of compounds have been described in the literature showing potent cytotoxic activity against human cancer cell lines which include diarylisoxazole,^39^ sulfonamide^40^ and benzothiadiazine.^41^

In this context and to develop new active antimitotic agents, we evaluated a series of benzothiadiazinone dioxide against human cancer cell lines, including LCL B cells (PRI), chronic myelogenous leukaemia cells (K562) and T-lymphoma cells (JURKAT) using MTT assay.^42^ Chlorambucil (CLB) was used as a reference.

The biological activity of studied derivatives was determined as IC_50_ values (μM) and was calculated by logistic regression analysis of dose response curves plotting between the percentage of viability and the concentration of the tested compounds.

**Fig. 2:**
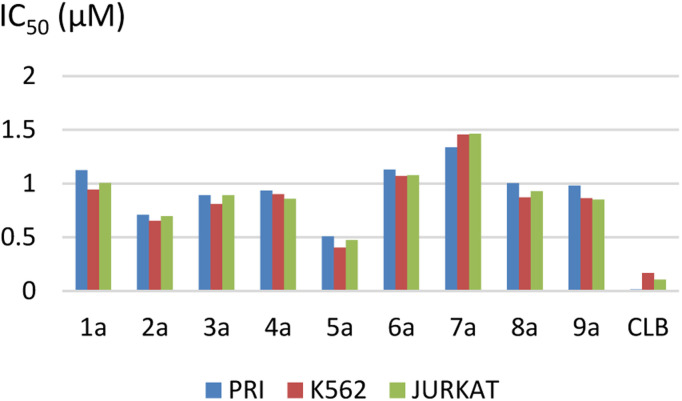
Representation of IC_50_ values.

The results are summarized in Table 2 and Fig. 2.

**Table:** Antitumor activity of compounds 1a–9a and standard drug CLB

Code	IC_50_ (μM) ± SD		
	PRI	K562	JURKAT
1a	1.123 ± 0.157	0.943 ± 0.095	1.004 ± 0.264
2a	0.708 ± 0.054	0.652 ± 0.075	0.697 ± 0.121
3a	0.891 ± 0.108	0.810 ± 0.115	0.891 ± 0.108
4a	0.934 ± 0.150	0.901 ± 0.131	0.858 ± 0.097
5a	0.510 ± 0.064	0.404 ± 0.045	0.474 ± 0.089
6a	1.131 ± 0.198	1.071 ± 0.121	1.079 ± 0.099
7a	1.337 ± 0.210	1.456 ± 0.307	1.464 ± 0.271
8a	1.003 ± 0.207	0.871 ± 0.173	0.930 ± 0.133
9a	0.982 ± 0.133	0.864 ± 0.274	0.851 ± 0.133
CLB	0.015 ± 0.001	0.167 ± 0.073	0.106 ± 0.065

The IC_50_ ranged from 0.51 ± 0.06 to 1.33 ± 0.21 μM for the PRI cell, from 0.40 ± 0.04 to 1.45 ± 0.30 μM for the K562 cell, and from 0.47 ± 0.08 to 1.46 ± 0.27 μM for the JURKAT cell.

We observed that the activity of compounds against the three human cancer cell lines was somewhat influenced by the structural diversity of the radicals on the aromatic ring.

The basic compound 1a showed moderate activity (IC_50_ = 1.12 ± 0.15 μM) compared with the reference drug CLB (IC_50_ = 0.015 ± 0.001 μM). In addition, the compounds bearing a fluorine atom on the aromatic ring in different positions (2a, 3a, and 5a) showed the best activities against PRI and K562 cancer cells. Further, best JURKAT cells growth inhibition was found in compounds 2a, 5a, and 9a.

The obtained results of the biological activity on the three cells used is most effectively demonstrated by derivative 5a (PRI: IC_50_ = 0.51 ± 0.06 μM, K562: IC_50_ = 0.40 ± 0.04 μM, and JURKAT: IC_50_ = 0.47 ± 0.08 μM), which can be attributed to the presence of a trifluoromethyl group substituting three fluorine atoms in the *para* position of the aromatic ring.

Among the tested compounds, both compound 4a with a hydroxyl group substitution in the *para* position of the aromatic ring and compound 9a with a chlorine group substitution in the *ortho* position exhibited satisfactory cytotoxicity, demonstrating an IC_50_ below 1 μM against all three tested cells.

Derivatives substituted by a bulky group in the *para* position 6a, 7a, and 8a are the least active, especially the derivative 7a (PRI: IC_50_ = 1.33 ± 0.21 μM, K562: IC_50_ = 1.45 ± 0.307 μM, and JURKAT: IC_50_ = 1.46 ± 0.27 μM) which is substituted by a bromine atom.

### *In silico* study

#### Molecular docking study

**Fig. 3:**
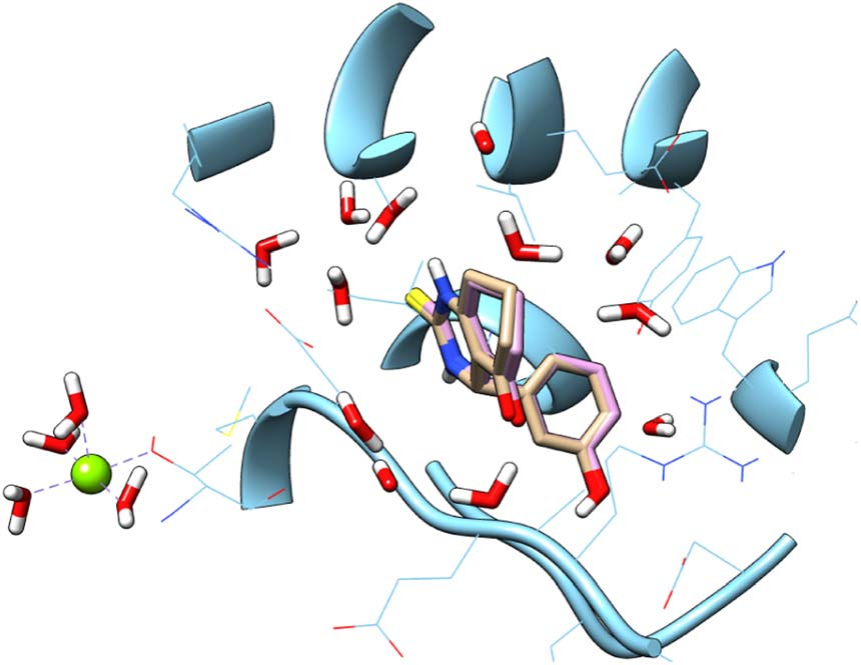
Docked and co-crystalized enastron in Eg5 enzyme after self-docking calculation.

Human kinesin Eg5, which plays an essential role in mitosis by establishing the bipolar spindle, has proven to be an interesting drug target for the development of cancer chemotherapeutics.^43^ A common approach in cancer chemotherapy is development of drugs that interrupt the mitosis phase of cell division. Enastron is a known kinesin inhibitor.^26^ In this study, a number of enastron analogues have been synthesized, in which (C=S) group has been replaced with sulfonyl group (SO_2_), moreover other substitution on the phenyl ring. In the aim to achieve the interaction on the active site, a docking study was carried out. Accuracy of docking protocol was examined by re-docking of enastron in the active site of Eg5 enzyme (self-docking). Fig. 3 shows docked enastron and co-crystallized one in almost same position among the receptor (RMSD = 0.22 Å) that confirmed validation of docking protocol using extra precision glide (XP) scoring function, in presence of water molecules that are not beyond 5 Å from reference ligand.

**Fig. 4:**
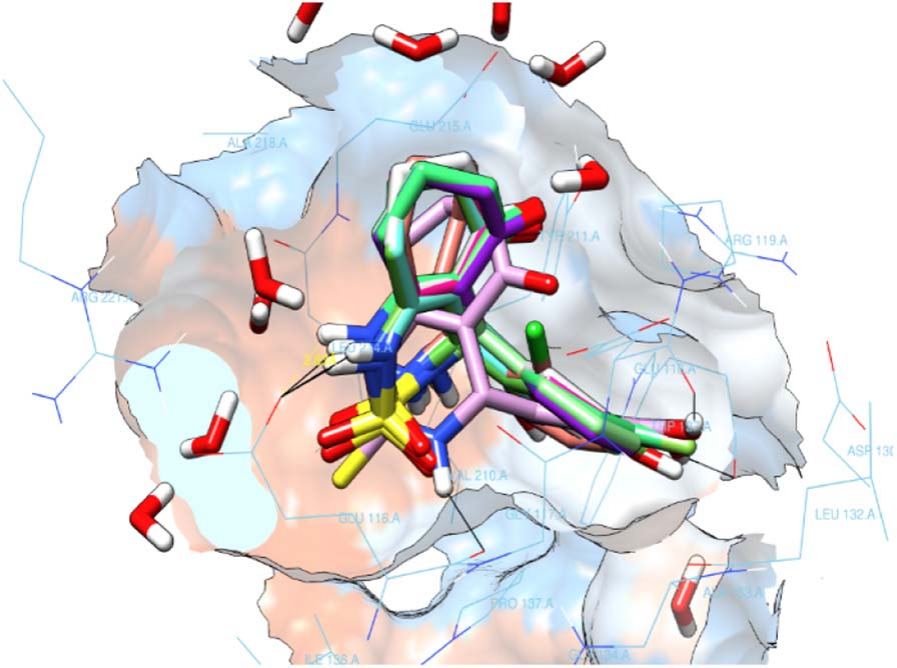
Superimposition of the docked enastron derivatives in the active site.

All enastron derivatives were docked into the active site of Eg5, and most studied compounds proved interesting stability inside the cavity (Fig. 4) with a glide score close to the one corresponding to the co-crystallized ligand, as shown in Table 3. Therefore, we were interested in studying the interaction of compounds that maintain hydrogen bonds, as well as hydrophobic interactions with residues of the active site.

**Table:** Ranking of enastron derivatives after docking study

Ligands	Glide score
4a	−6.95
9a	−6.64
1a	−6.58
3a	−6.58
2a	−6.56
5a	−6.54
6a	−6.30
8a	−5.30
7a	−5.27
Enastron	−9.448

**Fig. 5:**
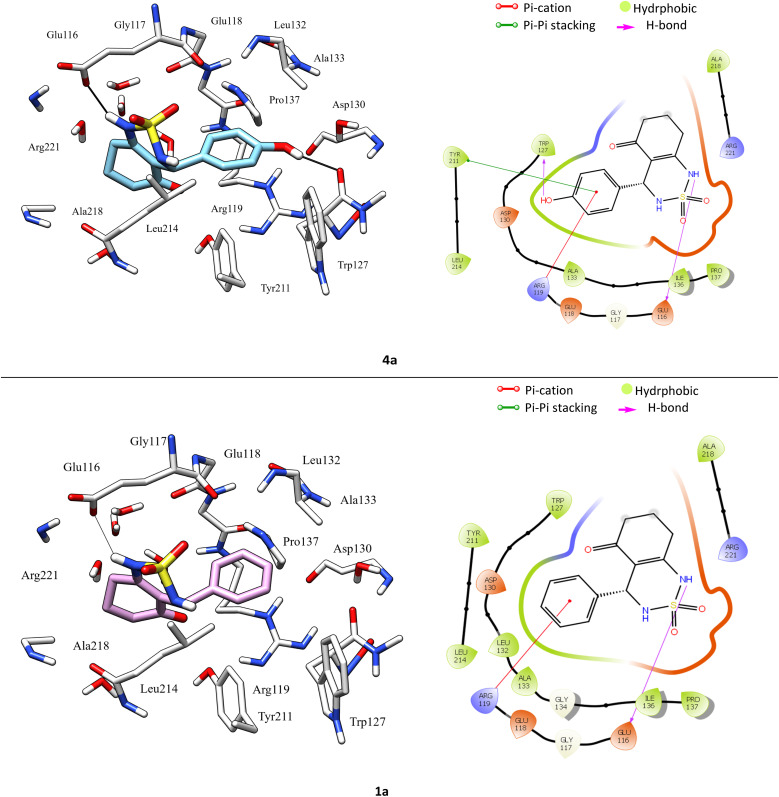
3D left and 2D right binding disposition of compounds 1a and 4a after docking calculations in the active site of Eg5 enzyme. The amino acid residues were shown as stick model and H-bonds were shown as black lines.

According to protein ligand interaction after visual check, the most consistent interaction is H-bond donor between side chain of Glu116 and nitrogen atom on cyclosulfamide ring. Synthesized ligands have shown mentioned interactions such as the co-crystallized ligand (figure ESI data†), and a backbone H-bond donor interaction was also detected between Trp127 and oxygen atom in the phenyl ring of compound 4a (Fig. 5). Due to this last interaction, the phenyl ring is inserted into a lipophilic cavity mainly delimited by Trp127, Ala133, Leu132, Tyr211 in which this group made Pi–Pi stacking interaction with the latter, which explains its great value of glide score. Therefore, we were interested in studying the interaction of these compounds.

**Fig. 6:**
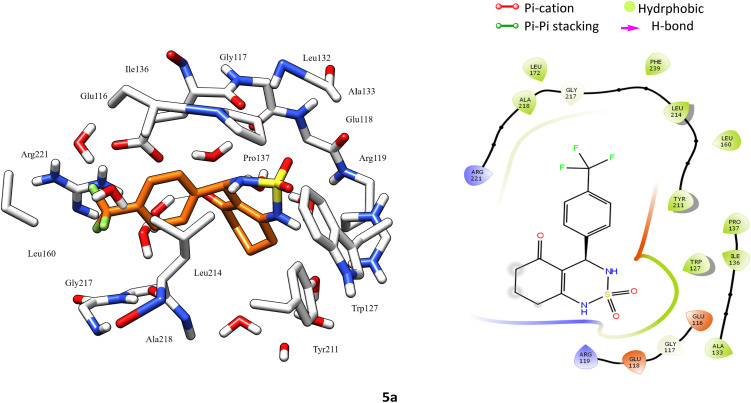
3D left and 2D right binding disposition of compound 5a after docking calculations in the active site of Eg5 enzyme. The amino acid residues were shown as stick model and H-bonds were shown as black lines.

After analyzing the other compounds, we distinguish that the substituent type on the phenyl ring plays a crucial role in the stability within the active site, in which compounds with a steric substituent at position *para* on the aromatic ring provoke medium stability compared with enastron inside the pocket, as is the case of 6a, 7a, and 8a compounds with NO_2_, Br, Cl substituent (Fig. 6).

Docking analysis revealed that the synthesized compounds interact with Eg5 in good manner and confirms the importance of H-bond donor group in cyclosulfamide ring, moreover the nature and position of substituent on the phenyl ring.

The results of the molecular docking studies support and provide an explanation for the biological test results.

#### Structure–activity relationship (SAR)

All the investigated compounds are analogues of enastron, wherein the thione group of enastron has been replaced by a sulphone group. The introduction of a sulfonamide group (NH–SO_2_) serves as a bio-isostere of the amide group (NH–CO) and the thioamide group (NH–CS).^44^ This substitution offers several advantages, including increased potency of inhibition and reduced toxicity. The sulfonamide group possesses also the ability to act as both a hydrogen donor and acceptor, thereby enhancing its pharmacological properties.^45^

**Fig. 7:**
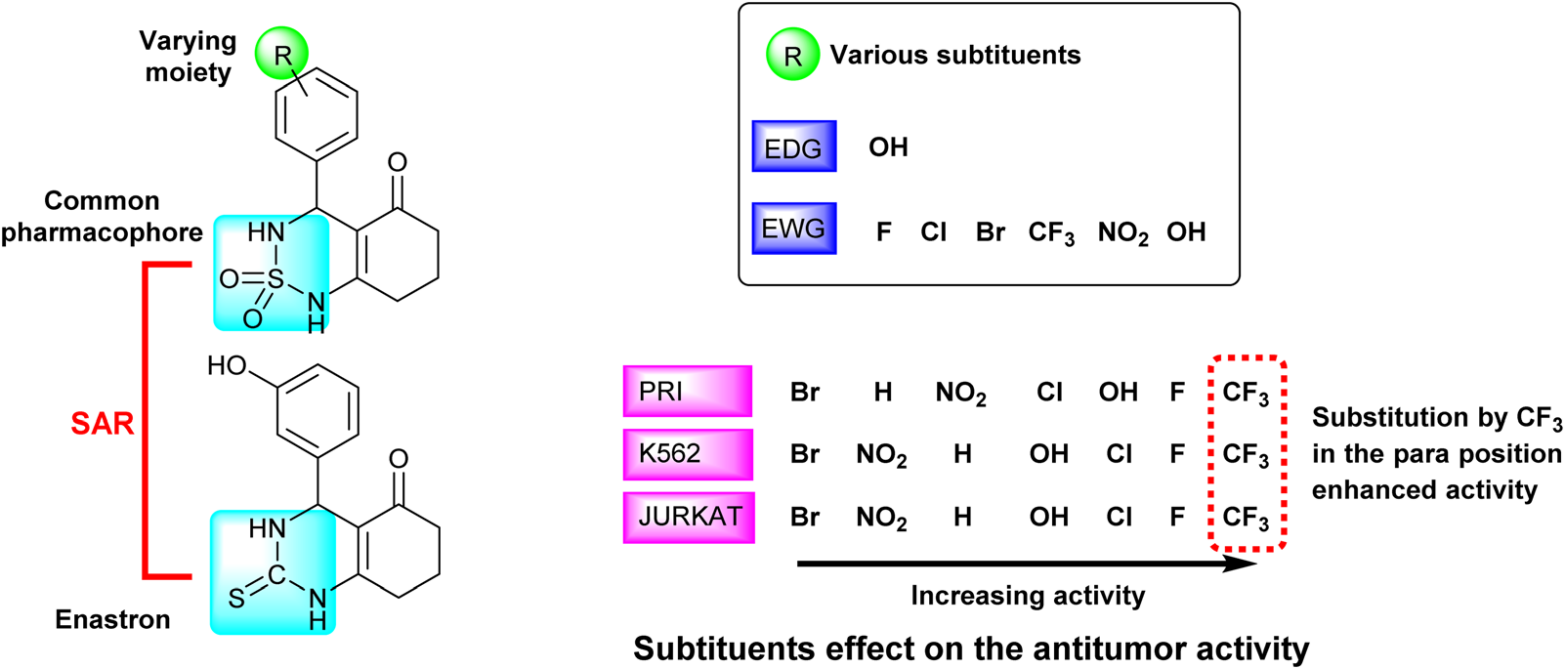
Schematic representation of SAR and substituents effect on the antitumor activity.

The studied derivatives share the sulfonamide group as the common pharmacophore, the latter is linked to a cyclic system. The different positions and substituents on the aromatic ring have a notable influence on the biological activity and docking scores. Based on the obtained results, it was observed that the incorporation of a fluoride substituent enhanced the inhibition of all three tested cell lines. Fig. 7 effectively illustrates the effect of substituents on the biological activity.

#### Molecular dynamics simulation

The complex structure containing the 4a bound with Eg5 enzyme was taken to perform the molecular dynamics (MD) simulation. The MD simulation was conducted using the free Desmond academic software-2022 and OPLS-2005 force field implemented on the LINUX environment's Intel Xeon Octa-Core 2.88 MHz. The 4a–Eg5 complex was solvated in an orthorhombic box with a dimension of 10.0 × 10.0 × 10.0 nm^3^. The simulation box complex contains 13 689 TIP3 water molecules and 46 353 atoms. The Na^+^ replaced five water molecules to neutralize the net charge of the complex system 4a–Eg5. All protein atoms were maintained at a distance equal to 1.0 nm from the solvate box edges. The solvated system was subjected to energy minimization of 50 000 steps. After that, the minimized system was equilibrated for 100 ps at 310 K. Following equilibration, the system was subjected to a final production run of 100 ns MD simulations at 310 K temperatures. Periodic boundary conditions were applied under isothermal and isobaric conditions with a relaxation time of 0.2 ps.^46^ MD simulation contributes to identifying the regions of the protein influenced by the presence of the ligand. These methods explicitly consider the whole protein–ligand system as flexible and can therefore be used for the validation of poses obtained by docking.^47^

To this end, we carried out a MD simulation to confirm the previous mode of binding predicted by the molecular docking for compound 4a fitted in the active site of the kinesin spindle Eg5.

Thus, we evaluated the stability of the system by the variations of the RMSD values, the number of hydrogen bonds between the ligand 4a and the kinesin spindle Eg5, the root mean square fluctuation (RMSF) and monitoring the protein secondary structure elements (SSE) like alpha-helices and beta-strands throughout the simulation. In the literature, MD stability can be also analyzed by the radius of gyration and DSSP.^48^

**Fig. 8:**
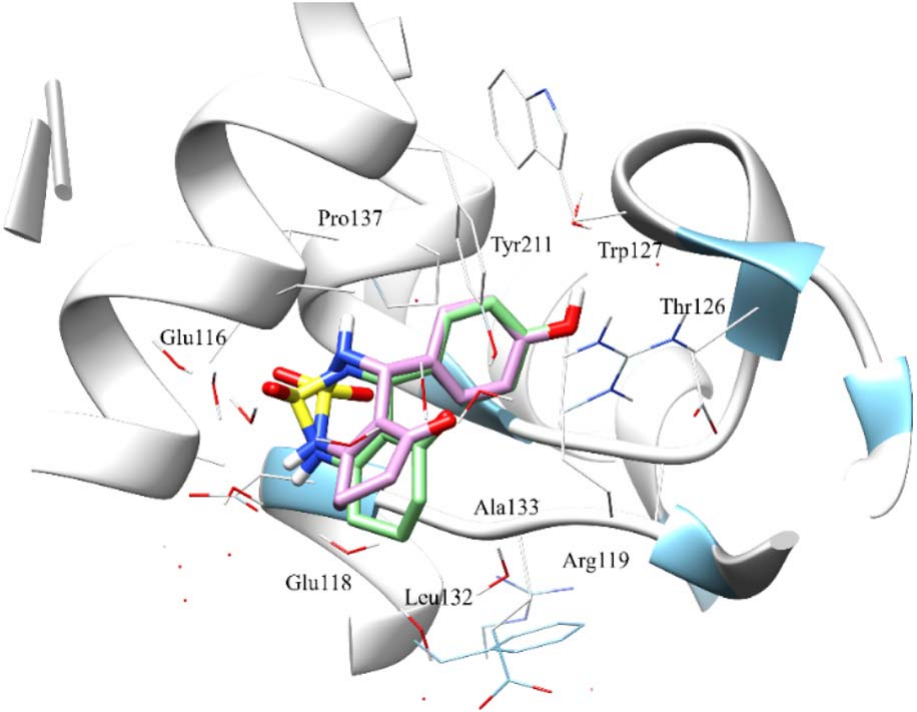
Superimposition of the docked compound 4a before MD simulation (0 ns) and after 100 ns running dynamic simulation in the active site. Compound 4a at 0 ns (green sticks) and 100 ns (pink sticks). The amino acid residues were shown as wire models.

The Fig. 8 shows the superimposition of the conformation resulting from the docking of the complex (4a–Eg5) before and after 100 ns of MD simulation. Analysis of the results shows that the ligand 4a retained the same binding site (before and after the MD simulation). In addition, the hydrogen bonds between Glu116, Tyr211, Glu118, Asp130 and residues, as well as all hydrophobic interactions with Leu132, Ala218, Pro137, and Ala133 residues, are retained.

##### Root mean square deviation (RMSD)

**Fig. 9:**
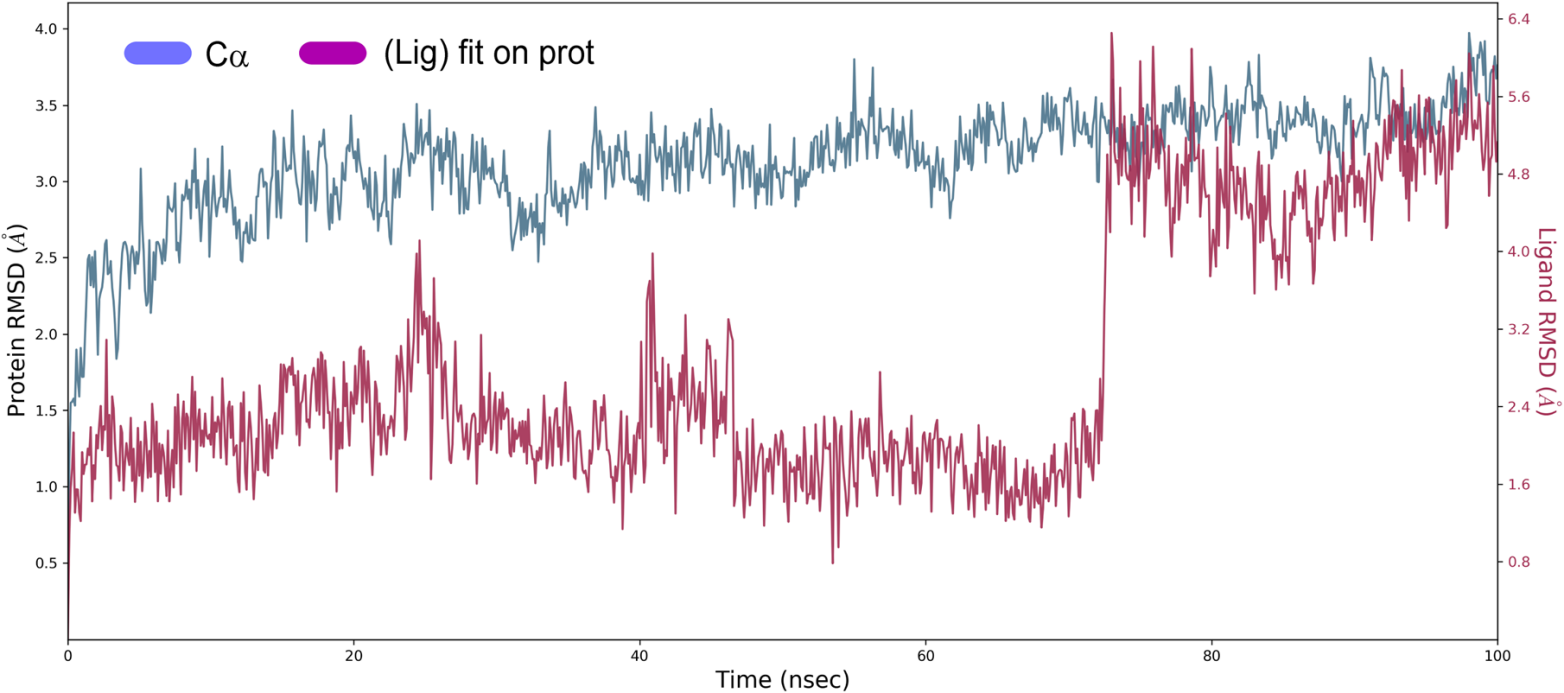
Protein and ligand root mean square deviation (PL-RMSD) obtained from the MD simulation trajectories.

The structural changes, specifically the deviation between the two compositions, can be best interpreted by measuring the protein and ligand PL-RMSD (Protein and Ligand Root Mean Square Deviation) obtained from the MD simulation trajectories. Changes in the order of 1–3 Å are perfectly acceptable for small molecules.^49^ The PL-RMSD measures the scale of distance between the protein and its ligand throughout the simulation time. All protein frames (1000 frames) are first aligned on the reference frame backbone (frame 0), and then the RMSD is calculated based on the C-alpha. Monitoring the RMSD of the protein can provide insights into the structural conformations throughout the simulation. It can indicate if the simulation is equilateral, and its fluctuations throughout the simulation are around some thermal average. Thus, a high RMSD value indicates the instability of the protein system. The comparative RMSD pattern analysis between the ligand 4a bound to the Eg5 protein demonstrates that both compositions showed a comparable RMSD value from the first nanosecond of the MD simulation trajectory. It's well established in MD literature that simulation time could extend until the system reached its equilibrium or when its main properties achieve stability.^50^ From the protein and ligand root-mean square deviation PL-RMSD data, we noted that all the systems have reached a plateau and equilibrated after 70 ns (Fig. 9). Then, we defined our productive phase into time intervals from 70 to 100 ns for all the simulations. After 70 ns of MD simulation, the ligand-RMSD values observed are significantly the same as the RMSD of the protein, indicating how stable the ligand is with respect to the protein and its binding pocket. The result of the PL-RMSD values indicated that has periodic backbone stability throughout the MD simulation while bound with the ligand 4a.

##### Root mean square fluctuation (RMSF)

**Fig. 10:**
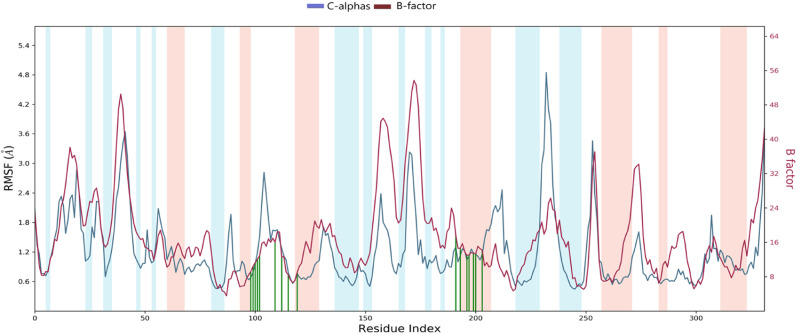
RMSF diagram of the amino acids of the Eg5 protein in the system studied 4a–Eg5 (protein residues that interact with the ligand are marked with green-colored vertical bars alpha-helical and beta-strand regions are highlighted in red and blue background).

The RMSF is a crucial result for measuring the deviation from a reference position of a ligand atom during the simulation time. This parameter represents the average deviation for each residue compared to the same atoms of the reference structure. Indeed, Fig. 10 shows the RMSF diagram of the amino acids of Eg5 protein in the system studied 4a–Eg5. The results were compared with the atomic displacement factor “*B* factor” of the Cα atoms of each protein residue. On this plot, peaks indicate areas of the protein that fluctuate the most during the simulation. In addition, we observe that the tails (N- and C-terminal) fluctuate more than any other part of the protein. Secondary structure elements like alpha helices and beta strands (highlighted in red and blue backgrounds, respectively) are usually more rigid than the unstructured protein part and thus fluctuate less than the loop regions (white backgrounds). The RMSF monitoring revealed that the residues: Leu30 (2.33 Å), Ala37 (2.42 Å), Asp59 (3.91 Å), Ala74 (2.06 Å), Thr107 (1.97 Å), Asn122 (2.82 Å), Ser178 (2.39 Å), Pro188 (3.88 Å), Ala230 (2.45 Å), Ile250 (4.85 Å), and Asn271 (3.45 Å) have higher RMSF values of alpha carbons (Cα). Therefore, the comparison of the RMSF curve of the alpha carbons (Cα) of the complex with the curve of the atomic displacement factor “*B*-factor” indicates a good simulation since the curve is parallel to that “*B*-factor” throughout the 100 ns MD simulation.

##### Protein–ligand contacts analysis

Protein–ligand interactions or ‘contacts’ are categorized into four types: hydrogen bonds, hydrophobic interactions, ionic, and water bridges.

##### Hydrogen bonds

**Fig. 11:**
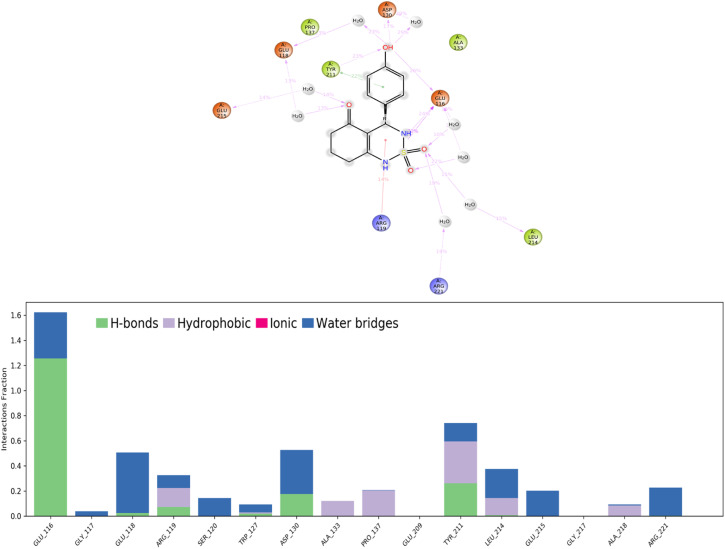
Protein–ligand contacts obtained from the MD simulation trajectories.

(H-bonds) play a critical role in ligand binding. Therefore, consideration of hydrogen-bonding properties in drug design is essential because of their strong influence on drug specificity, metabolization, and adsorption.^51^ In our study, hydrogen bonds formed between the 4a ligand and the Eg5 enzyme during the 100 ns of MD simulation are presented in Fig. 11.

The results show that the ligand formed 3 to 5 hydrogen bonds on average during the 100 ns of MD simulation which can be broken down into four subtypes: backbone acceptor (Glu116 128%); backbone donor (Tyr211 25.6%); side-chain acceptor (Asp130 17.7%).

Thus, supporting the results already obtained by the molecular docking, having predicted three hydrogen bonds formed between the 4a ligand with residues in the active site of the Eg5 enzyme.

##### Hydrophobic contacts

Fall into three subtypes: π–cation, π–π stacking, and other non-specific interactions. Generally, these types of interactions involve a hydrophobic amino acid and an aromatic or aliphatic group on the ligand. The results show that the ligand formed six hydrophobic contacts on average during the 100 ns of MD simulation, which can be broken down into three subtypes: π–cation (Arg119 15.10%), π–π stacking (Tyr211 22.1%), and other non-specific interactions (Ala133, Pro137, Tyr211, Leu214, Ala218).

##### Ionic interactions

Polar interactions are between two oppositely charged atoms within 3.7 Å of each other and do not involve a hydrogen bond. The obtained results of this study revealed that there are no ionic interactions between the ligand and the protein.

##### Water bridges

These are hydrogen-bonded protein–ligand interactions mediated by a water molecule. The hydrogen-bond geometry is slightly relaxed from the standard H-bond definition. The results show that the ligand formed multiple water bridges, especially between the Glu116, Glu118, Asp130, Leu214, and Glu215 amino acids and the 4a ligand.

**Fig. 12:**
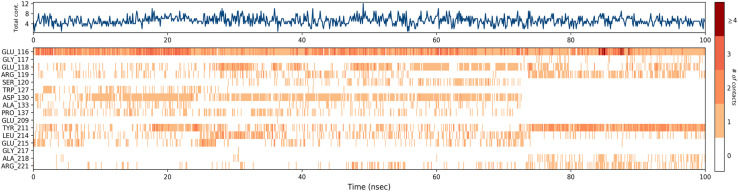
Timeline representation of the interactions and contacts (H-bonds, hydrophobic, ionic, and water bridges) obtained from the MD simulation trajectories.

A timeline representation of interactions and contacts (H-bonds, hydrophobic, ionic, and water bridges) is summarized in the Fig. 12. The top panel shows the total number of specific interactions the Eg5 protein makes with the ligand 4a over the 100 ns MD simulation trajectory. The bottom panel shows which residues interact with the ligand in each trajectory frame. Some residues, specifically: Glu166, Glu118, Asp130, Tyr211, Leu214 and Glu215, make more than one specific interaction with the ligand, which is represented by a darker orange shade, according to the scale to the right of the plot.

##### Protein secondary structure elements (SSE) analysis

**Fig. 13:**
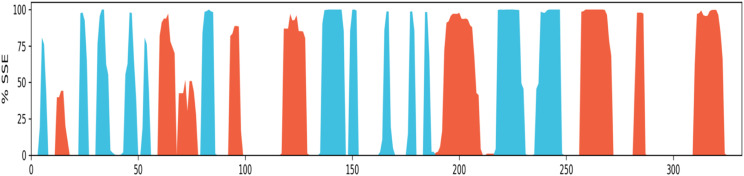
Protein secondary structure elements (SSE) monitored throughout MD simulation: alpha-helical 22.10%, beta-strand 20.85%, total SSE 42.95%. (*Alpha-helical and beta-strand regions are highlighted in red and blue backgrounds, respectively, and the loop regions are highlighted in white backgrounds*).

Protein secondary structure elements (SSE) like alpha-helices and beta-strands are monitored throughout the simulation. It is observed that the secondary structure elements (helix and beta-sheets) remained conserved throughout the simulation process (Fig. 13), which highlights the stability and reliability of the Eg5 after binding to the ligand. A bit of fluctuation is observed in the loop regions, but structurally, no significant changes have been seen. It has been also observed that the ligand is permanently attached to the active site without any structural modification, which implies that the ligand is highly stable. The secondary structural analysis has shown no significant overall conformational change in the complex.

#### Density functional theory (DFT) study

The stability and reactivity of studied benzothiadiazinone dioxides are frequently linked to their molecular geometry and substituent types. To establish and evaluate the correlation between the molecular docking results and the structure of studied compounds, a DFT investigation was conducted using Gaussian 09.

**Fig. 14:**
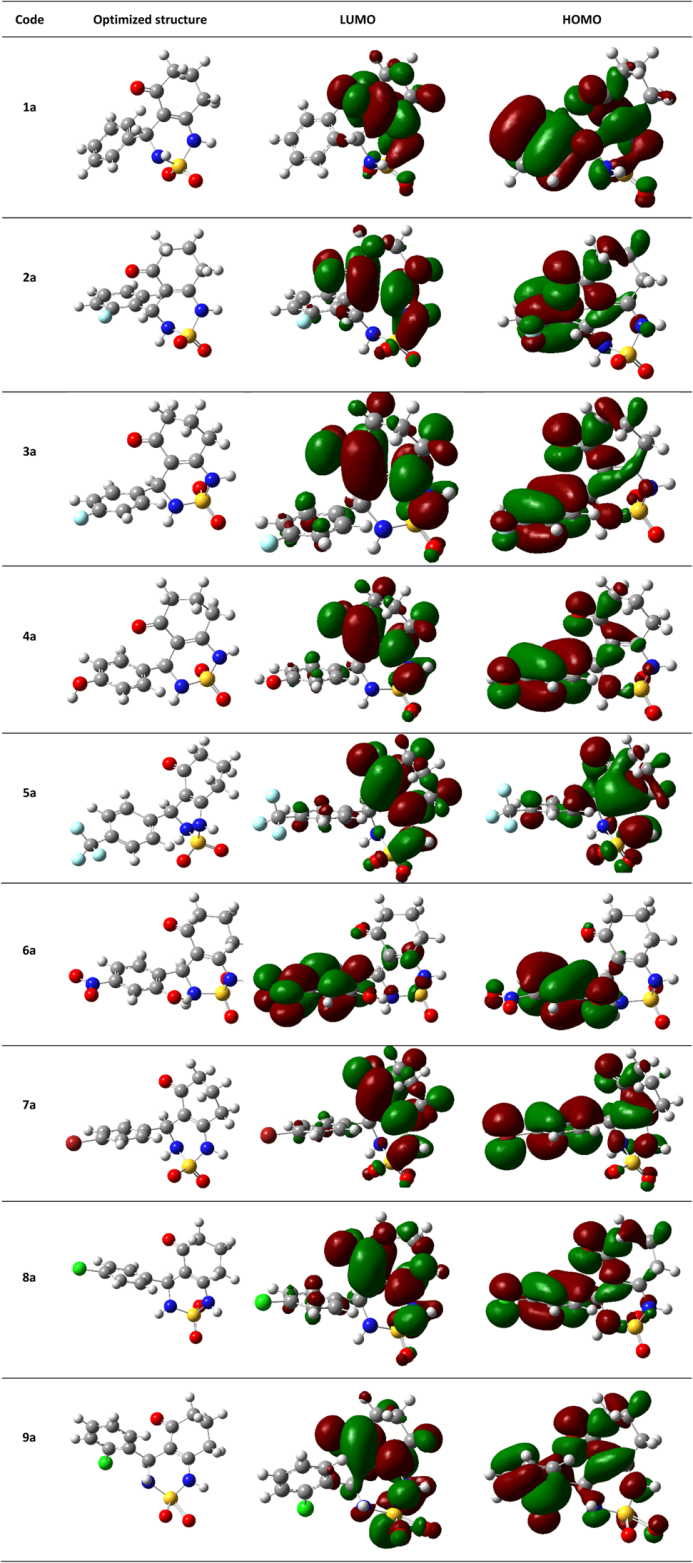
(HOMO, LUMO) orbitals and optimized structures (1a–9a) at B3LYP/6-31G(d,p) level using a contour threshold of 0.02 a.u in gas phase.

The theoretical DFT calculations were performed in gas phase using B3LYP/6-31G(d,p) basis set method. All optimum compounds are stable, and this is approved in terms of the absence of the imaginary frequency (Fig. 14).

The highest occupied molecular orbital (HOMO) and the lowest unoccupied molecular orbital (LUMO) are collectively referred to as frontier molecular orbitals (FMOs). The HOMO is occupied by electrons and has the highest energy level, making it an electron donor, while the LUMO is unoccupied and has the lowest energy level, allowing it to accept electrons, making it an electron acceptor. These FMOs are crucial in determining how ligands interact with their receptors. By providing qualitative information on the susceptibility of electrons in the HOMO to transfer to the LUMO, FMOs can aid in understanding the nature of these interactions. HOMO and LUMO are important quantum chemical parameters that determine the reactivity of molecules and are used to calculate various chemical reactivity descriptors.

All necessary parameters to study the chemical reactivity of synthesized compounds including energy gap, kinetic stability, chemical softness, chemical hardness (*η*), electronic chemical potential (*μ*), electronegativity (*χ*), electrophilicity index (*ω*), and hydrophobicity coefficient (Log *P*) were calculated and listed in Table 4. Additionally, Fig. 14 shows the frontier molecular orbital HOMO and LUMO for the investigated compounds in gas phase.

**Table:** The calculated parameters of studied compounds obtained by B3LYP/6-31G(d,p) method in gas phase

Molecular descriptors	1a	2a	3a	4a	5a	6a	7a	8a	9a
Log *P*	−0.07	0.08	0.08	−0.046	0.85	−0.39	0.75	0.48	0.48
*α*_tot_ (bohr^3^)	165.36	167.95	167.95	210.13	178.93	214.13	196.88	181.19	185.16
*μ* (D)	4.8852	5.0287	4.0705	3.1712	5.7000	7.3096	4.9334	4.4620	5.1925
*E*_HOMO_ (eV)	−0.2382	−0.2367	−0.2361	−0.2191	−0.2470	−0.2439	−0.2379	−0.2384	−0.2367
*E*_LUMO_ (eV)	−0.0483	−0.0517	−0.0521	−0.0477	−0.0544	−0.0873	−0.0523	−0.0535	−0.0530
Δ*E*_gap_ (eV)	0.1899	0.1850	0.1841	0.1714	0.1926	0.1566	0.1856	0.1850	0.1837
(*η*)	0.0949	0.0925	0.0920	0.0857	0.0963	0.0783	0.0928	0.0925	0.0919
(*S*)	10.5330	10.8091	10.8666	11.6713	10.3853	12.7747	10.7770	10.8120	10.8867
(*μ*)	−0.1433	−0.1442	−0.1441	−0.1334	−0.1507	−0.1656	−0.1451	−0.1460	−0.1449
(*χ*)	0.1433	0.1442	0.1441	0.1334	0.1507	0.1656	0.1451	0.1460	0.1449
(*ω*)	0.1081	0.1124	0.1128	0.1039	0.1179	0.1752	0.1134	0.1152	0.1142

Based on the study, it appears that most of the compounds being analyzed have moderate lipophilicity with a Log *P* range of [−0.39 to 0.75]. However, one derivative, 5a, has the highest value of Log *P* at 0.85.

In terms of dipole moment, the range observed is [3.17–7.30], with compound 6a showing the highest value at 7.30. This is likely due to the presence of NO_2_ and OH substituents on the aromatic ring.

The polarizability depends on how the susceptibility of the molecular system electron cloud is affected by the approaching of a charge. Molecules of large size are more polarizable compounds. It is worth noting that compound 1a is the smallest in size and has the least polarizability (165.36 bohr^3^), however, 6a of the highest complexity is predicted to have the highest polarizability, 214.13 bohr^3^.

The results of the FMOs energy analysis revealed that the energies of compounds 6a and 4a are lower compared with the other compounds. However, the destabilization of the LUMO level is found to be higher in 6a than in the others.

The compound 5a showed the highest energy gap Δ*E*_gap_ = 0.1926 eV, it is the most stable of all studied compounds.

To ensure the validity of the evidence regarding the inhibitory properties of compounds, it is important to calculate the 3D plot molecular electrostatic potential (MEP). The MEP can provide insight into the size and shape of the electrostatic potential of a molecule, and can help predict physicochemical properties based on the molecular structure of the compounds being studied. Additionally, the MEP is a useful tool to explain hydrogen bonding and estimating a drug's reactivity towards electrophilic and nucleophilic attacks.

The MEP of compounds (1a–9a) was calculated using the same method and base sets. The MEP shows that the maximum negative region is the preferred site for electrophilic attacks, indicated by the red color. This means that an electrophile will be attracted to the negatively charged sites of the molecule. The opposite is true for the blue regions, where electrophiles will be repelled.

**Fig. 15:**
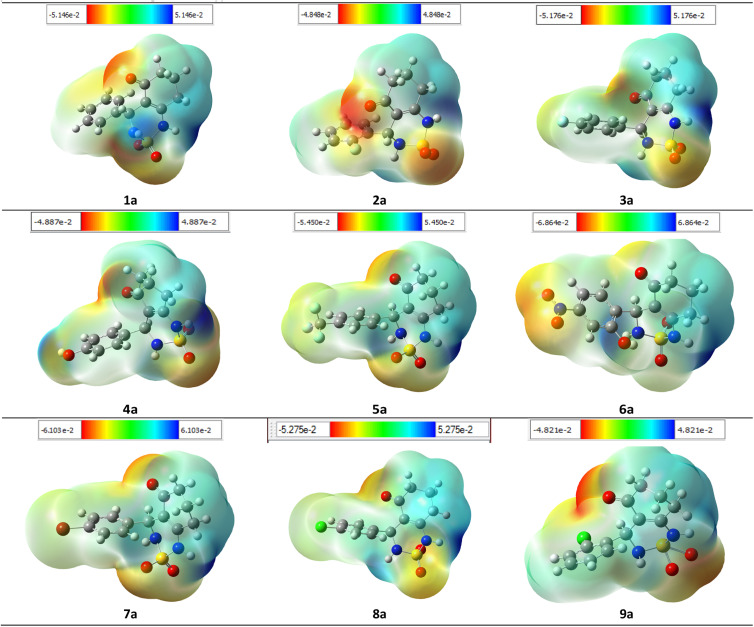
MEP formed by mapping of total density over electrostatic potential in gas phase for all compounds (1a–9a).

The mapping of the electrostatic potential around the compounds varied according to the type of atoms and their electronic nature. This variation can be responsible for differences in binding affinity with the active site receptor. Therefore, the MEP can be a useful tool in predicting a compound's inhibitory properties (Fig. 15).

#### *In silico* pharmacokinetics analysis

Before conducting *in vivo* testing, it is crucial to examine the pharmacokinetic properties of any molecule being considered as a potential drug candidate, including factors such as absorption, distribution, metabolism, excretion, and toxicity (ADME). The drug-likeness of synthesized compounds predicted by analyzing their ADME properties using SwissADME server based on Lipinski's rule of five. These essential parameters not only to determine the similarity of the substance to a drug, but also its effectiveness within the body.

The ability of a drug to reach the site of action is determined by its pharmacokinetic process, while the pharmacodynamic process assesses whether the drug can produce the desired pharmacological effect.

The Lipinski rule, also known as the rule of five, utilizes basic molecular descriptors formulated by Lipinski *et al.* to determine drug-likeness. According to this rule, most drug-like molecules possess a Log *P* value less than or equal to 5, a molecular weight less than or equal to 500 Da, and no more than 10 hydrogen bond acceptors and 5 hydrogen bond donors. Molecules that violate more than one of these criteria may face challenges with bioavailability. Physicochemical parameters play a vital role in generation and escalation of bioactivity of chemical entity.

The pharmacokinetic parameters of synthetic compounds are given in Table 5. Studied compounds (1a–9a) have molecular weight in the range of 278–357 (<500). Low molecular weight drug molecules (<500) are easily transported, diffused, and absorbed as compared to heavy molecules. Molecular weight is an important aspect in therapeutic drug action, if it increases correspondingly, it effects the drug action. Number of hydrogen bond acceptor and number of hydrogen bond donor in the tested compounds were found to be within Lipinski limit. H-bond acceptor ranged from 2 to 7 (<10) H-bond donor ranged from 1 to 3 (<5).

**Table:** Pharmacokinetic parameters and drug likeness score (DLS) of compounds (1a–9a)

Properties	1a	2a	3a	4a	5a	6a	7a	8a	9a	CLB
Molecular weight (g per mole)	278.33	296.32	296.32	294.33	346.32	339.32	357.22	312.77	312.77	304.21
Rotatable bonds	1	1	1	1	1	2	1	1	1	9
H-bond donor	2	2	2	3	2	3	2	2	2	1
H-bond acceptor	4	5	5	5	7	7	4	4	4	2
Violations	0	0	0	0	0	0	0	0	0	0
Log *P*_o/w_ iLOGP	1.17	1.47	1.29	1.03	1.61	0.51	1.87	1.67	1.57	4.62
Log *S* ESOL	−2.10	−2.26	−2.26	−1.97	−2.97	−2.04	−3.02	−2.70	−2.70	−2.44
GI	High	High	High	High	High	High	High	High	High	High
BBB	No	No	No	No	No	No	No	No	No	No
Log *K*_p_	−7.59	−7.62	−7.62	−7.93	−7.37	−8.33	−7.57	−6.79	−7.35	−6.95
Bioavailability score	0.55	0.55	0.55	0.55	0.55	0.55	0.55	0.55	0.55	0.85
TPSA (Å)	83.65	83.65	83.65	103.88	107.64	149.70	83.65	83.65	83.65	40.54
DLS	−0.78	−0.49	−0.46	−0.16	−0.66	0.07	−0.66	−0.27	−0.025	−0.66

Most compounds have one rotatable bond except compound 6a has two rotatable bonds (<10). Additionally, they showed good lipophilicity, as indicated by iLOGP values ranging from 0.51 to 1.87, which are less than <5 and they exhibited good water solubility, as indicated by Log *S* ESOL values ranging from −1.97 to −3.02.

The skin permeability coefficient *K*_p_ values were found ranged from −8.33 to −6.79. *K*_p_ is an important parameter in the development of topical drug formulations and is linearly correlated with molecular size and lipophilicity. The more negative the Log *K*_p_ (with *K*_p_ in cm s^−1^), the less skin permeant the molecule.

The ADME study revealed that none of the compounds violate the Lipinski's rule of five, which is a set of criteria used to assess the drug-likeness of a chemical compound based on its physicochemical properties, including molecular weight, lipophilicity, hydrogen bonding capacity, and solubility.

The possibility that a chemical compound possesses characteristics that make it an appropriate medication candidate is determined also by the drug likeness score (DLS). It is typically based on the compound's similarity to known drugs and their physicochemical properties. A high DLS indicates that a compound is more likely to have favorable pharmacokinetic and pharmacodynamic properties, and therefore more likely to be successful in clinical trials as a drug candidate.

The studied compounds present a remarkable DLS in the range of [−0.78 to 0.07] (Table 5). It was observed that compound 4a, which demonstrated the highest stability in the active site of the Eg5 enzyme (docking score: −6.95) exhibited a good drug likeness score value (DLS: 0.07) and the compound 6a showed the best DLS score 0.07. The bioavailability radar enables a rapid evaluation of a compound's drug-likeness by considering six physicochemical attributes: lipophilicity, size, polarity, solubility, flexibility, and saturation. The radar plot of the molecule had to fall totally within a specific physicochemical range on each axis, which was represented by a pink area, in order to be deemed drug-like.

**Fig. 16:**
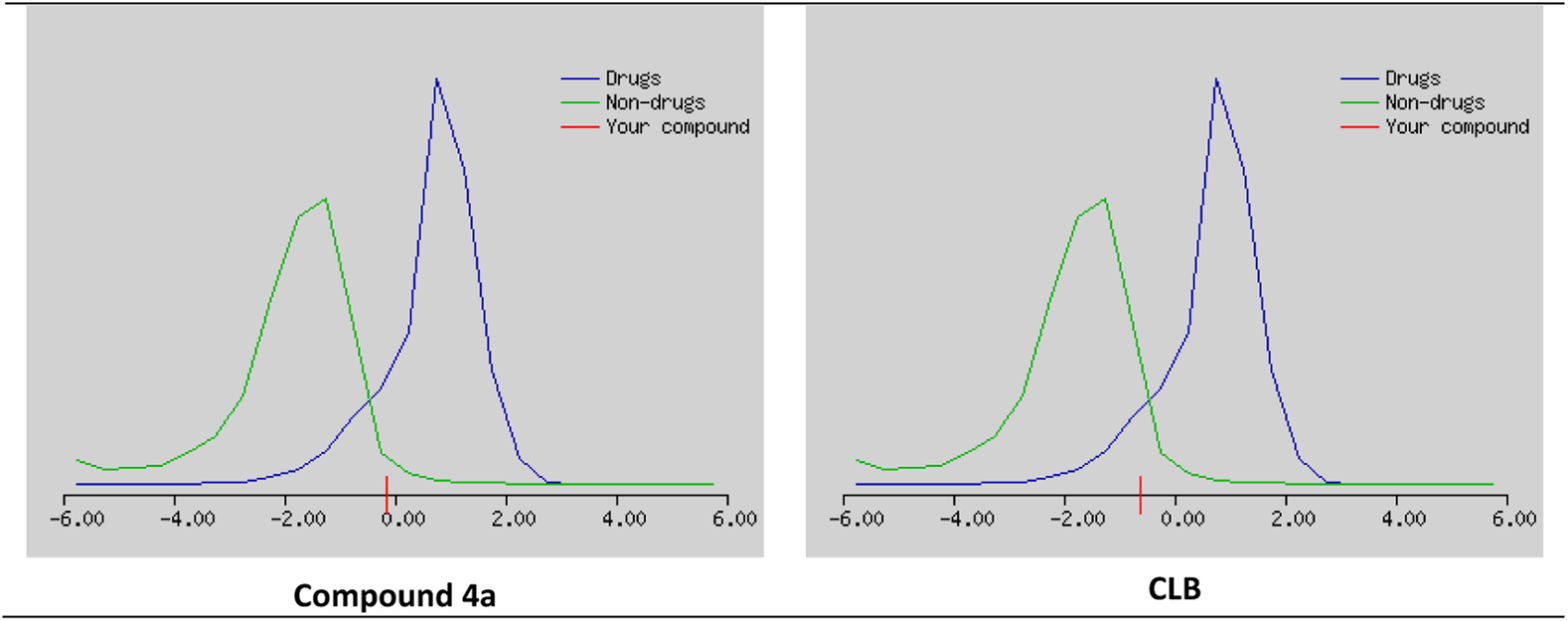
Drug likeness score estimation curve using Molsoft server.

This pink area represents the optimal range for each properties (lipophilicity: XLogP3 between −0.7 and +5.0, size: MW between 150 and 500 g mol^−1^, polarity: TPSA between 20 and 130 Å^2^, solubility: Log *S*) not higher than 6, saturation: fraction of carbons in the sp^3^ hybridization not less than 0.25, and flexibility: no more than 9 rotatable bonds (Fig. 16).

**Fig. 17:**
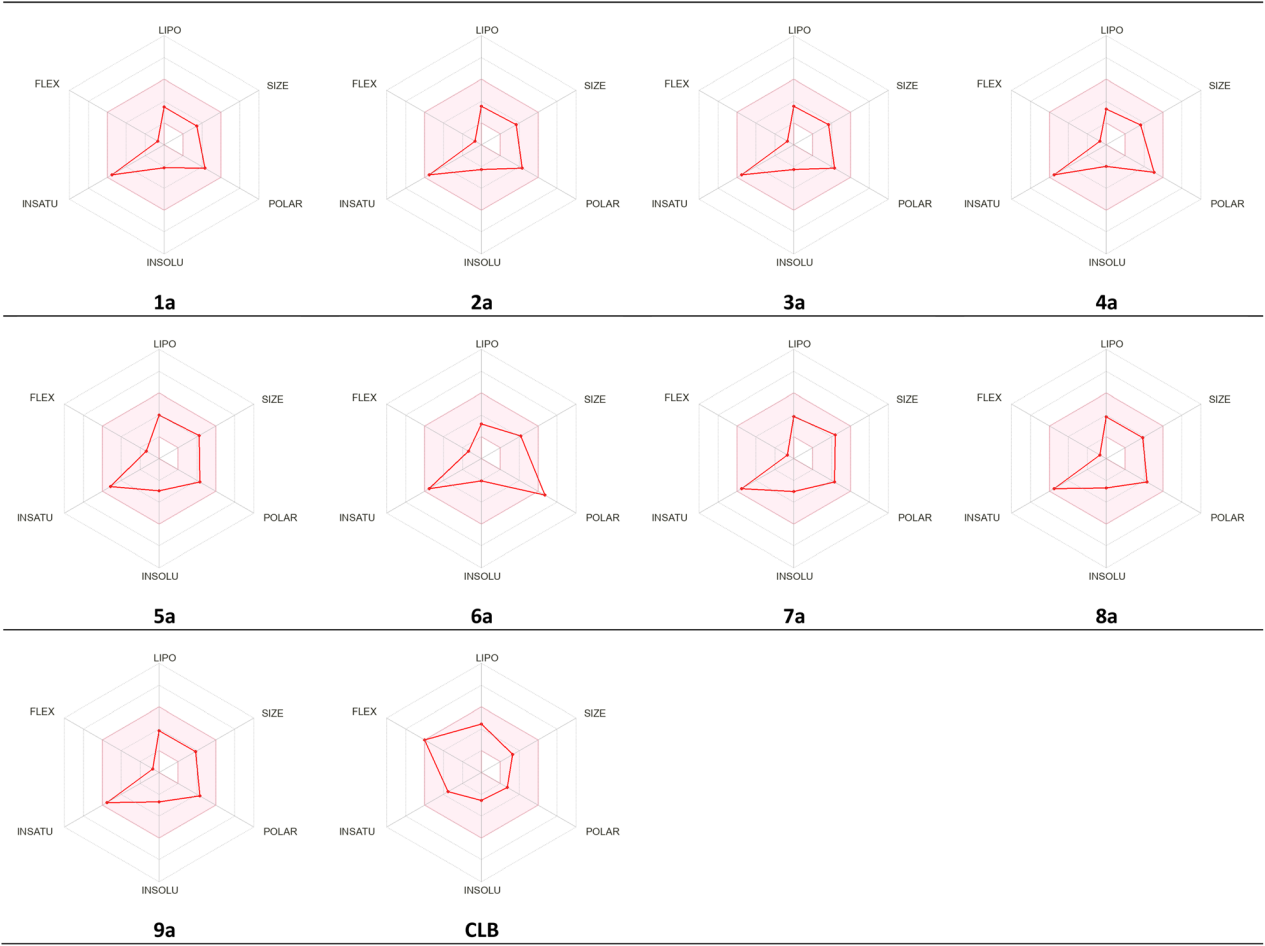
Radar related to physicochemical properties of the synthesized molecules and CLB drug.

As shown in Fig. 17, all studied molecules presented physico-chemical profiles which make them suitable for oral administration except the derivative 6a not orally bioavailable; because it showed a higher polarity (TPSA 149.7).

## Conclusions

In summary, a series of cyclosulfamide derivatives were evaluated for their antitumor activity against three human cell lines (PRI, K562, and JURKAT). The findings of the *in vitro* tests demonstrated that compound 5a displayed a significant inhibitory effect on the growth of these cancerous cell lines. The molecular docking study of the compounds in the active site of the Eg5 enzyme revealed high docking scores and good stability in the cavity for most of the compounds. These results suggest that the studied compounds could be potential candidates for inhibiting the Eg5 enzyme. Based on the positive results of the molecular docking study, we were motivated to confirm the predicted binding mode of compound 4a using MD simulation. The results of the MD simulation indicate that compound 4a maintained its binding site even after 100 ns, and the hydrogen bonds and hydrophobic interactions with the residues remained unchanged. DFT study enabled us to understand the electronic effects of the different substituted compounds. The MEP analysis allowed us to predict the most reactive sites for nucleophilic and electrophilic attacks. Furthermore, the analysis of the FMOs indicated that compound 5a had the highest energy gap, Δ*E*_gap_ = 0.1926 eV, making it the most stable among all compounds studied. All compounds were also evaluated based on the Lipinski rule of five for their bioactivity, molecular descriptors, and drug likeness and all of them exhibited favorable oral bioavailability.

## Author contributions

Abdeslem Bouzina: responsible for the principal idea and the synthesis of compounds. Yousra Ouafa Bouone: DFT study. Omar Sekiou: follow the calculation of MD simulation. Mohamed Aissaoui and Rachida Mansouri: studies for the molecular docking. Tan-Sothea Ouk: carried out the *in vitro* study. Abdelhak Djemel: *in silico* pharmacokinetics analysis. Noureddine Aouf, Zihad Bouslama and Malika Ibrahim-Ouali: editing and performing the manuscript preparation.

## Conflicts of interest

There are no conflicts to declare.

## Supplementary Material

RA-013-D3RA02904B-s001
