# Supplementary material for: In vitro antitumor activity, molecular dynamics simulation, DFT study, ADME prediction, and Eg5 binding of enastron analogues
Source: RSC Adv. 2023 Jun 28;13(28):19567–84. doi: 10.1039/d3ra02904b (PMC10301704; doi:10.1039/d3ra02904b)
Supplement: RA-013-D3RA02904B-s001 [file RA-013-D3RA02904B-s001.pdf]

Electronic Supplementary Material (ESI) for RSC Advances.
This journal is © The Royal Society of Chemistry 2023

In vitro antitumor activity, molecular dynamics simulation, DFT study, ADME prediction, and Eg5 binding of Enastron analogues

Abdeslem Bouzina,[a*] Yousra Ouafa Bouone,[a] Omar Sekiou,[b] Mohamed Aissaoui,[a] Tan-Sothea Ouk,[c] Abdelhak Djemel,[d] Rachida Mansouri,[b] Malika Ibrahim-Ouali,[e] Zihad Bouslama,[b] and Nour-Eddine Aouf[a]

[a]*Laboratory of Applied Organic Chemistry, Bioorganic Chemistry Group, Department of Chemistry, Sciences Faculty, Badji Mokhtar Annaba University, Box 12, 23000 Annaba, Algeria.*

[b]*Environmental Research Center (CRE), 23000 Annaba, Algeria.*

[c]*Université de Limoges, LABCiS, UR 22722, F-87000 Limoges, France.*

[d]*Research Unit in Medicinal Plants, URPM, 3000 Laghouat, attached to Research Center of Biotechnology, CRBt, 25000 Constantine, Algeria.*

[e]*Aix Marseille Univ, CNRS, Centrale Marseille, iSm2, Marseille F-13397, France*

*Corresponding author. Email: abdeslem.bouzina@univ-annaba.dz, bouzinaabdeslem@yahoo.fr

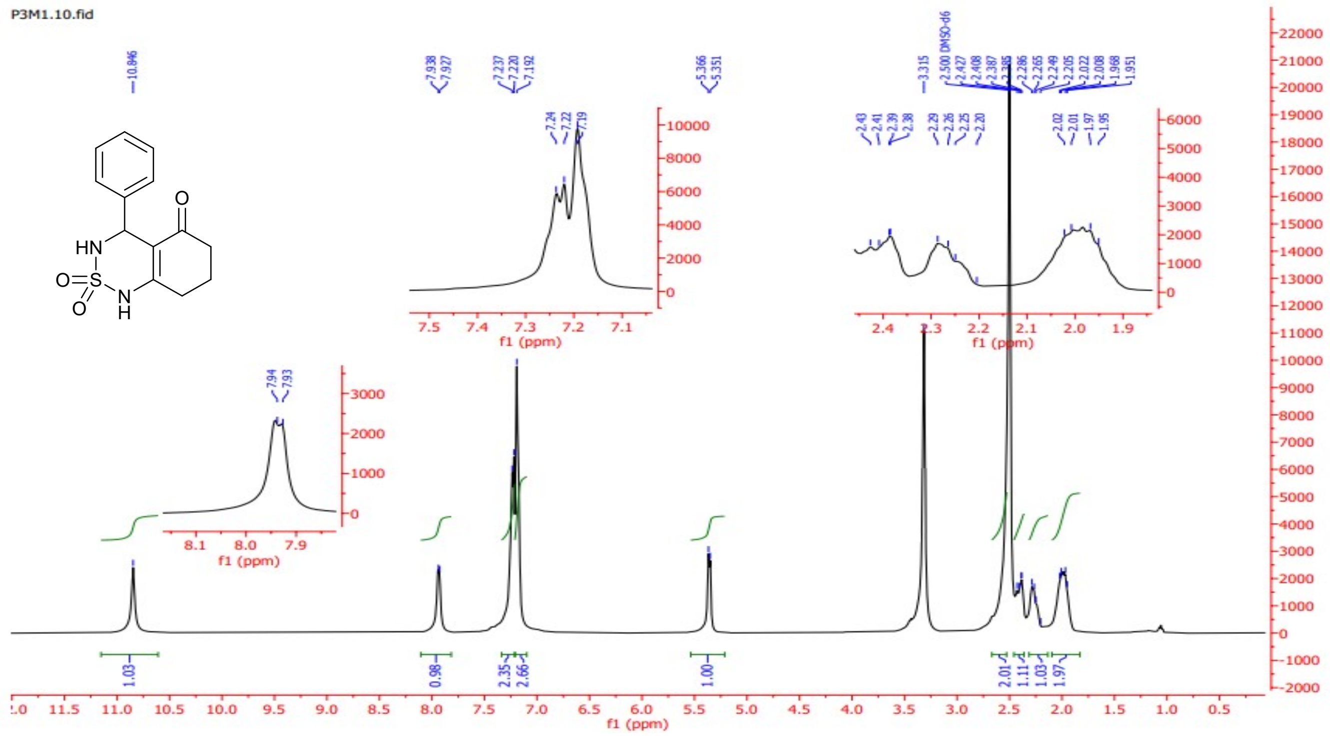

^{1}H NMR Spectrum: 4-phenyl-4,6,7,8-tetrahydro-1H-benzo[c][1,2,6]thiadiazin-5(3H)-one 2,2-dioxide

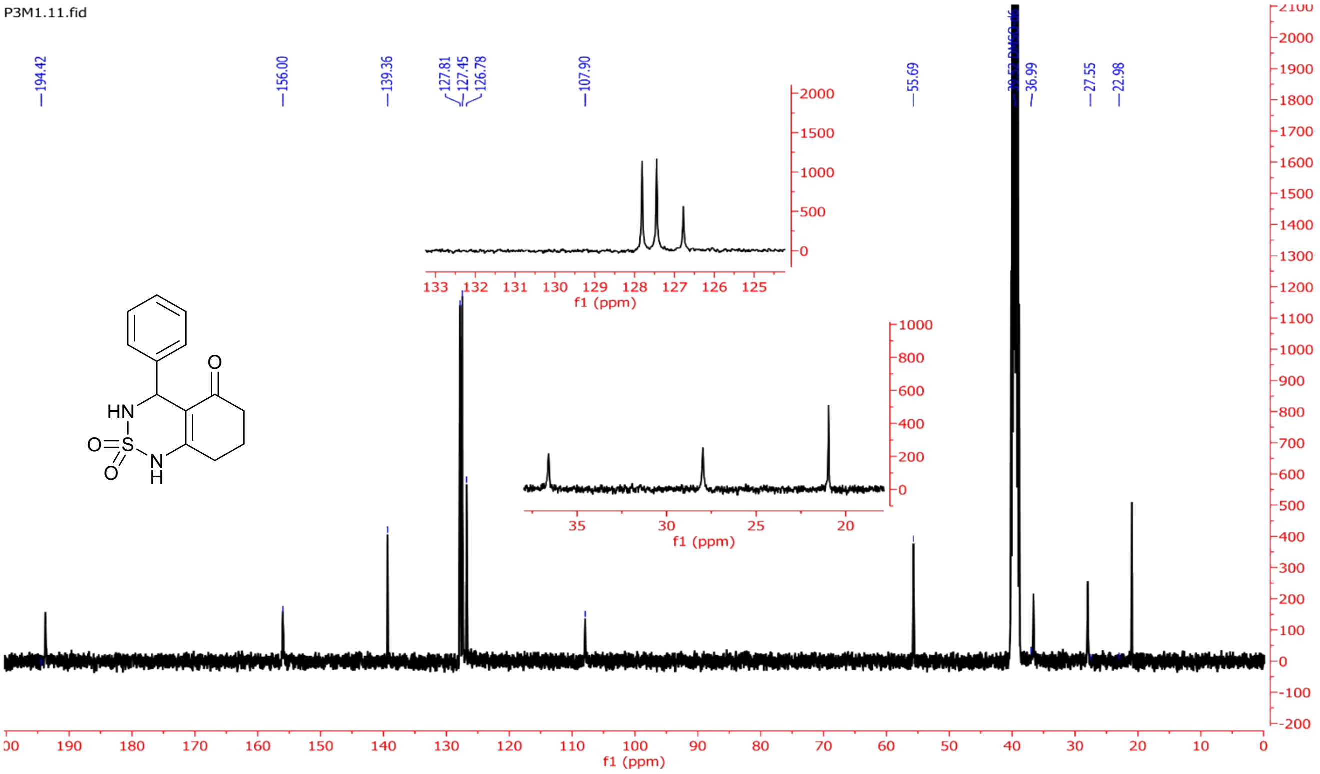

^{13}C NMR Spectrum: 4-phenyl-4,6,7,8-tetrahydro-1H-benzo[c][1,2,6]thiadiazin-5(3H)-one 2,2-dioxide

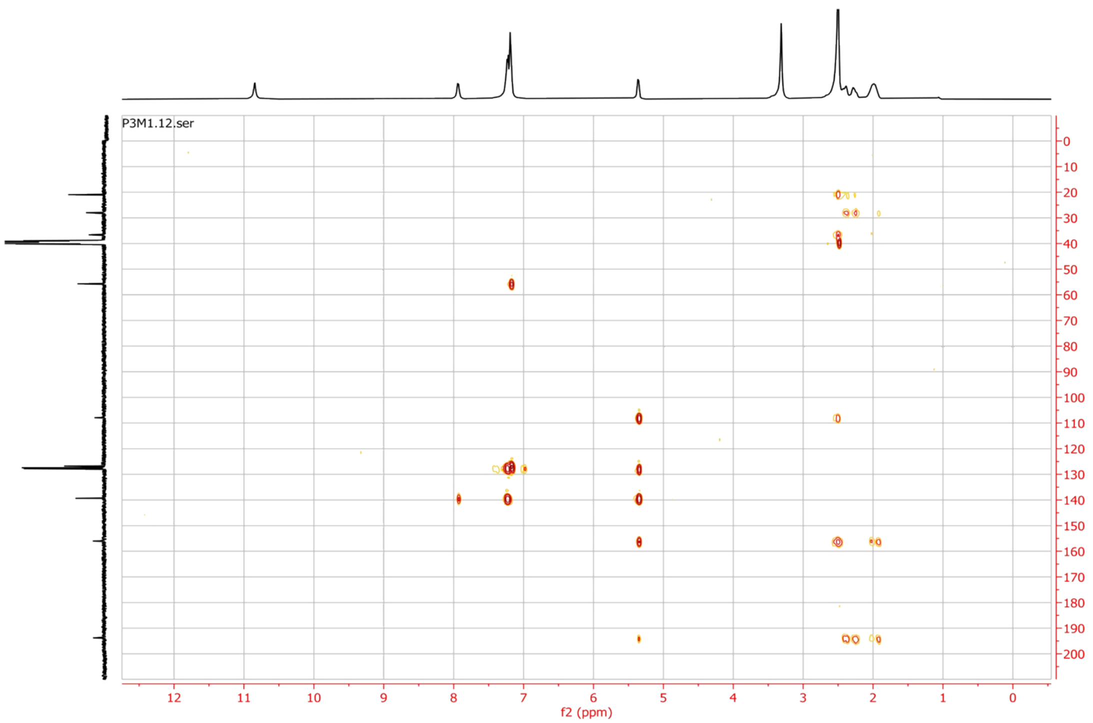

HMBC Spectrum: 4-phenyl-4,6,7,8-tetrahydro-1H-benzo[c][1,2,6]thiadiazin-5(3H)-one 2,2-dioxide

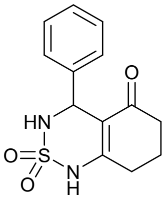

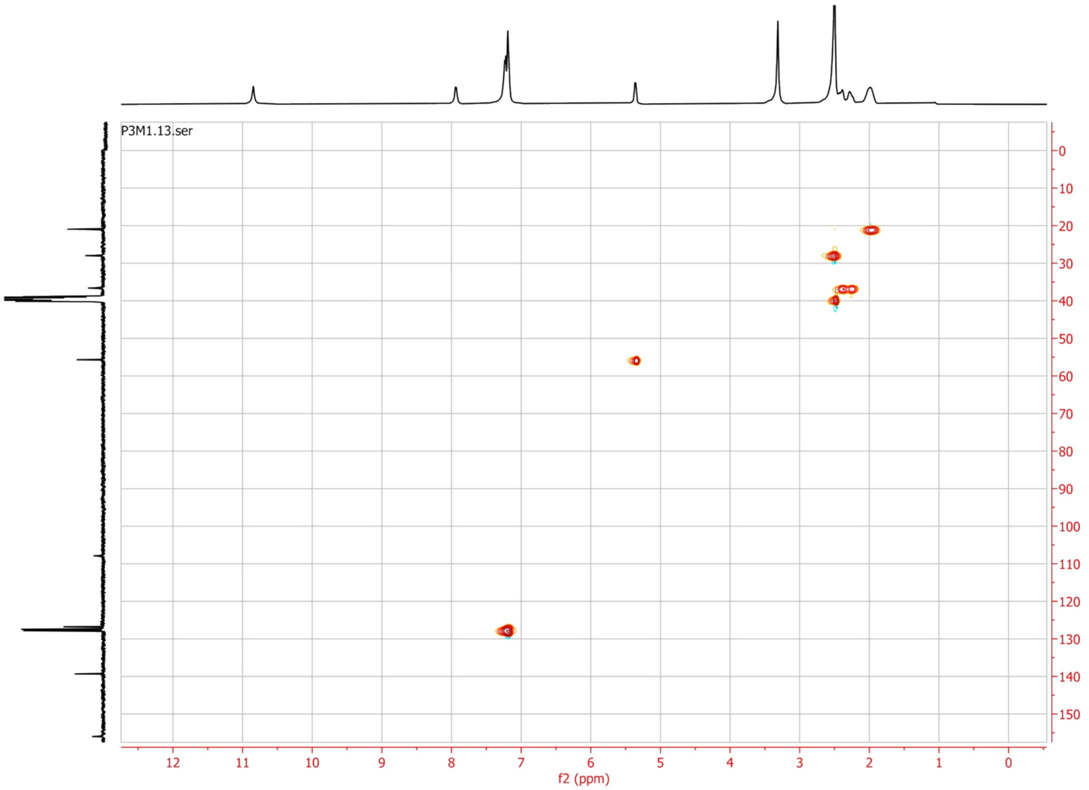

HMQC Spectrum: 4-phenyl-4,6,7,8-tetrahydro-1H-

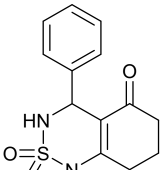

benzo[c][1,2,6]thiadiazin-5(3H)-one 2,2-dioxide

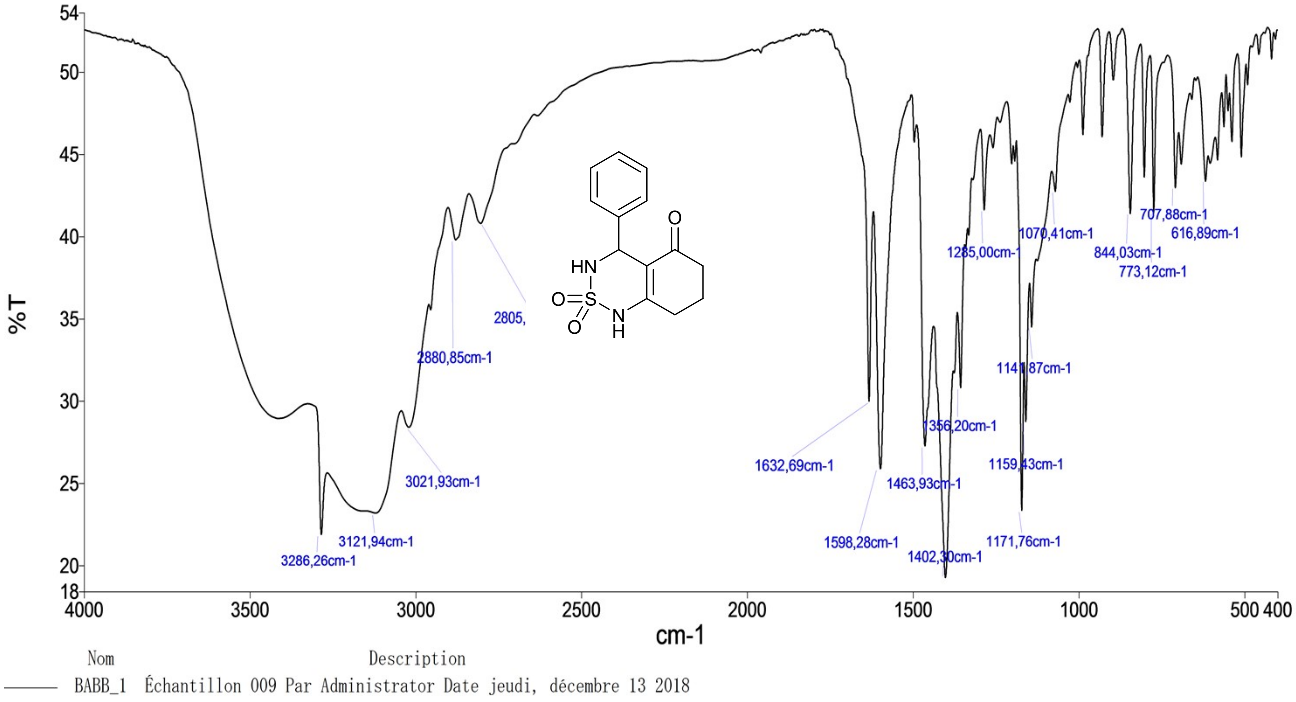

IR Spectrum: 4-phenyl-4,6,7,8-tetrahydro-1H-benzo[c][1,2,6]thiadiazin-5(3H)-one 2,2-dioxide

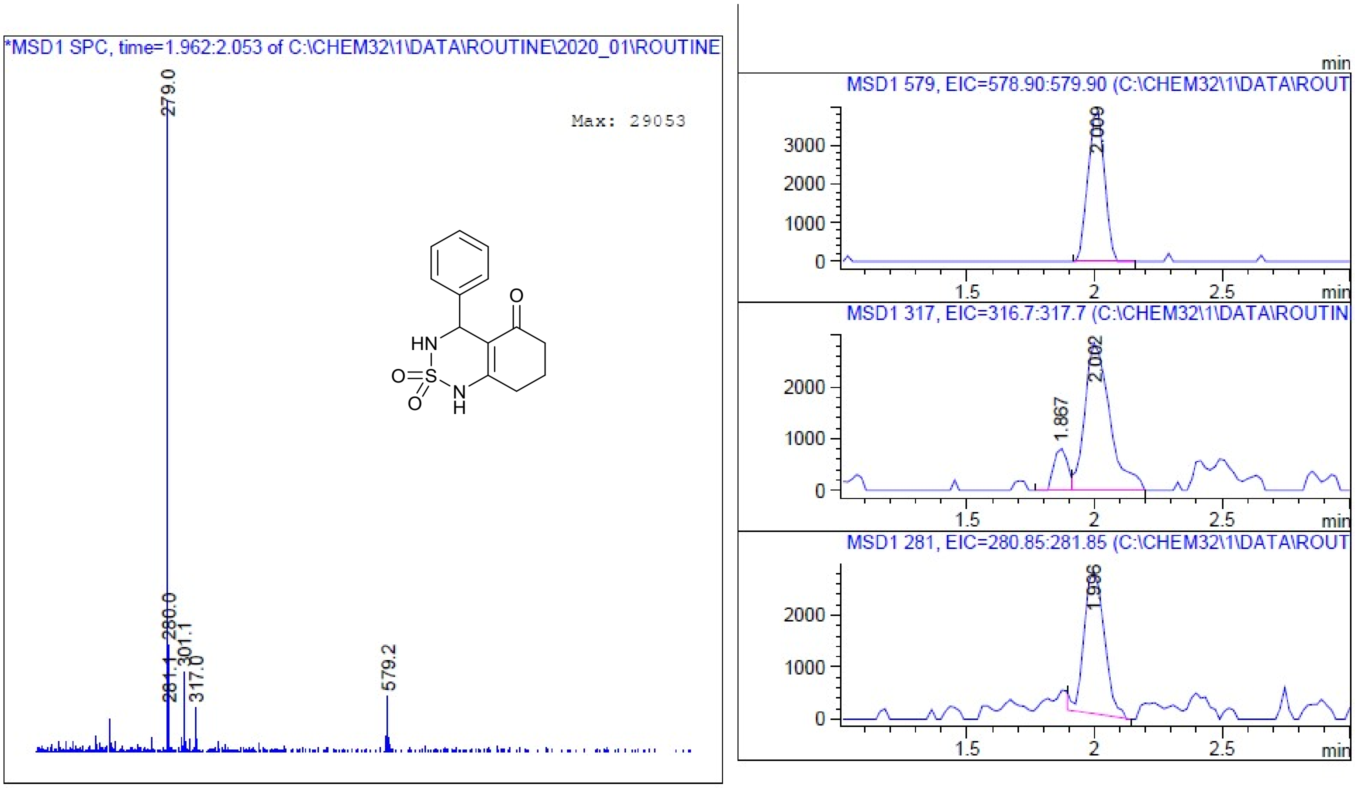

LC-MS: 4-phenyl-4,6,7,8-tetrahydro-1H-benzo[c][1,2,6]thiadiazin-5(3H)-one 2,2-dioxide

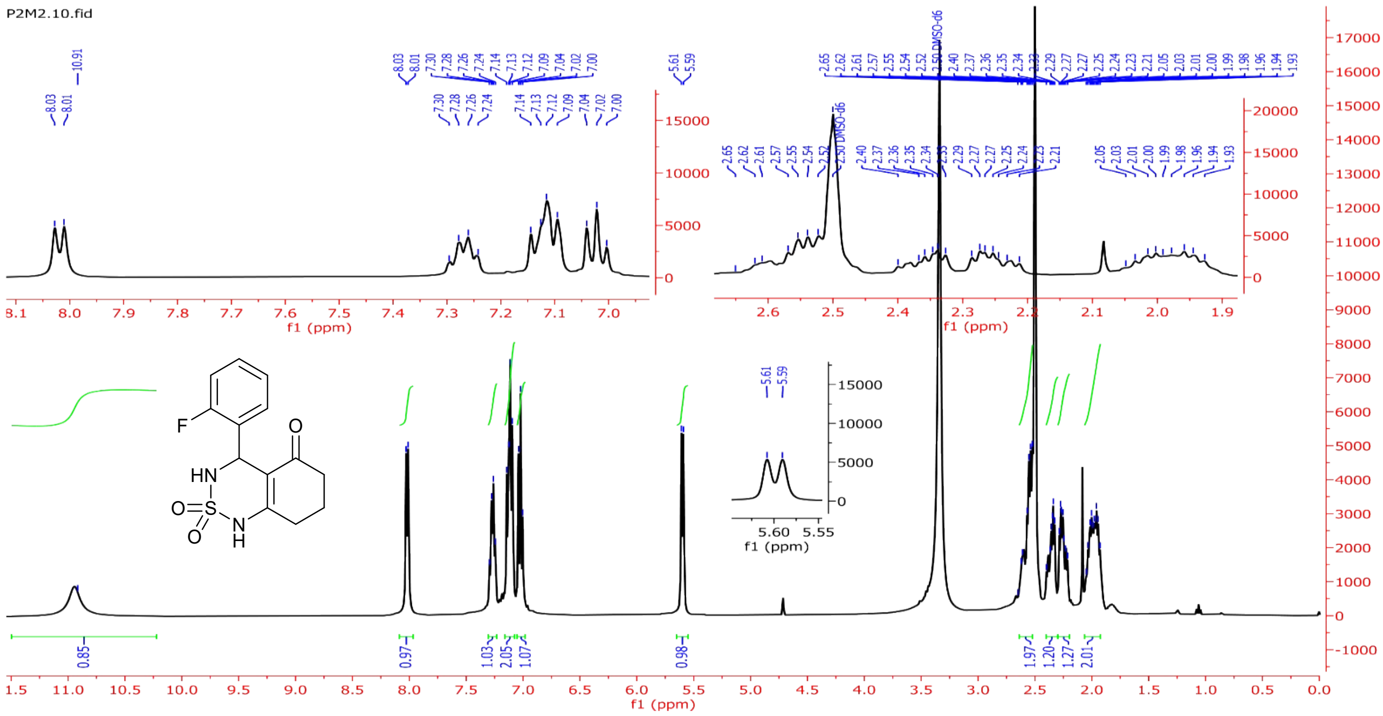

¹H NMR Spectrum: 4-(2-fluorophenyl)-4,6,7,8-tetrahydro-1H-benzo[c][1,2,6]thiadiazin-5(3H)-one 2,2-

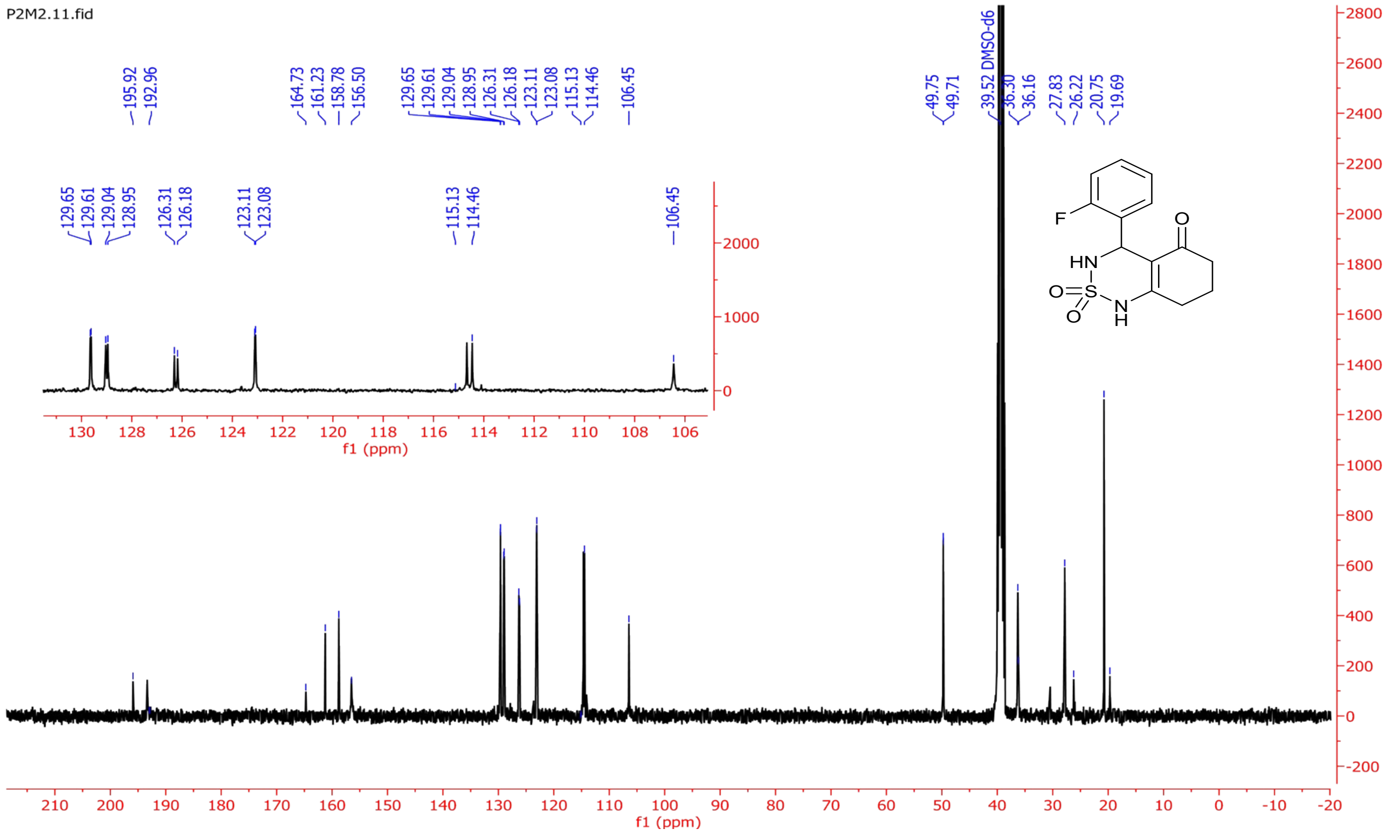

dioxide

^{13}C NMR Spectrum: 4-(2-fluorophenyl)-4,6,7,8-tetrahydro-1H-benzo[c][1,2,6]thiadiazin-5(3H)-one 2,2-dioxide

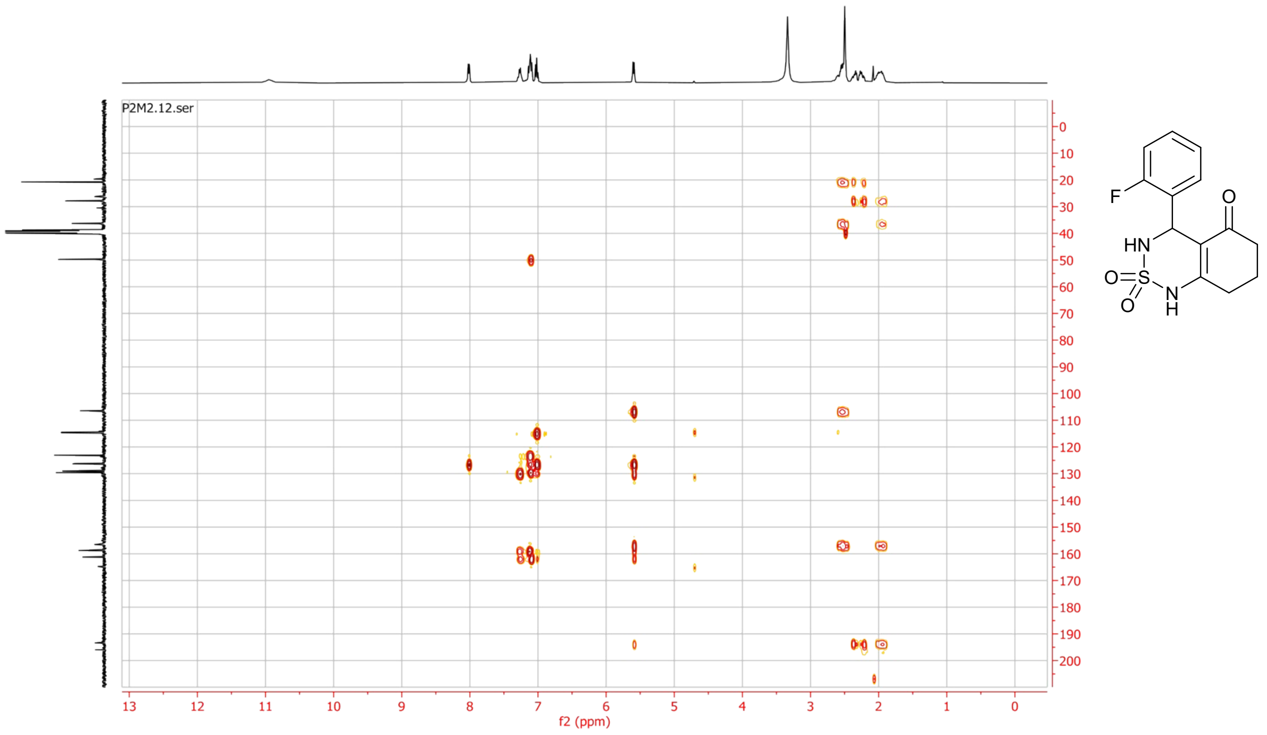

HMBC Spectrum: 4-(2-fluorophenyl)-4,6,7,8-tetrahydro-1H-benzo[c][1,2,6]thiadiazin-5(3H)-one 2,2-dioxide

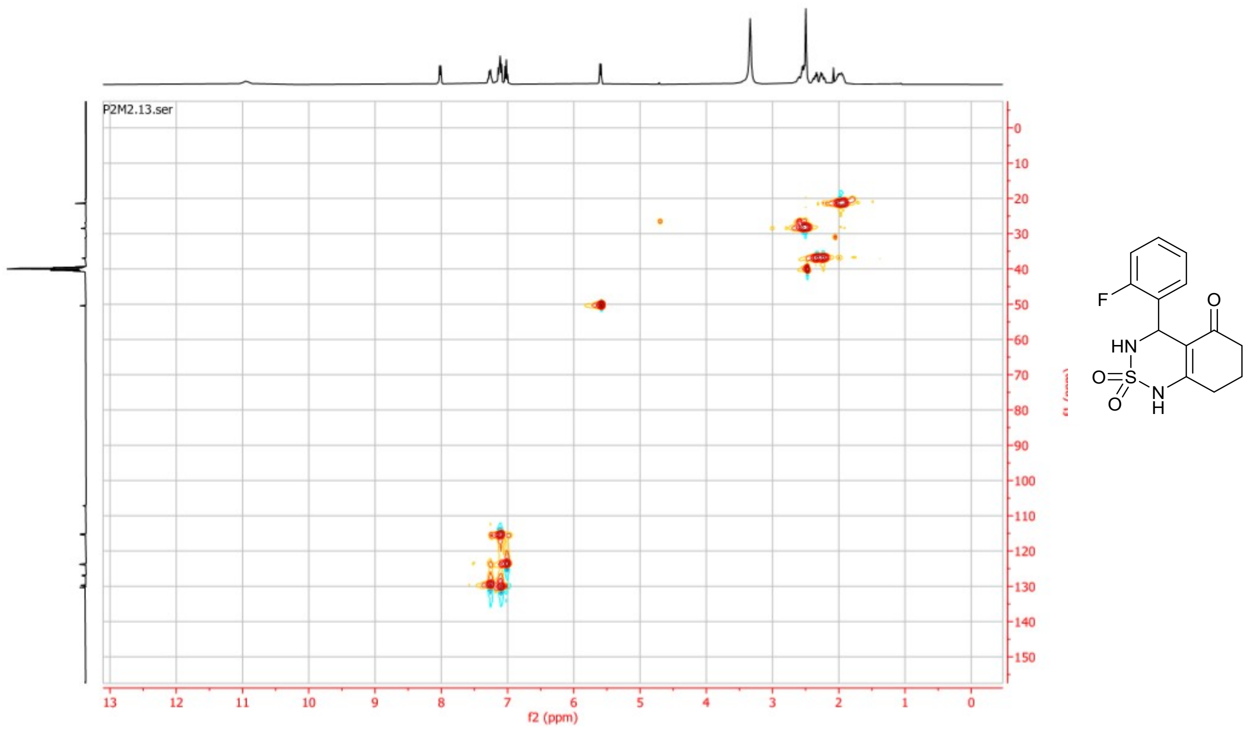

HMQC Spectrum: 4-(2-fluorophenyl)-4,6,7,8-tetrahydro-1H-benzo[c][1,2,6]thiadiazin-5(3H)-one 2,2-dioxide

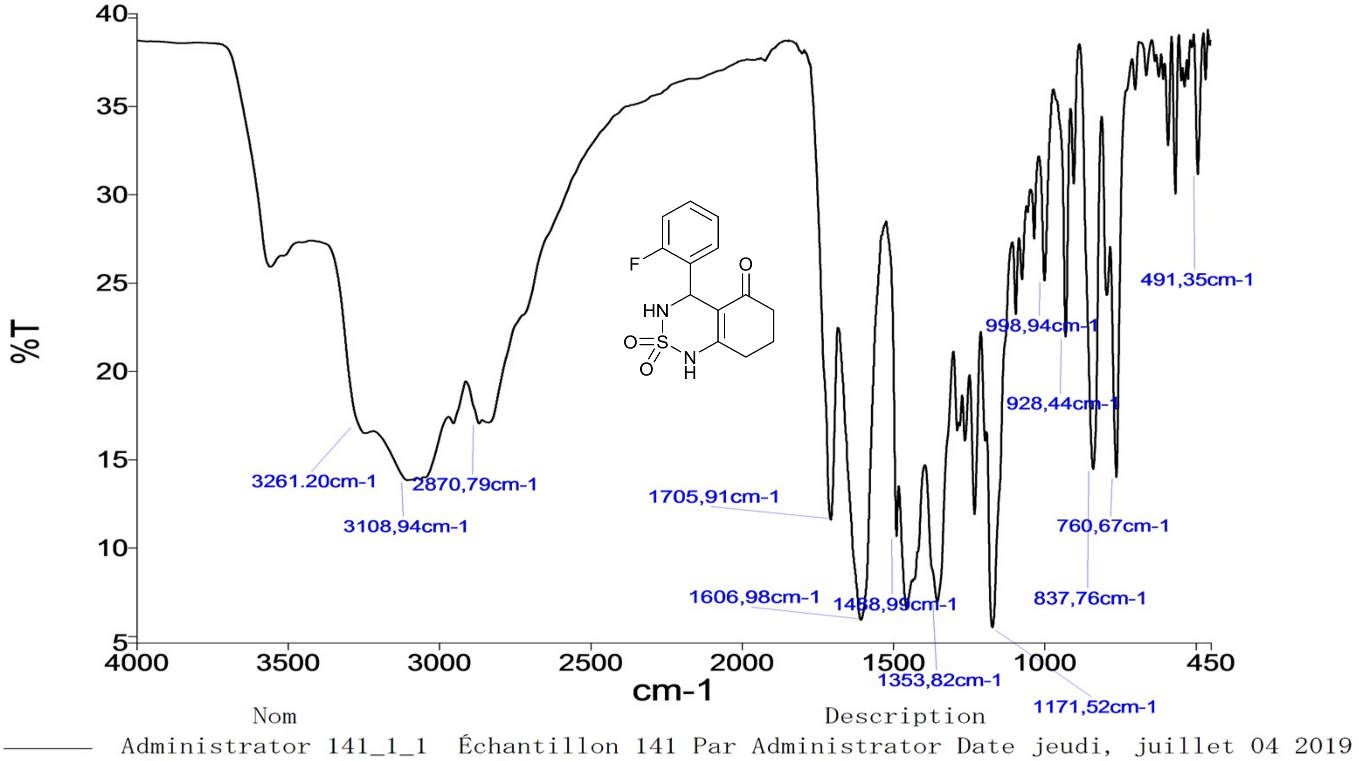

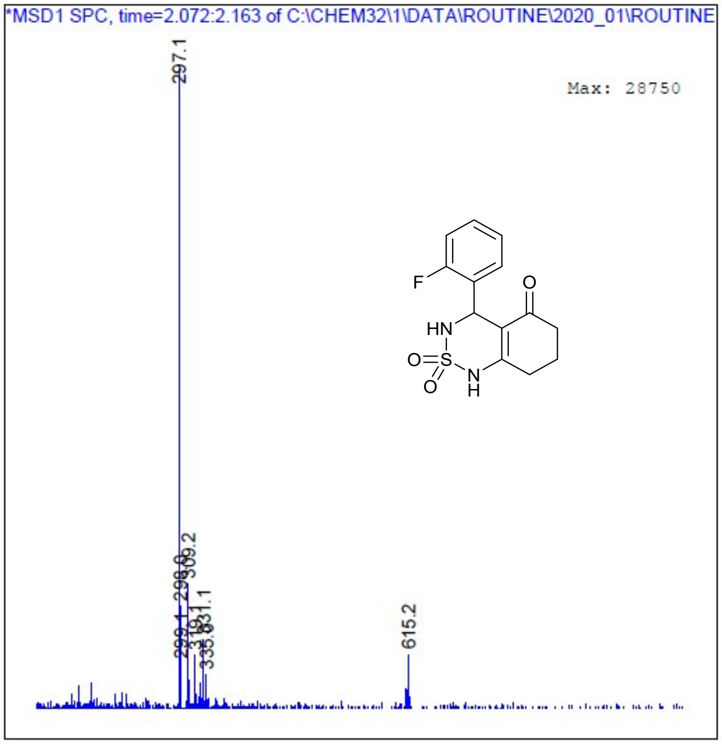

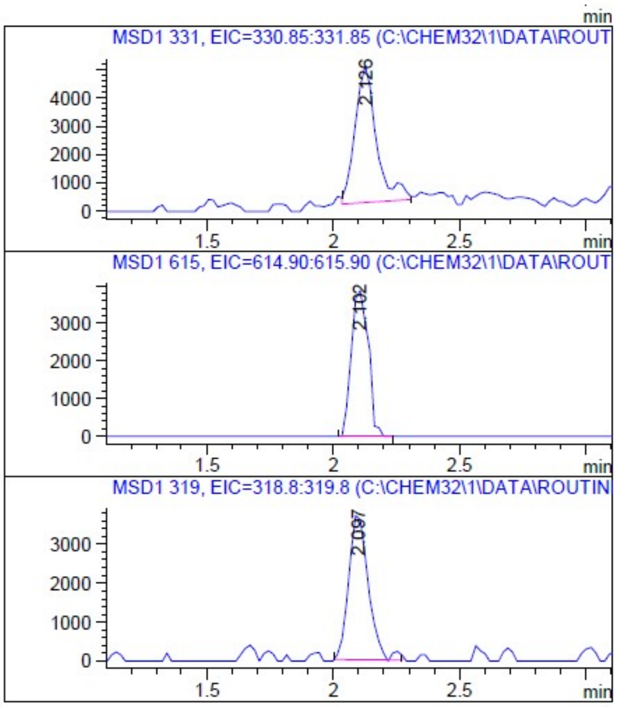

IR Spectrum: 4-(2-fluorophenyl)-4,6,7,8-tetrahydro-1H-benzo[c][1,2,6]thiadiazin-5(3H)-one 2,2-dioxide

LC-MS: 4-(2-fluorophenyl)-4,6,7,8-tetrahydro-1H-benzo[c][1,2,6]thiadiazin-5(3H)-one 2,2-dioxide

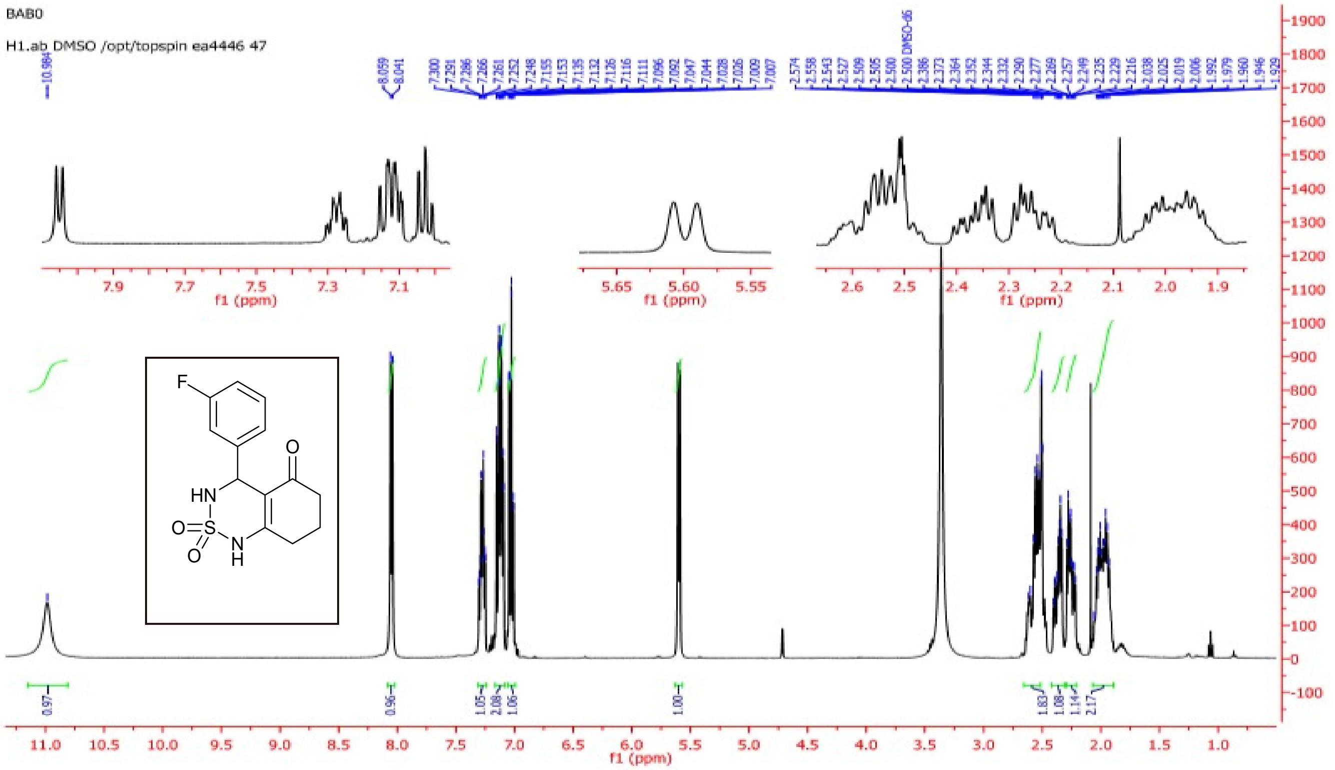

^{1}H NMR Spectrum: 4-(3-fluorophenyl)-4,6,7,8-tetrahydro-1H-benzo[c][1,2,6]thiadiazin-5(3H)-one 2,2-dioxide

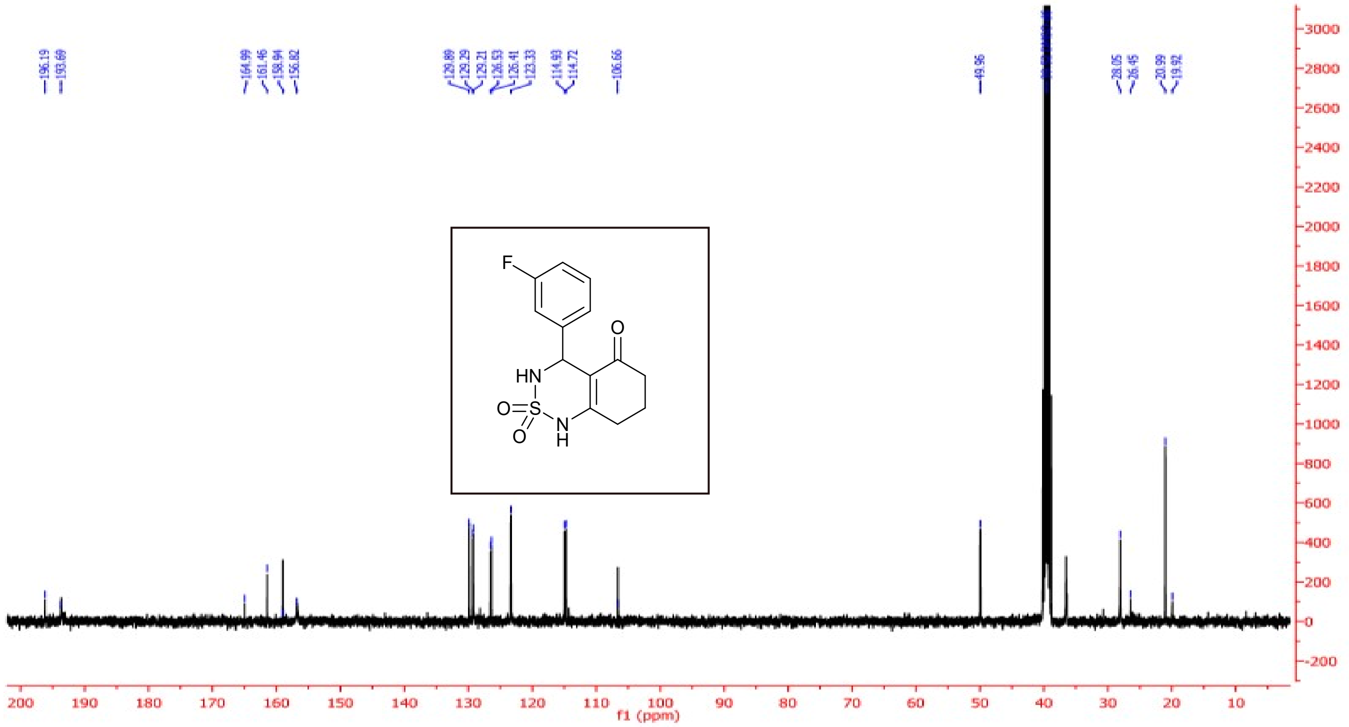

^{13}C NMR Spectrum: 4-(3-fluorophenyl)-4,6,7,8-tetrahydro-1H-benzo[c][1,2,6]thiadiazin-5(3H)-one 2,2-dioxide

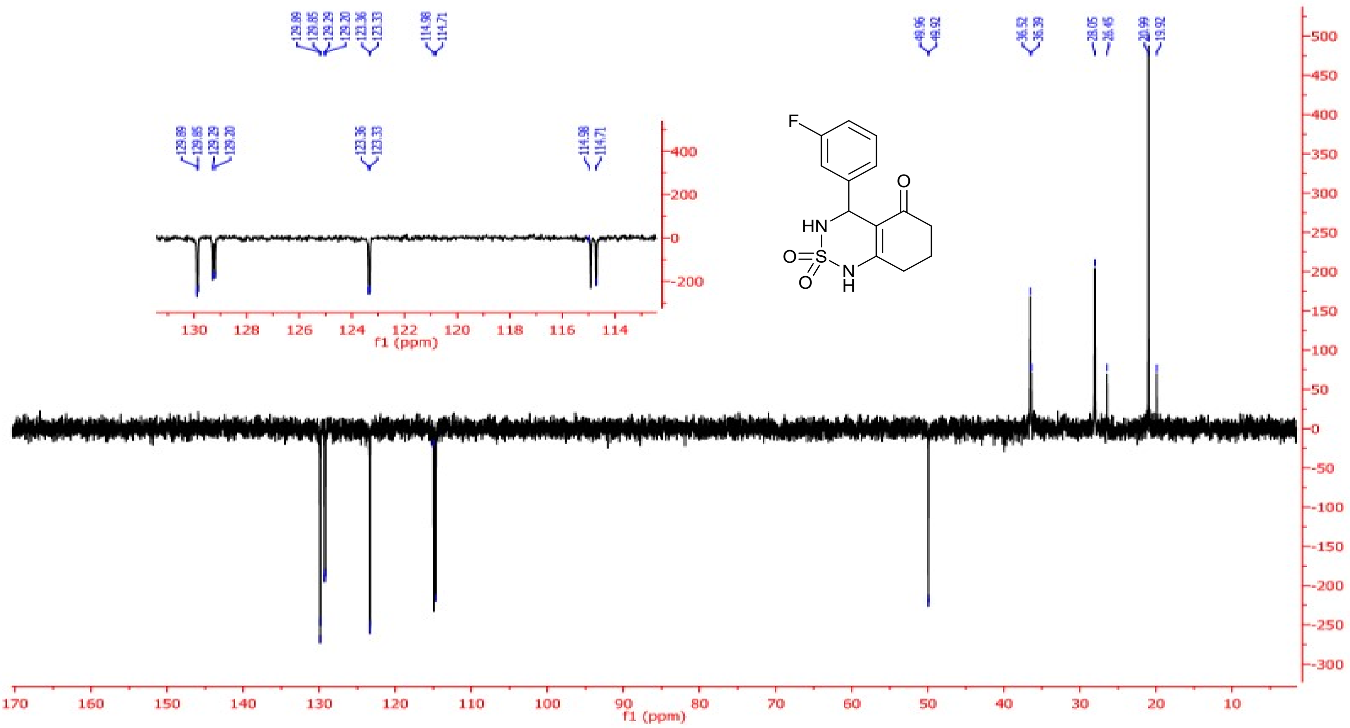

DEPT NMR Spectrum: 4-(3-fluorophenyl)-4,6,7,8-tetrahydro-1H-benzo[c][1,2,6]thiadiazin-5(3H)-one 2,2-

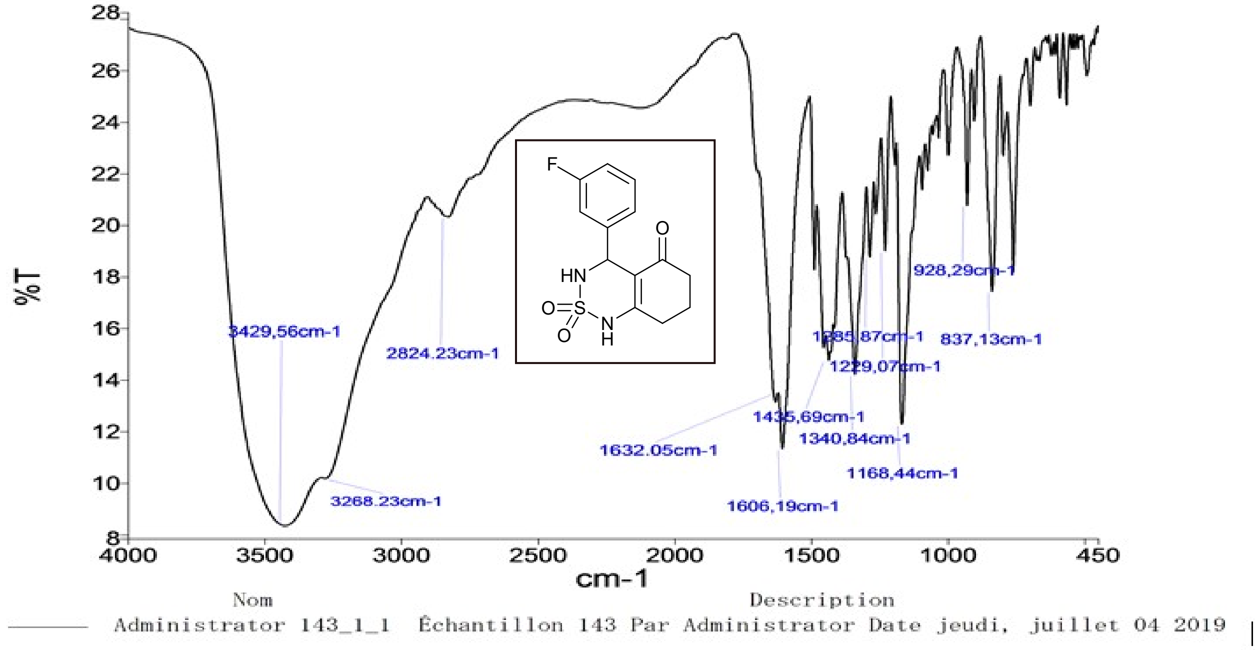

dioxide

IR Spectrum: 4-(3-fluorophenyl)-4,6,7,8-tetrahydro-1H-benzo[c][1,2,6]thiadiazin-5(3H)-one 2,2-dioxide

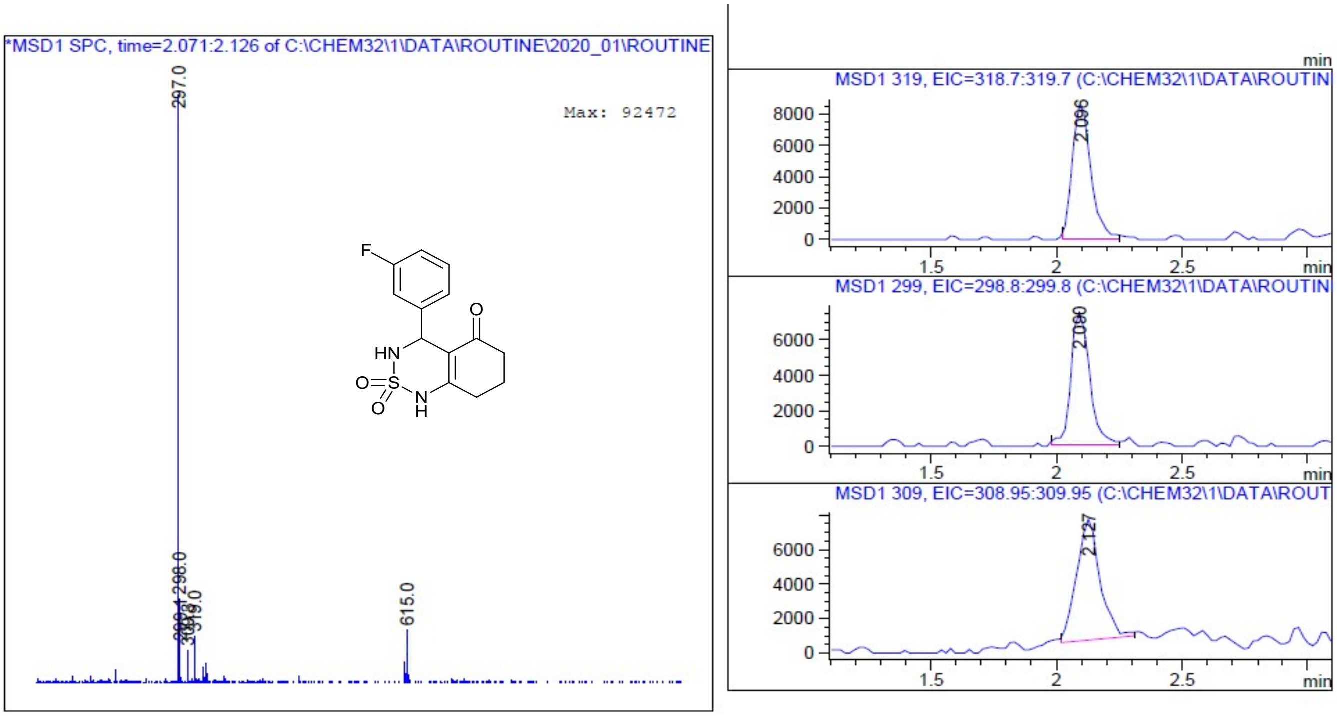

LC-MS: 4-(3-fluorophenyl)-4,6,7,8-tetrahydro-1H-benzo[c][1,2,6]thiadiazin-5(3H)-one 2,2-dioxide

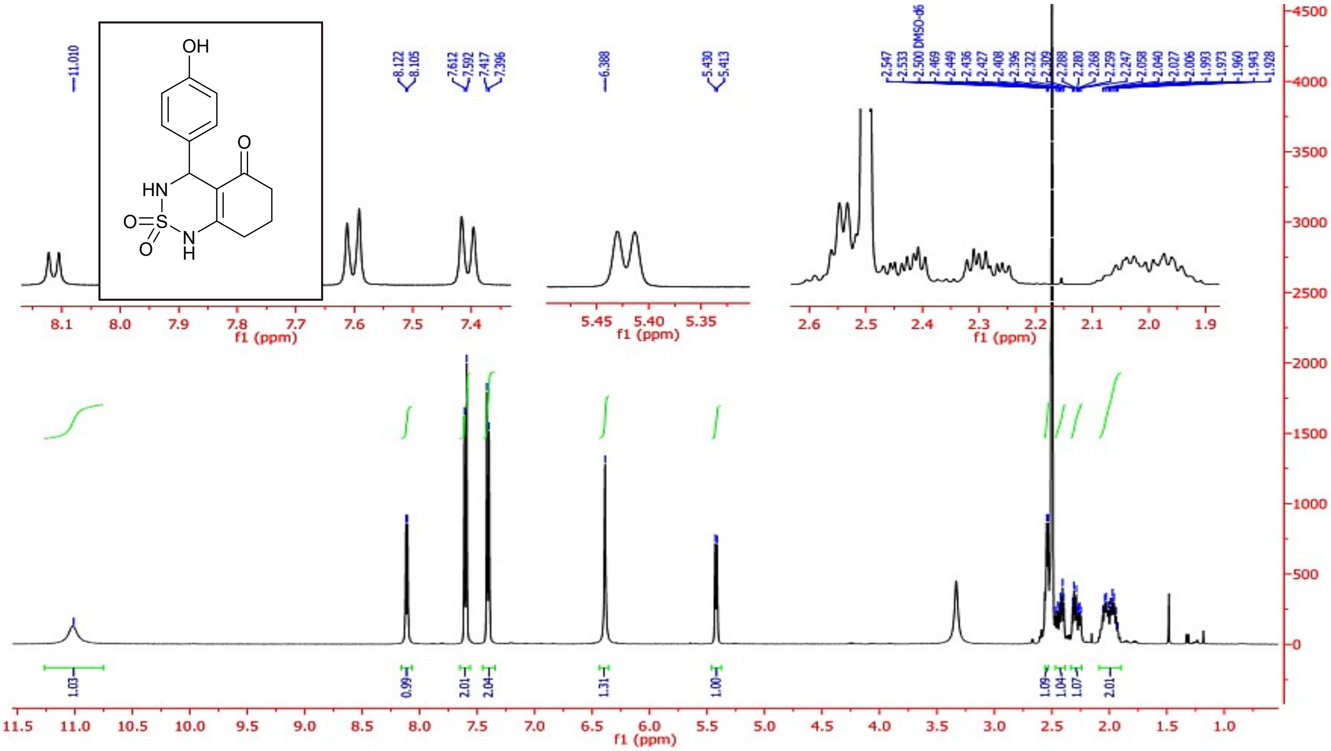

^{1}H NMR Spectrum: 4-(4-hydroxyphenyl)-4,6,7,8-tetrahydro-1H-benzo[c][1,2,6]thiadiazin-5(3H)-one 2,2-dioxide

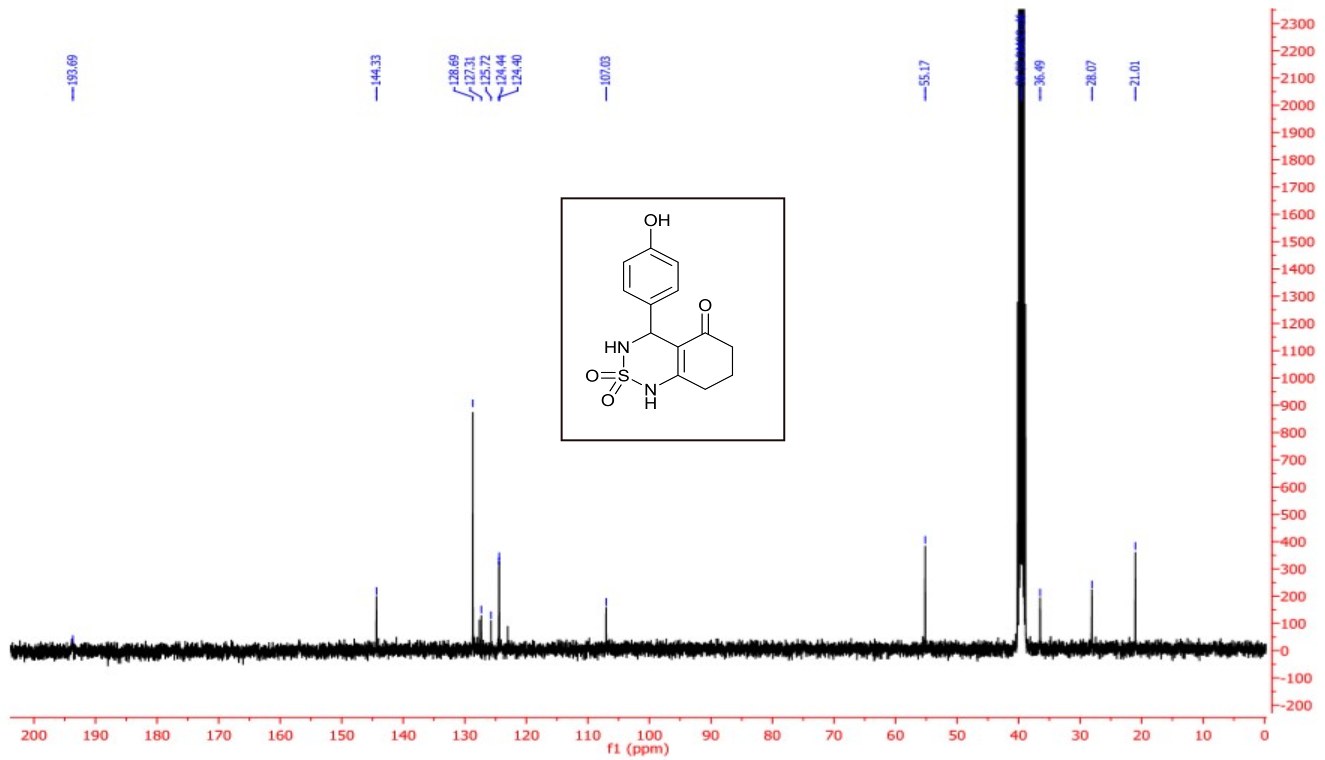

¹³C NMR Spectrum: 4-(4-hydroxyphenyl)-4,6,7,8-tetrahydro-1H-benzo[c][1,2,6]thiadiazin-5(3H)-one 2,2-dioxide

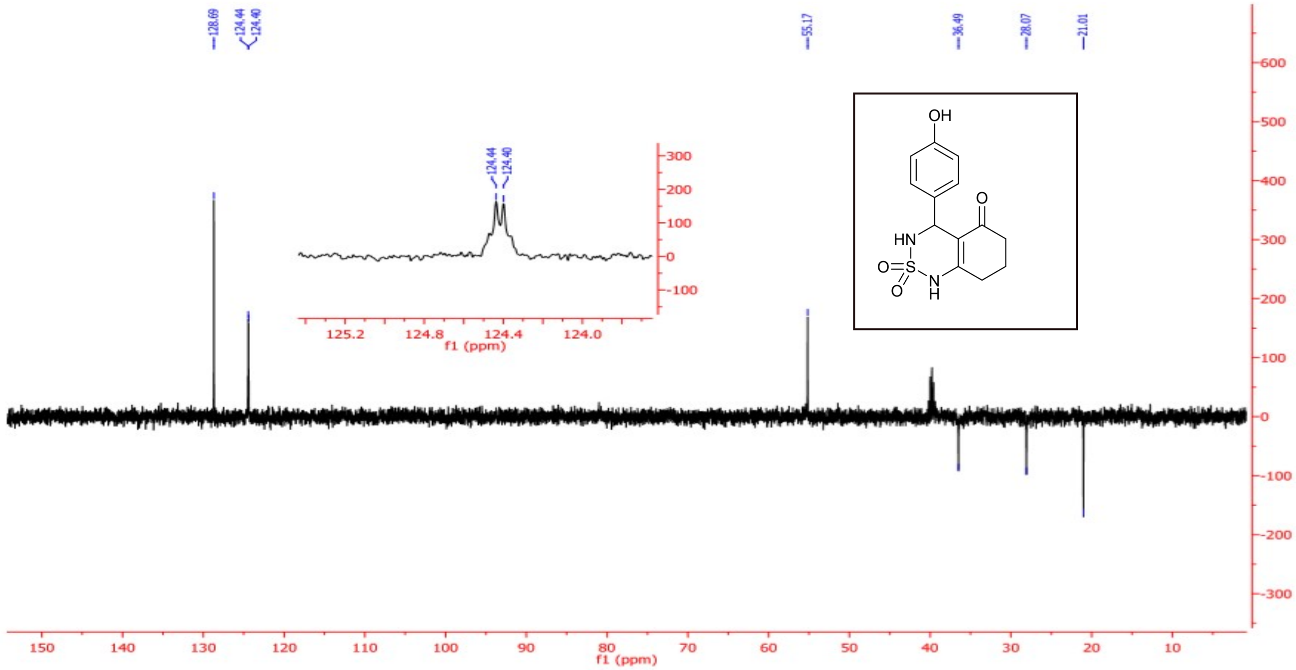

DEPT NMR Spectrum: 4-(4-hydroxyphenyl)-4,6,7,8-tetrahydro-1H-benzo[c][1,2,6]thiadiazin-5(3H)-one 2,2-dioxide

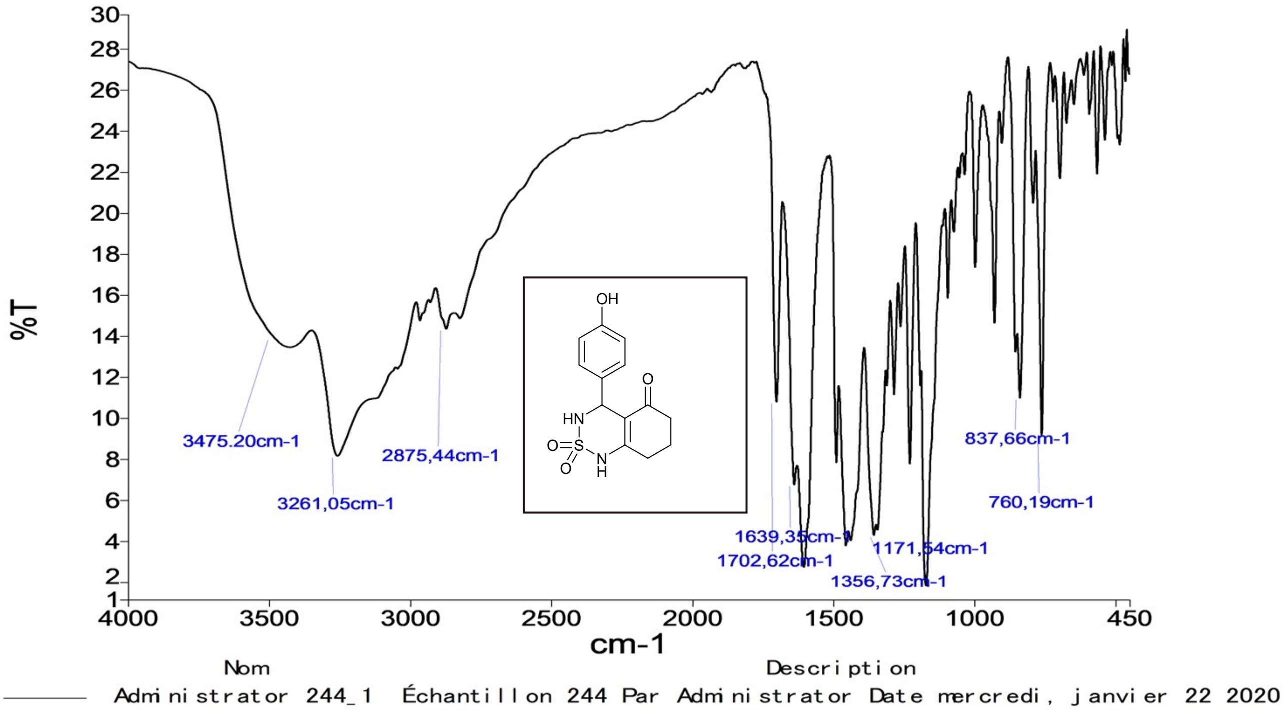

IR Spectrum: 4-(4-hydroxyphenyl)-4,6,7,8-tetrahydro-1H-benzo[c][1,2,6]thiadiazin-5(3H)-one 2,2-dioxide

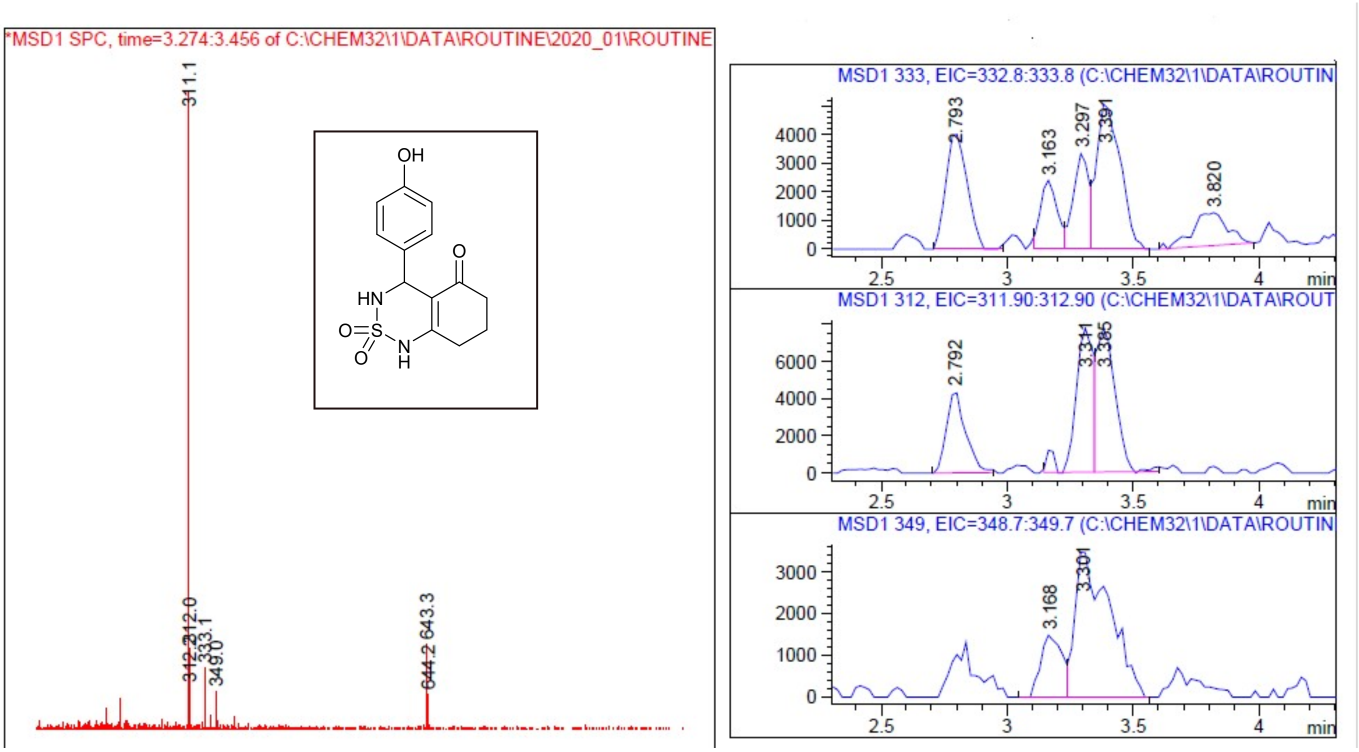

LC-MS: 4-(4-hydroxyphenyl)-4,6,7,8-tetrahydro-1H-benzo[c][1,2,6]thiadiazin-5(3H)-one 2,2-dioxide

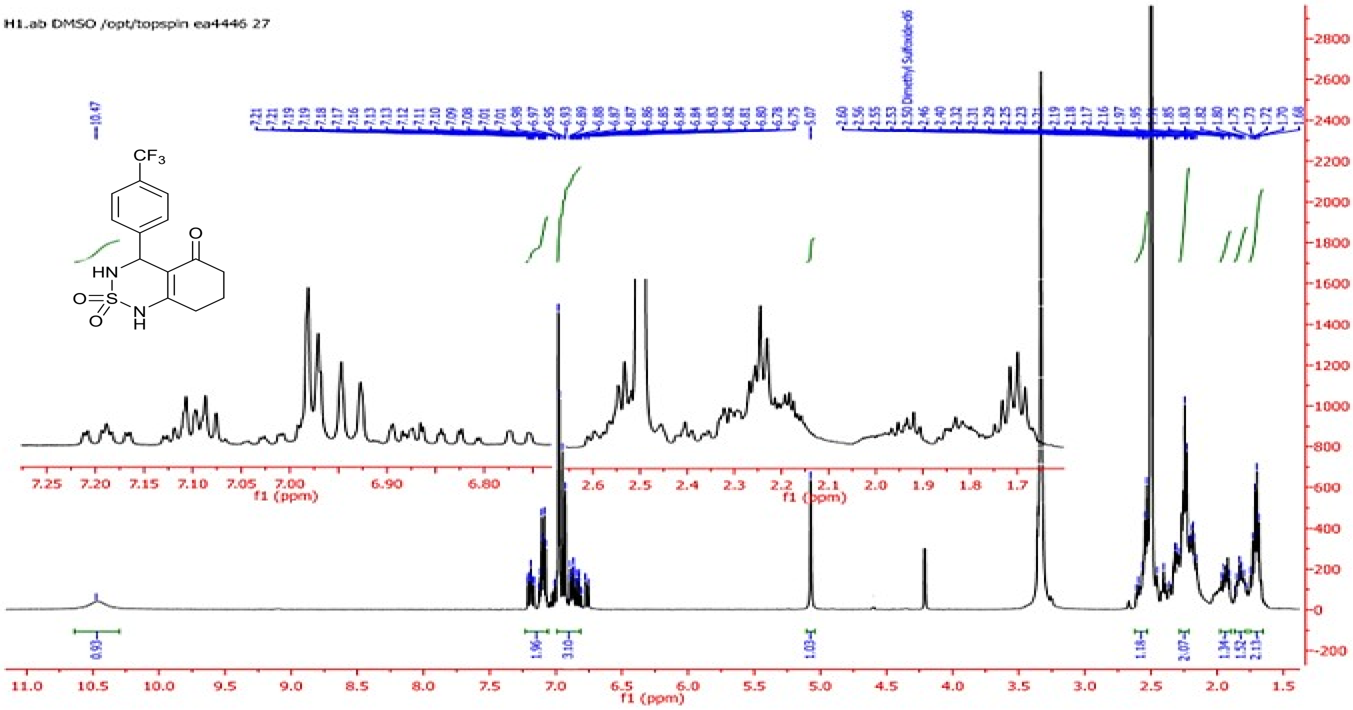

^{1}H NMR Spectrum: 4-(4-(trifluoromethyl)phenyl)-4,6,7,8-tetrahydro-1H-benzo[c][1,2,6]thiadiazin-5(3H)-one 2,2-dioxide

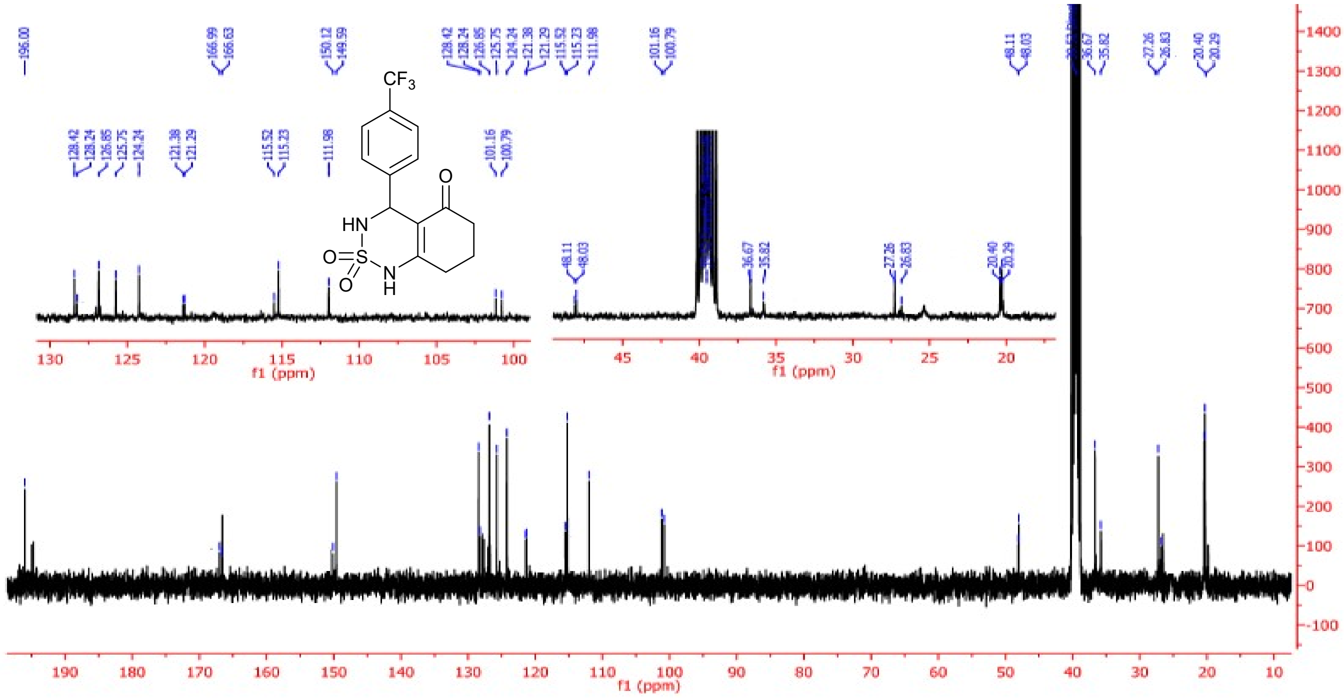

^{13}C NMR Spectrum: 4-(4-(trifluoromethyl)phenyl)-4,6,7,8-tetrahydro-1H-benzo[c][1,2,6]thiadiazin-5(3H)-one 2,2-dioxide

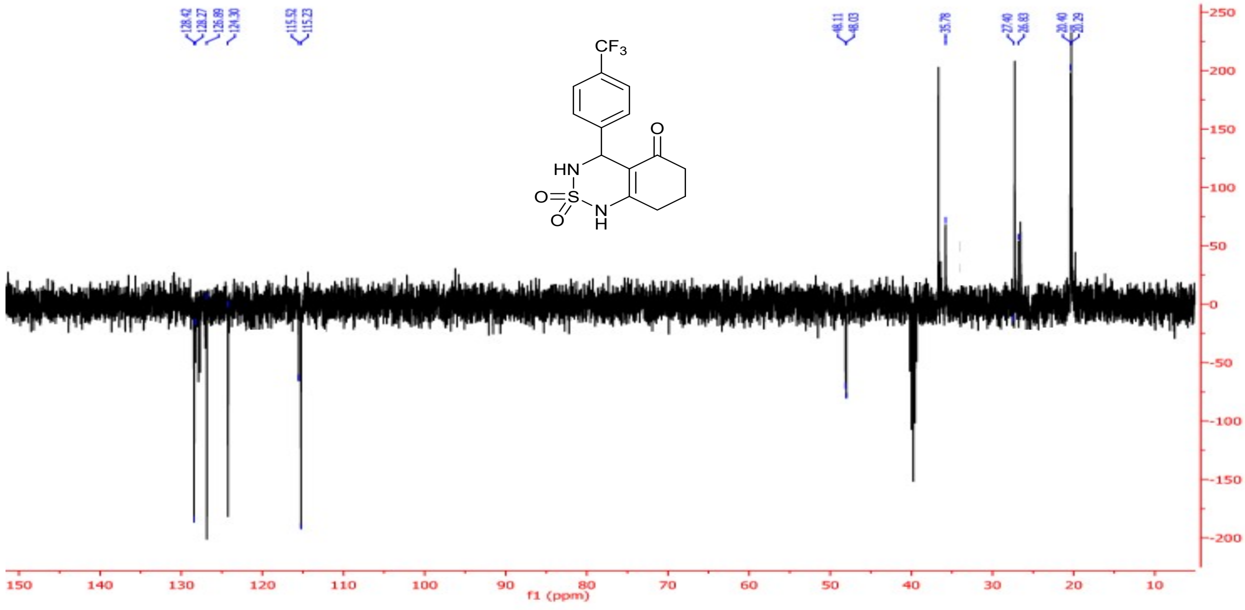

DEPT NMR Spectrum: 4-(4-(trifluoromethyl)phenyl)-4,6,7,8-tetrahydro-1H-benzo[c][1,2,6]thiadiazin-

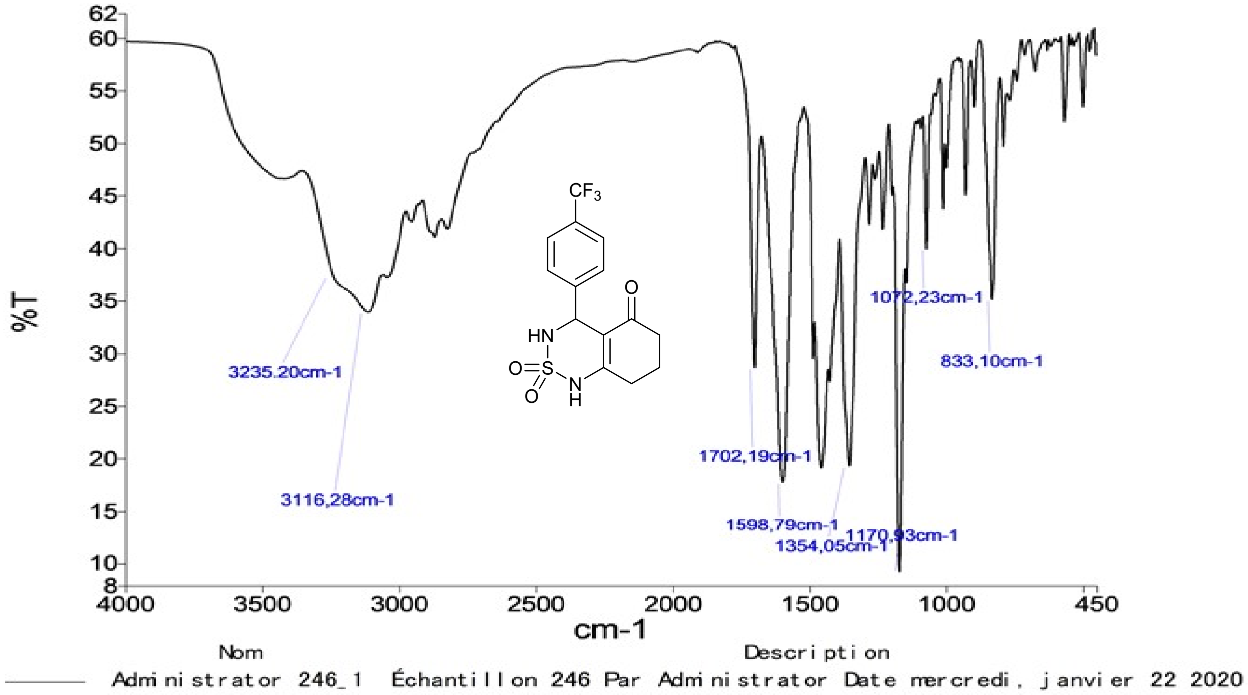

5(3H)-one 2,2-dioxide

IR Spectrum: 4-(4-(trifluoromethyl)phenyl)-4,6,7,8-tetrahydro-1H-benzo[c][1,2,6]thiadiazin-5(3H)-one 2,2-dioxide

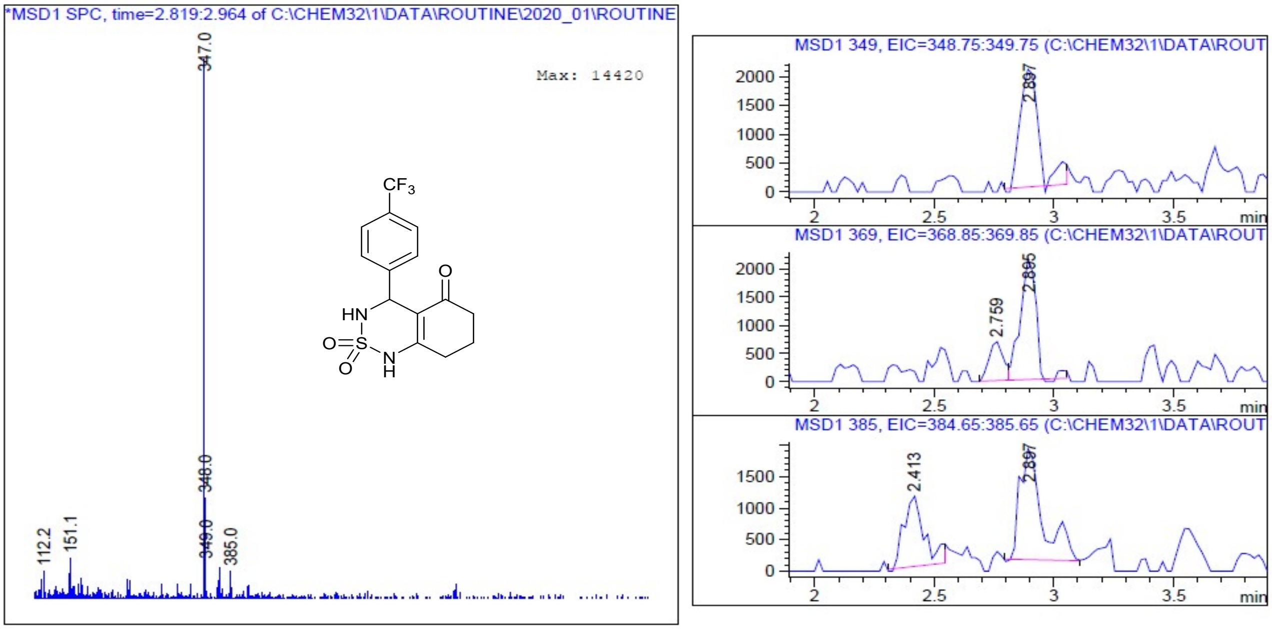

LC-MS: 4-(4-(trifluoromethyl)phenyl)-4,6,7,8-tetrahydro-1H-benzo[c][1,2,6]thiadiazin-5(3H)-one 2,2-dioxide

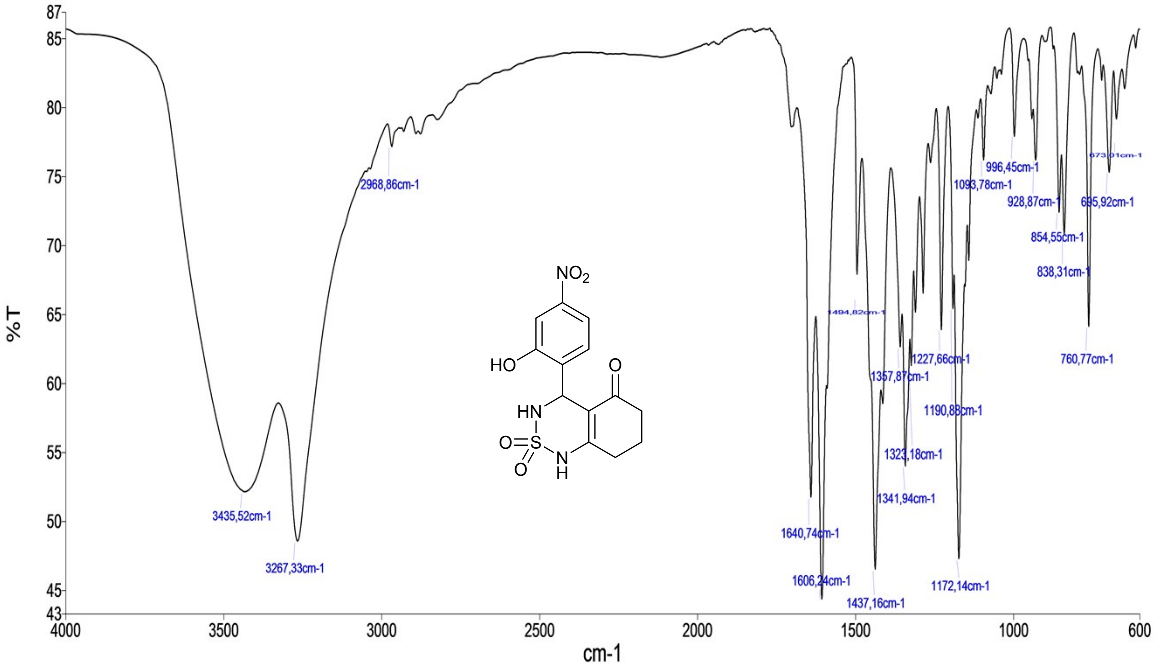

IR Spectrum: 4-(4-bromophenyl)-4,6,7,8-tetrahydro-1H-benzo[c][1,2,6]thiadiazin-5(3H)-one 2,2-dioxide

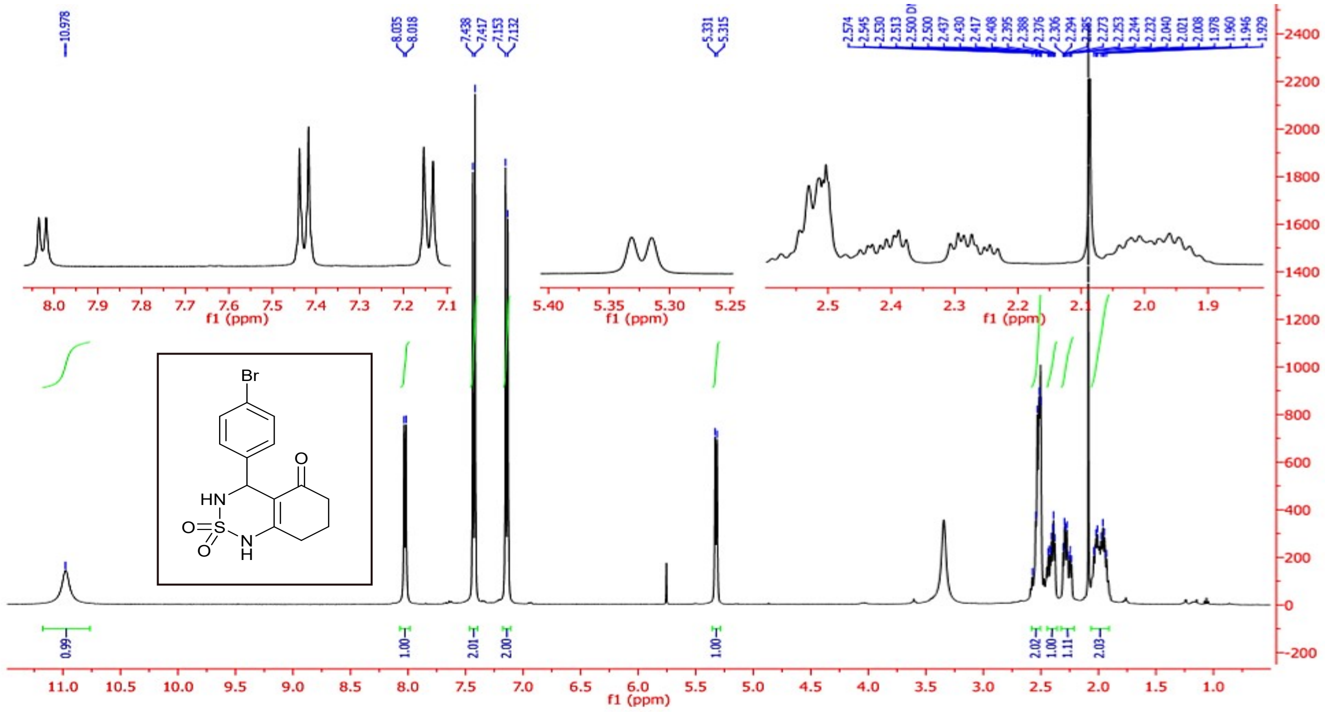

¹H NMR Spectrum: 4-(4-bromophenyl)-4,6,7,8-tetrahydro-1H-benzo[c][1,2,6]thiadiazin-5(3H)-one 2,2-dioxide

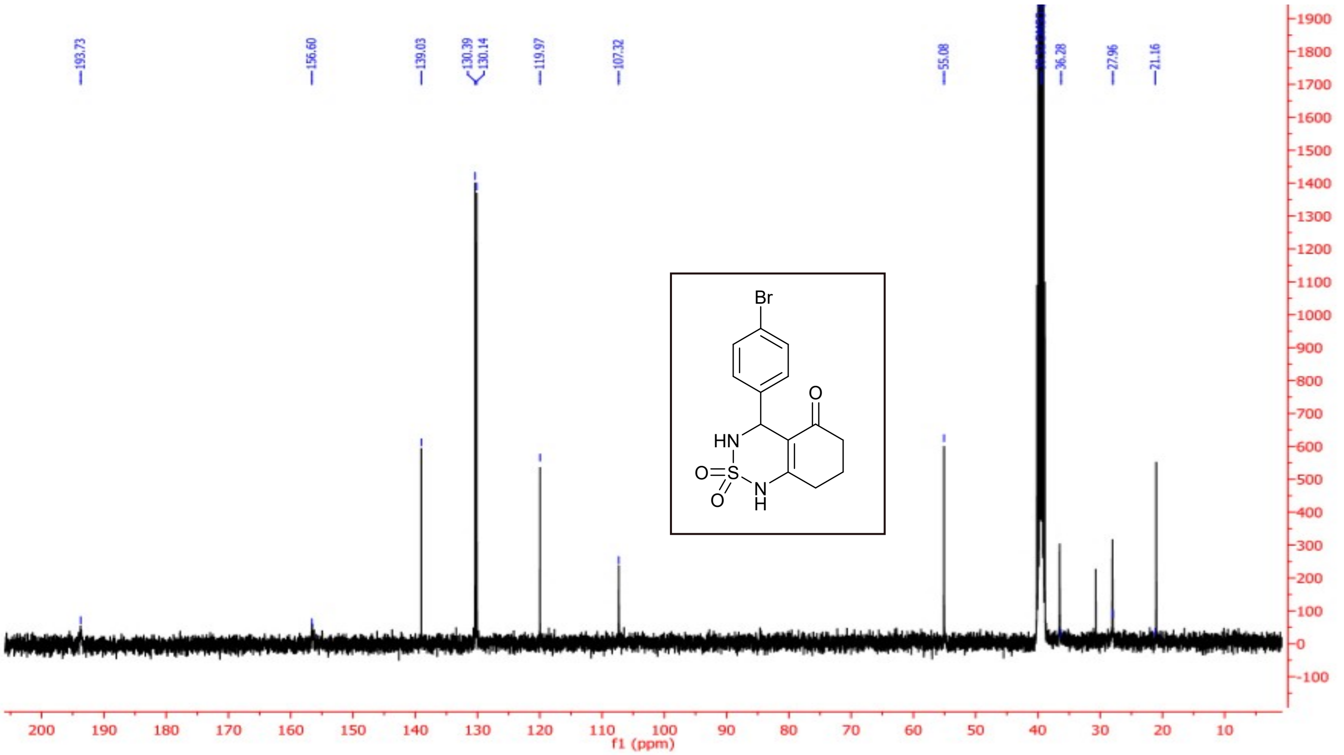

¹³C NMR Spectrum: 4-(4-bromophenyl)-4,6,7,8-tetrahydro-1H-benzo[c][1,2,6]thiadiazin-5(3H)-one 2,2-dioxide

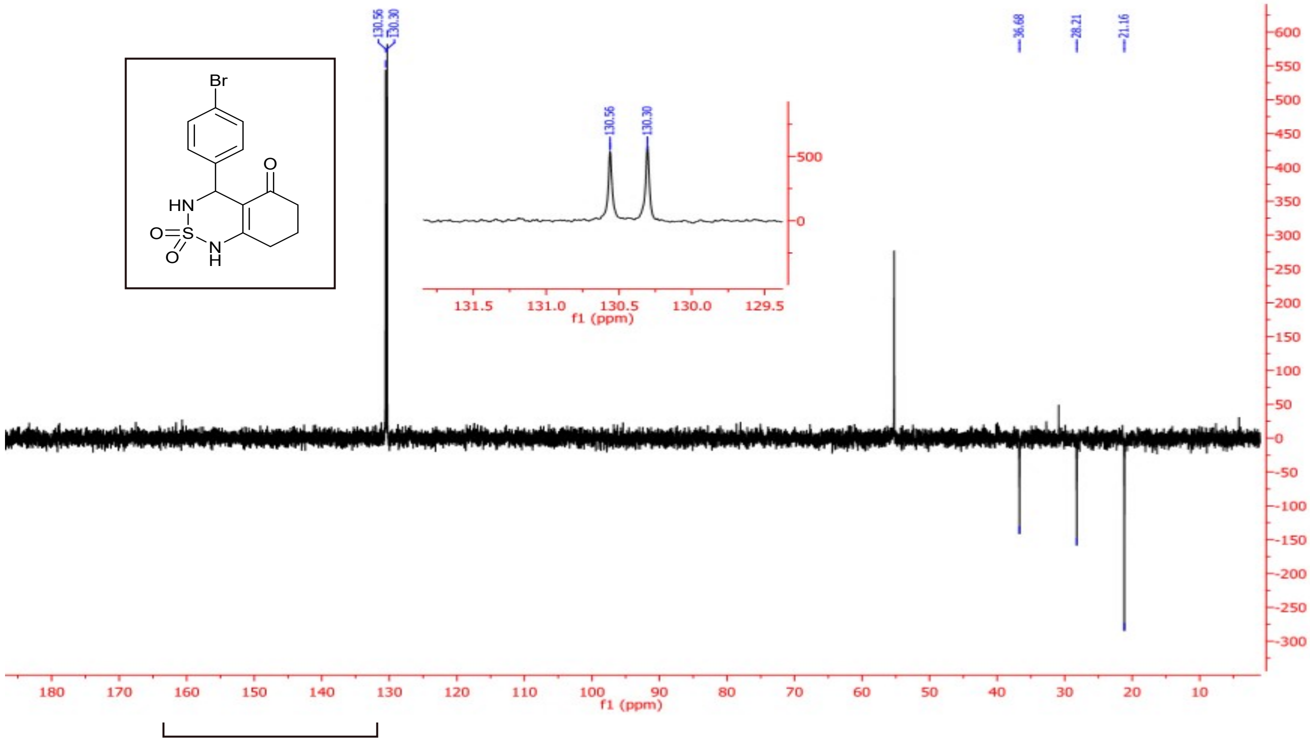

DEPT NMR Spectrum: 4-(4-bromophenyl)-4,6,7,8-tetrahydro-1H-benzo[c][1,2,6]thiadiazin-5(3H)-one 2,2-dioxide

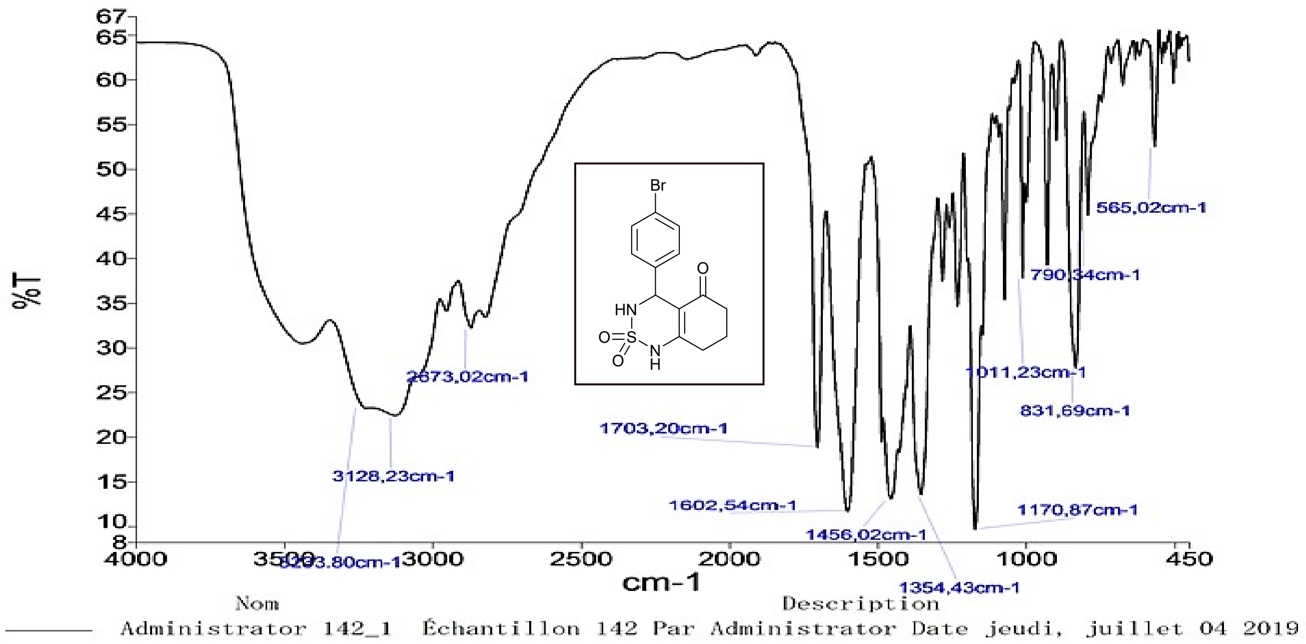

IR Spectrum: 4-(4-bromophenyl)-4,6,7,8-tetrahydro-1H-benzo[c][1,2,6]thiadiazin-5(3H)-one 2,2-dioxide

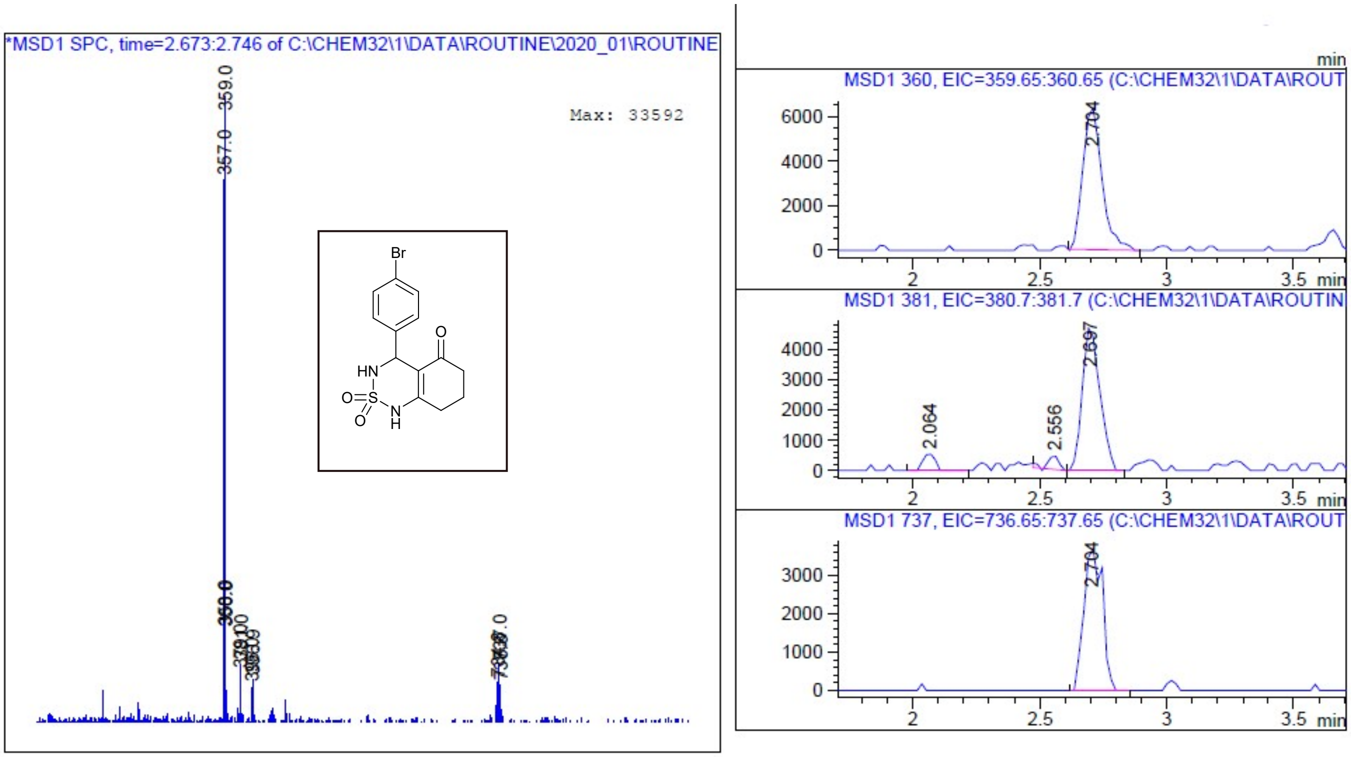

LC-MS: 4-(4-bromophenyl)-4,6,7,8-tetrahydro-1H-benzo[c][1,2,6]thiadiazin-5(3H)-one 2,2 dioxide

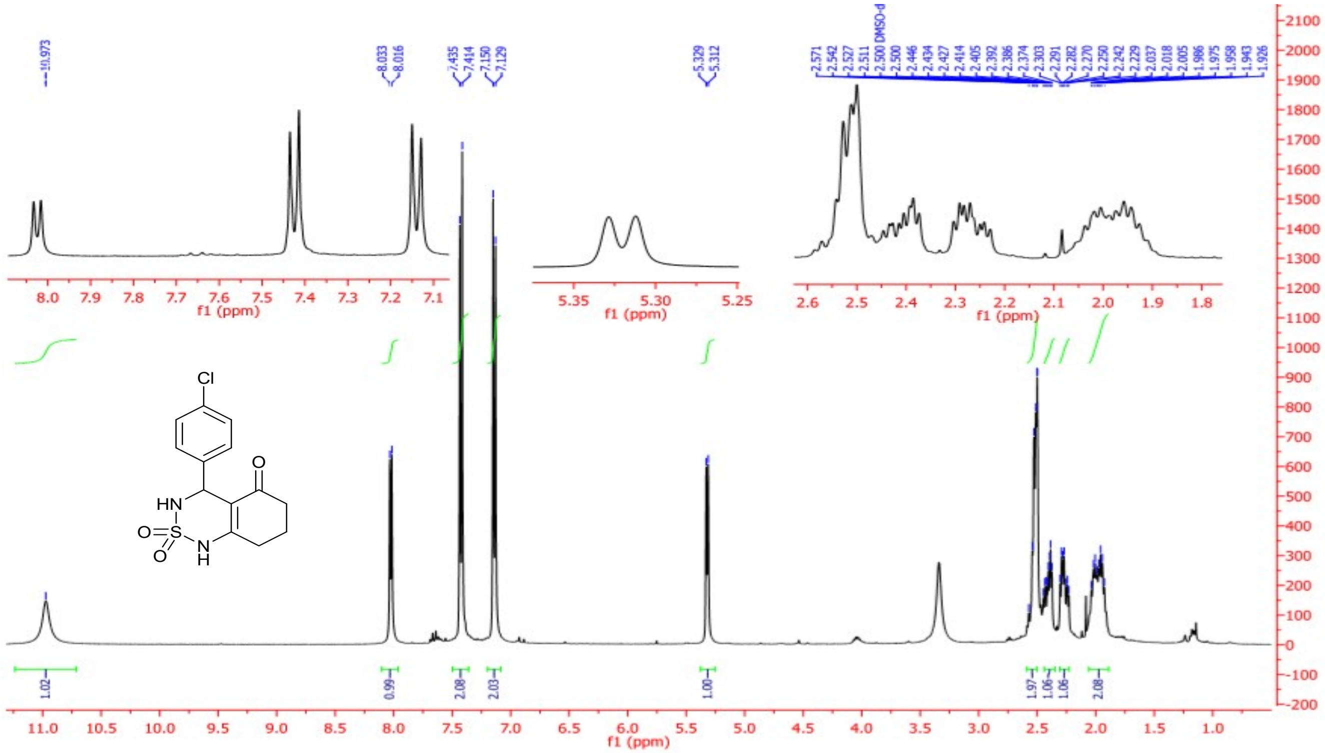

¹H NMR Spectrum: 4-(4-chlorophenyl)-4,6,7,8-tetrahydro-1H-benzo[c][1,2,6]thiadiazin-5(3H)-one 2,2-

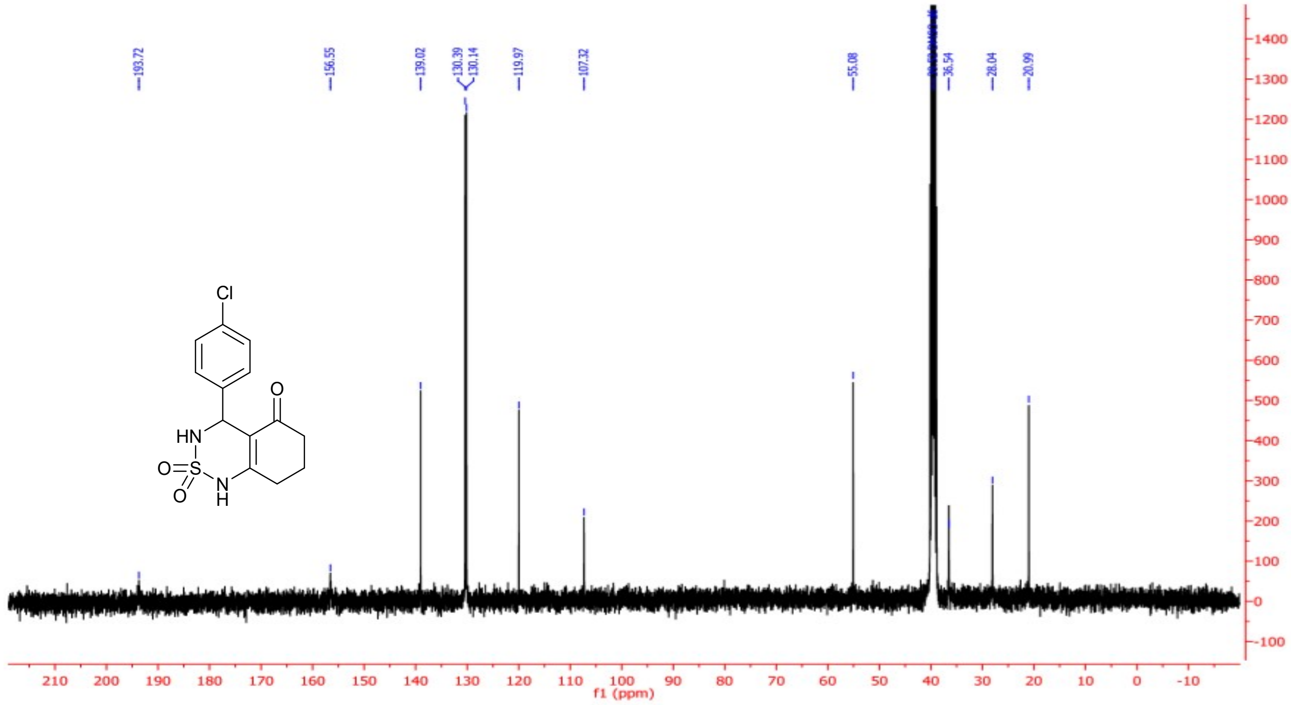

dioxide

¹³C NMR Spectrum: 4-(4-chlorophenyl)-4,6,7,8-tetrahydro-1H-benzo[c][1,2,6]thiadiazin-5(3H)-one 2,2-dioxide

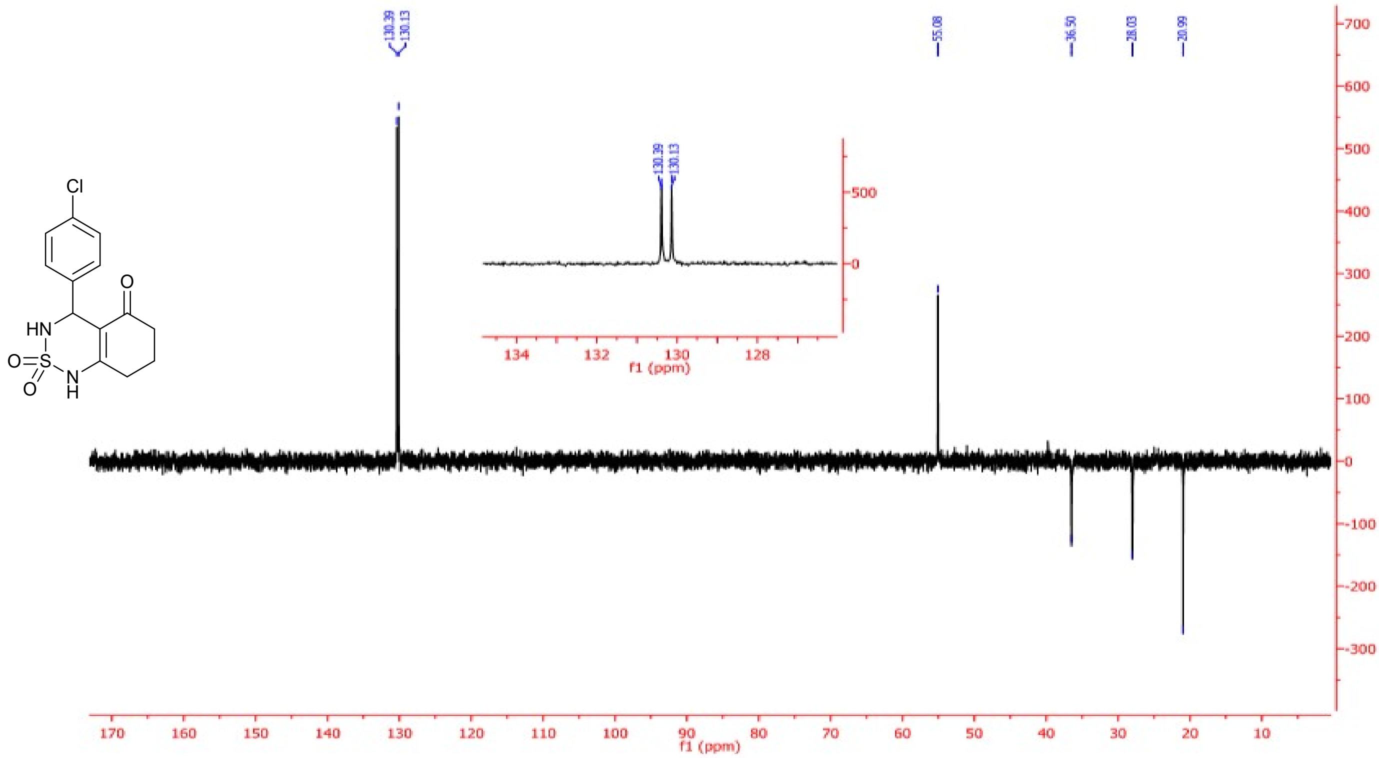

DEPT NMR Spectrum: 4-(4-chlorophenyl)-4,6,7,8-tetrahydro-1H-benzo[c][1,2,6]thiadiazin-5(3H)-one 2,2-dioxide

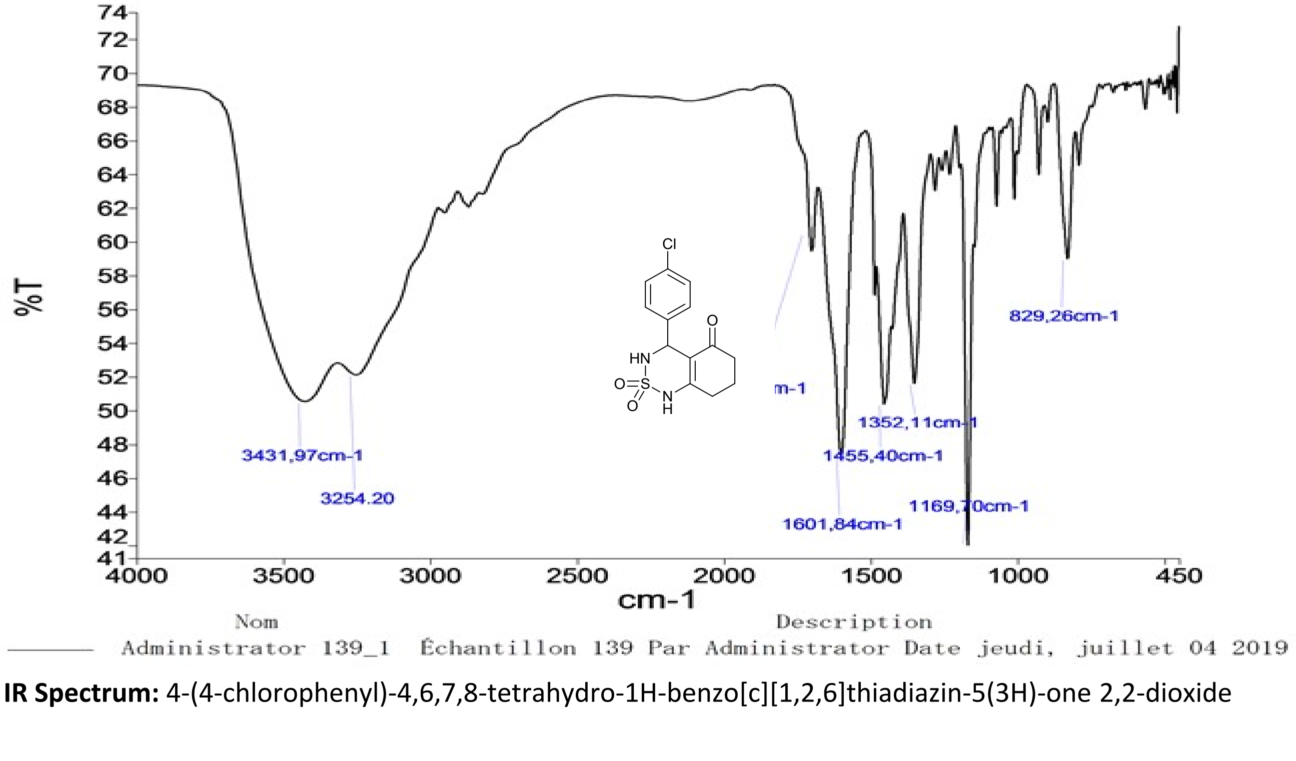

IR Spectrum: 4-(4-chlorophenyl)-4,6,7,8-tetrahydro-1H-benzo[c][1,2,6]thiadiazin-5(3H)-one 2,2-dioxide

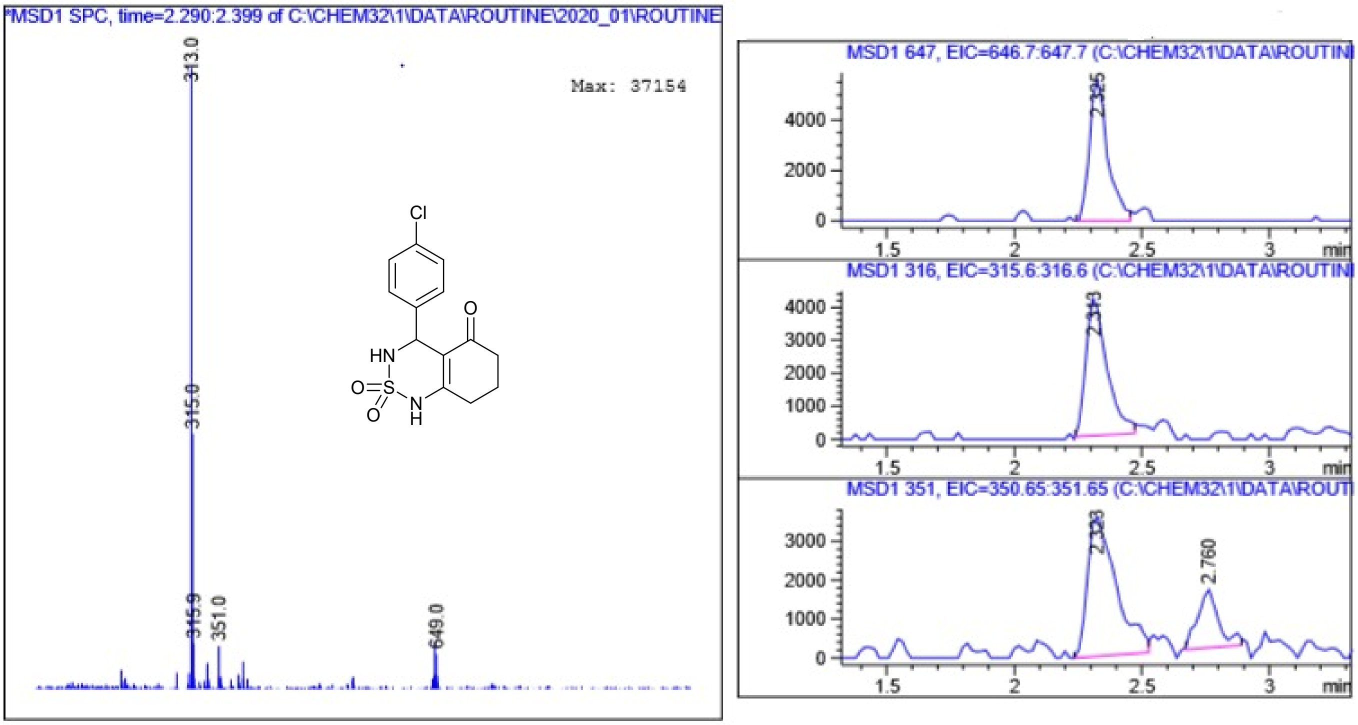

LC-MS: 4-(4-chlorophenyl)-4,6,7,8-tetrahydro-1H-benzo[c][1,2,6]thiadiazin-5(3H)-one 2,2-dioxide

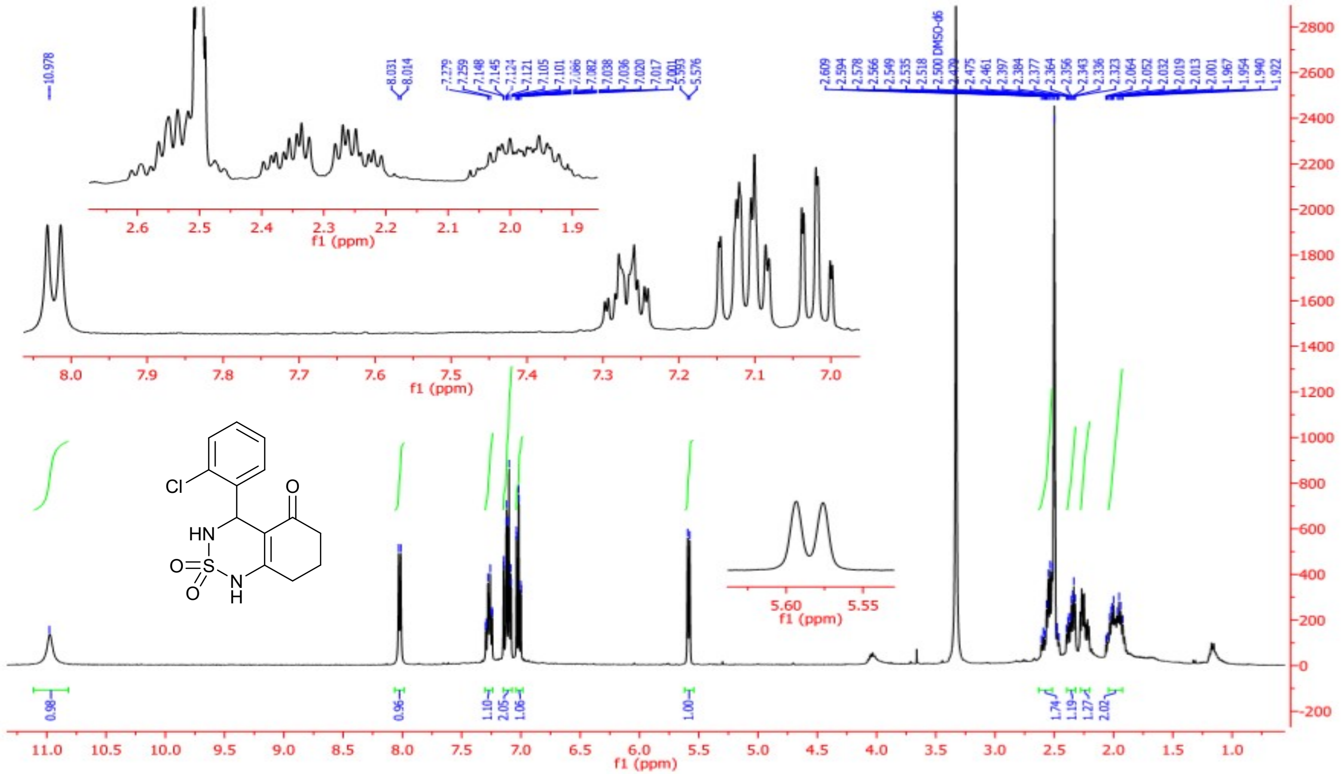

¹H NMR Spectrum: 4-(4-bromophenyl)-4,6,7,8-tetrahydro-1H-benzo[c][1,2,6]thiadiazin-5(3H)-one 2,2-dioxide

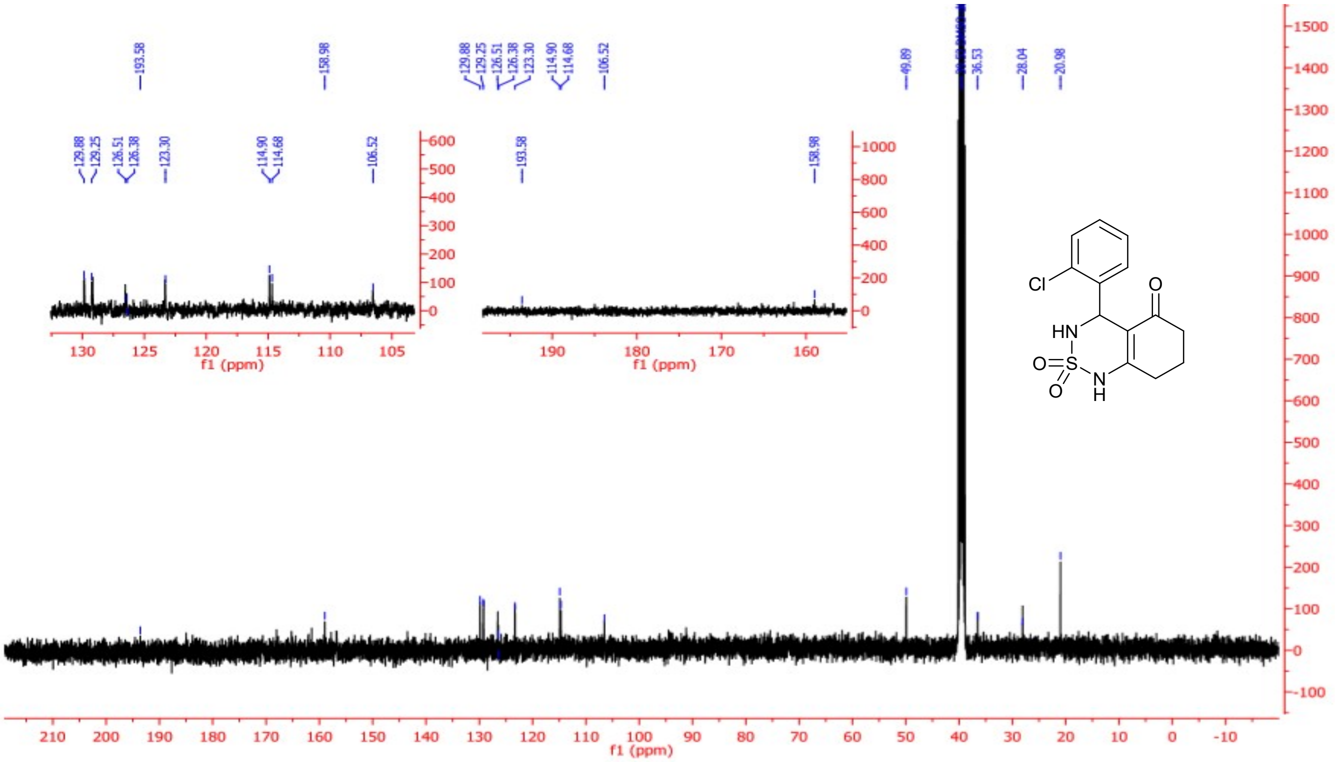

^{13}C NMR Spectrum: 4-(4-bromophenyl)-4,6,7,8-tetrahydro-1H-benzo[c][1,2,6]thiadiazin-5(3H)-one 2,2-dioxide

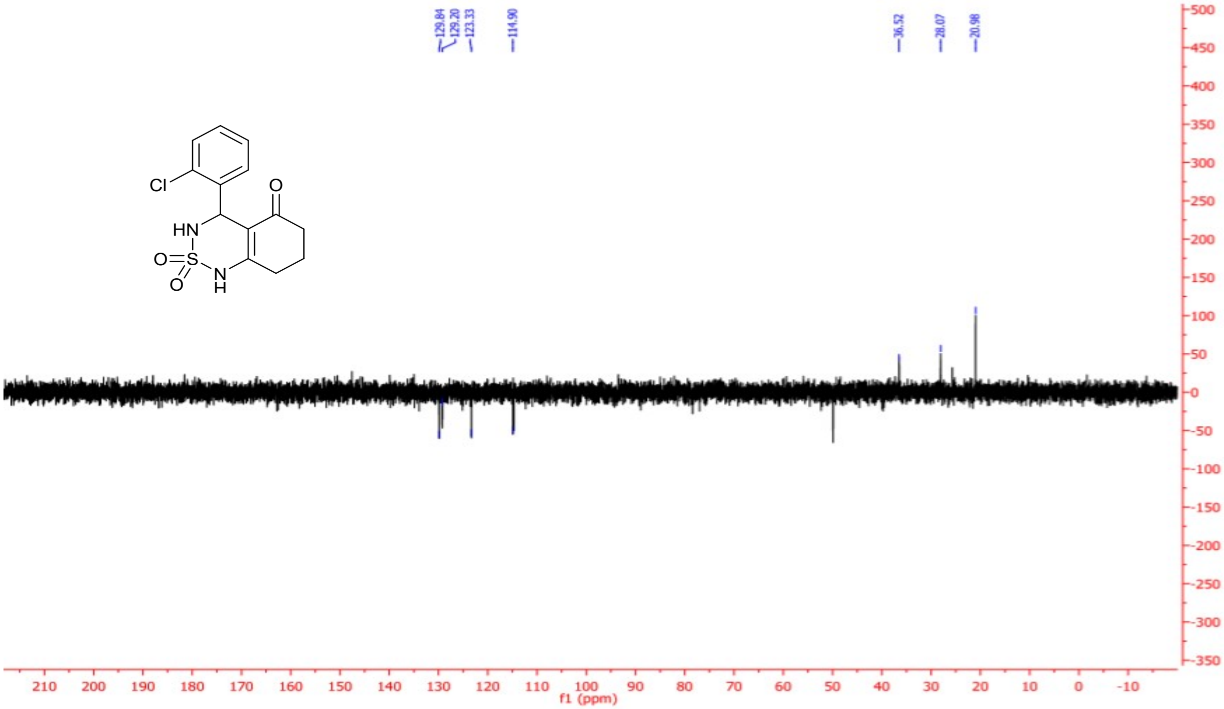

DEPT NMR Spectrum: 4-(4-bromophenyl)-4,6,7,8-tetrahydro-1H-benzo[c][1,2,6]thiadiazin-5(3H)-one 2,2-dioxide

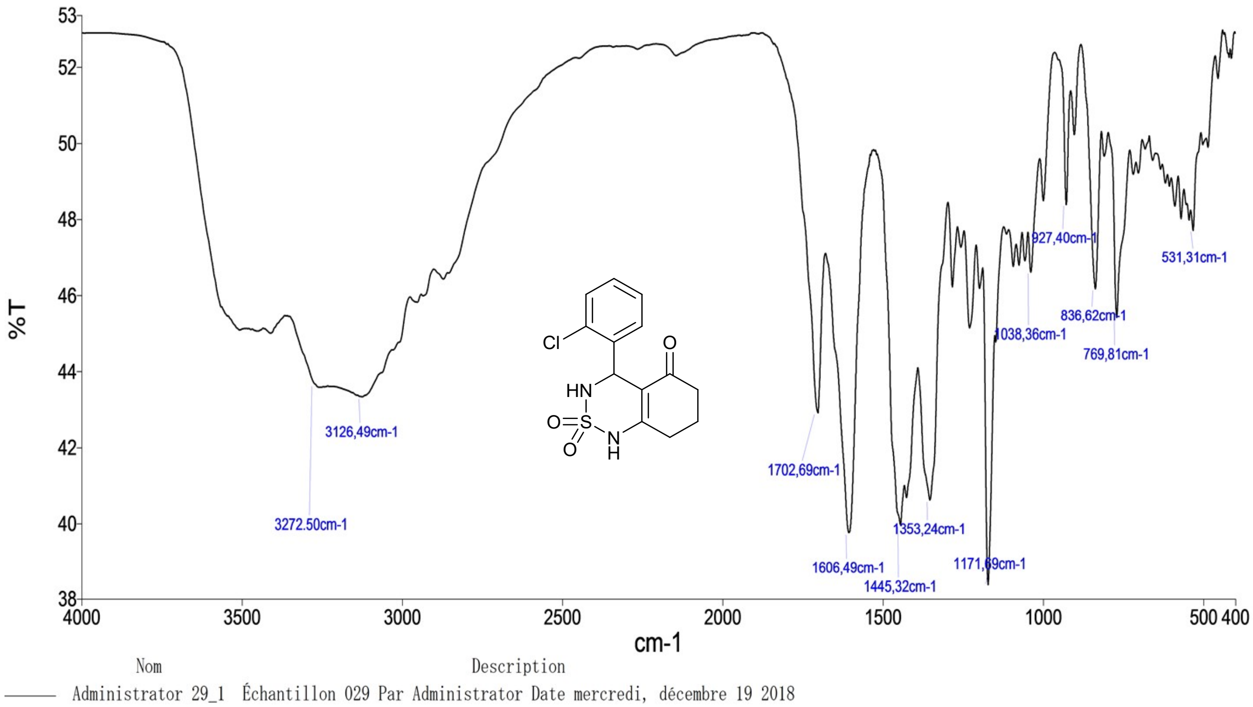

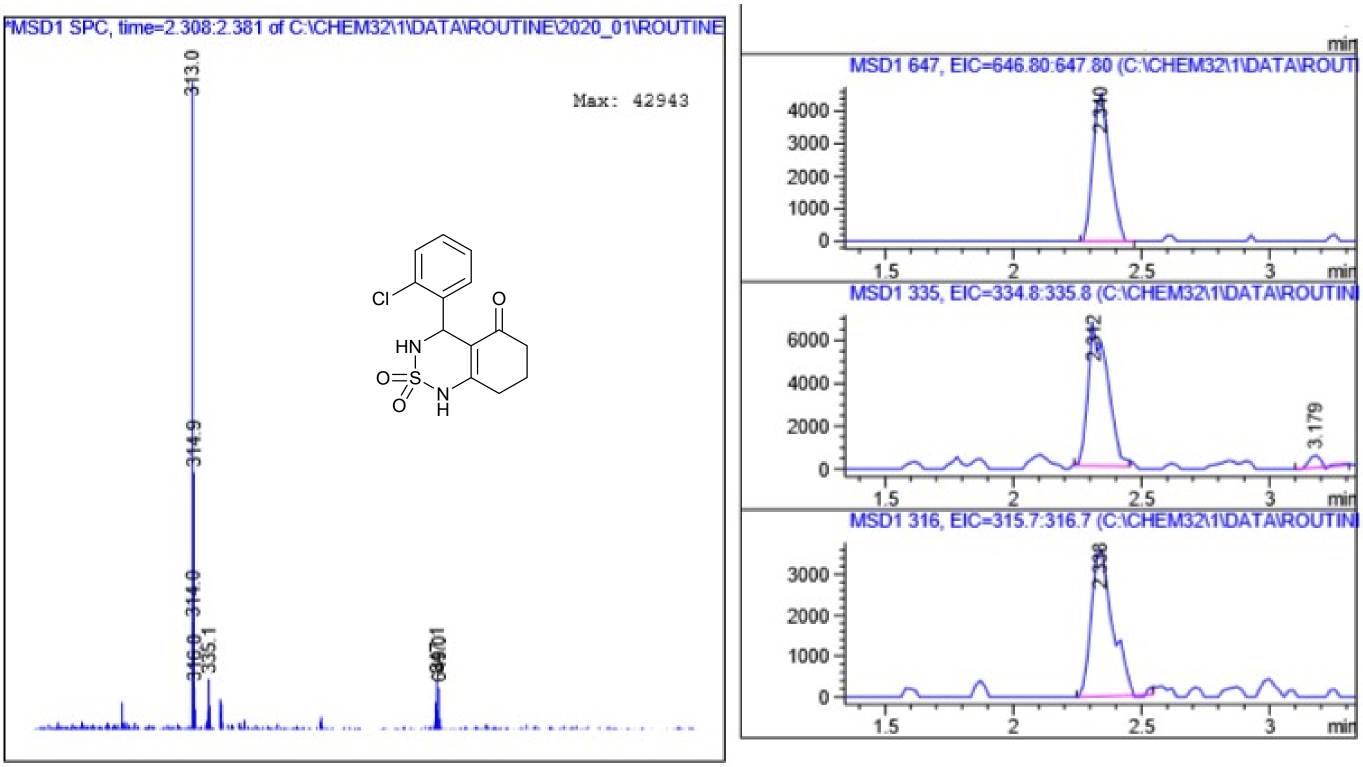

IR Spectrum: 4-(2-chlorophenyl)-4,6,7,8-tetrahydro-1H-benzo[c][1,2,6]thiadiazin-5(3H)-one 2,2-dioxide

LC-MS: 4-(2-chlorophenyl)-4,6,7,8-tetrahydro-1H-benzo[c][1,2,6]thiadiazin-5(3H)-one 2,2-dioxide

Binding disposition of compounds after docking calculations in the active site of Eg5

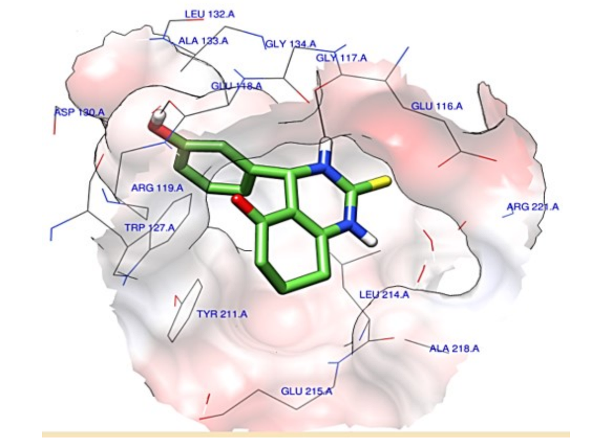

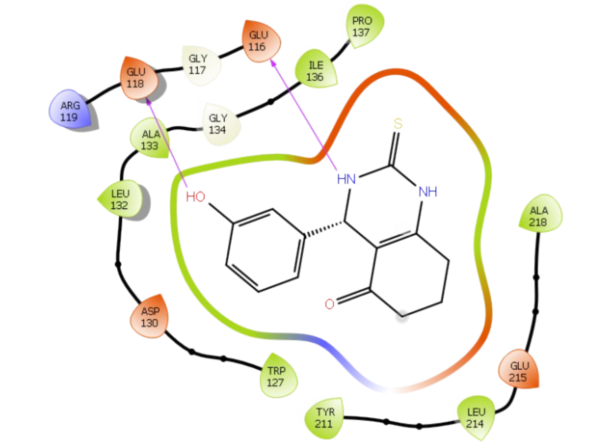

Reference ligand: **Enastron**

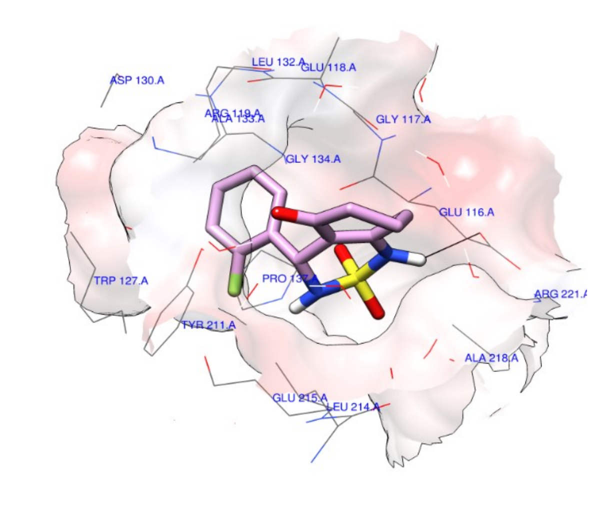

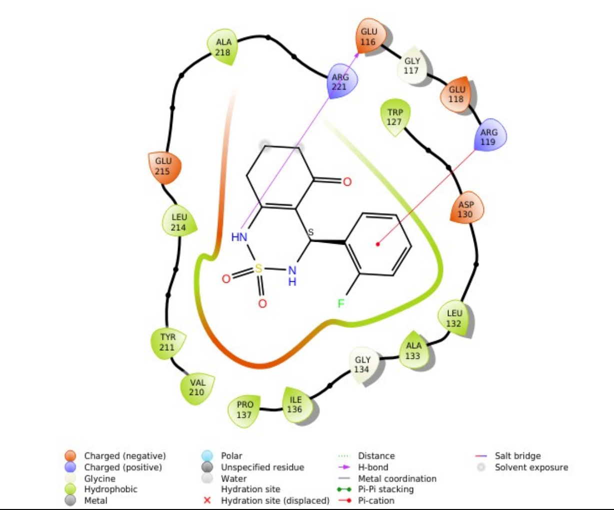

4-(2-fluorophenyl)-4,6,7,8-tetrahydro-1H-benzo[c][1,2,6]thiadiazin-5(3H)-one 2,2-dioxide (**2a**)

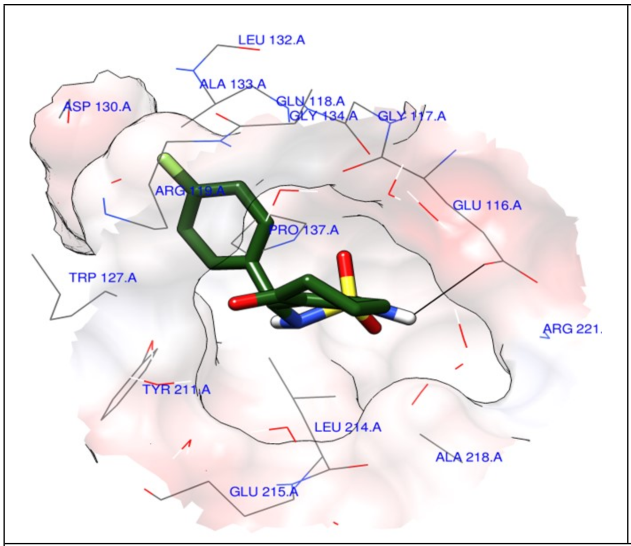

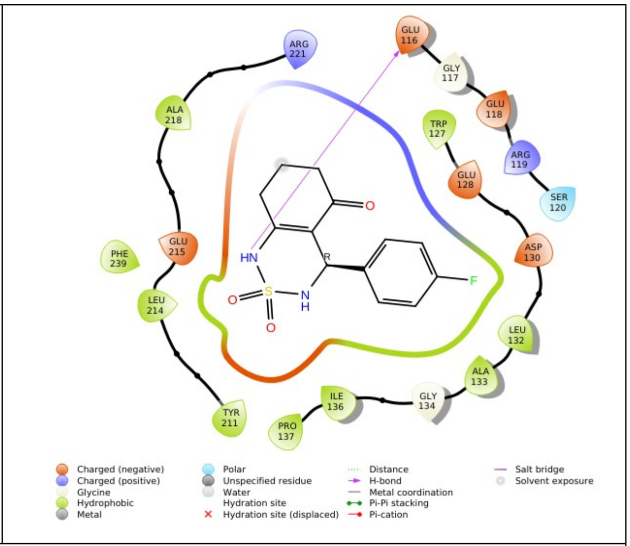

4-(4-fluorophenyl)-4,6,7,8-tetrahydro-1H-benzo[c][1,2,6]thiadiazin-5(3H)-one 2,2-dioxide (**3a**)

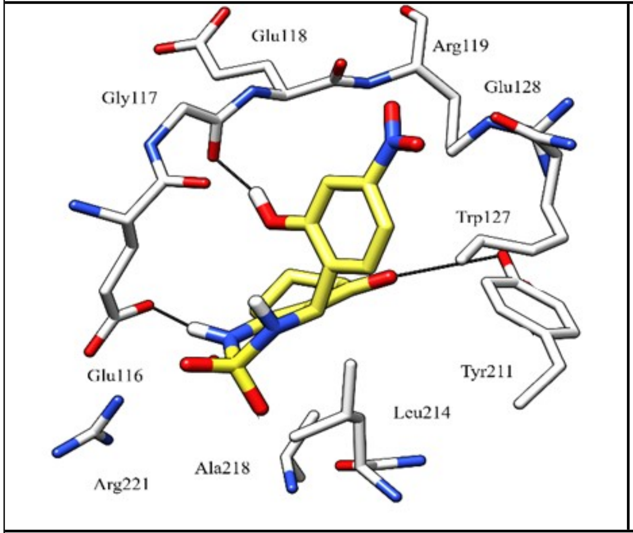

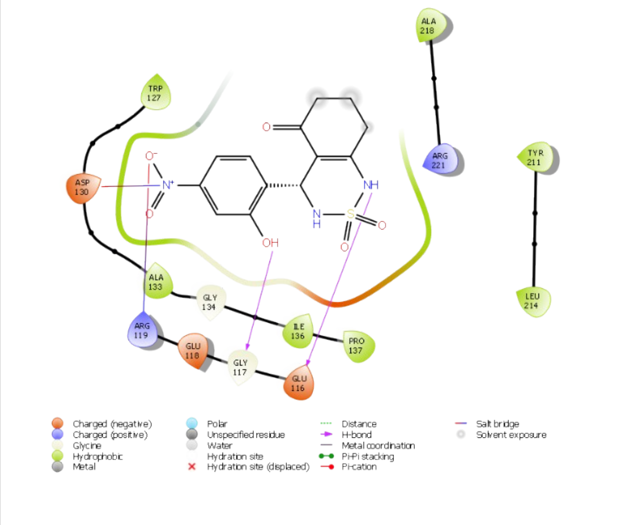

4-(2-hydroxy-4-nitrophenyl)-4,6,7,8-tetrahydro-1H-benzo[c][1,2,6]thiadiazin-5(3H)-one 2,2-dioxide (**6a**)

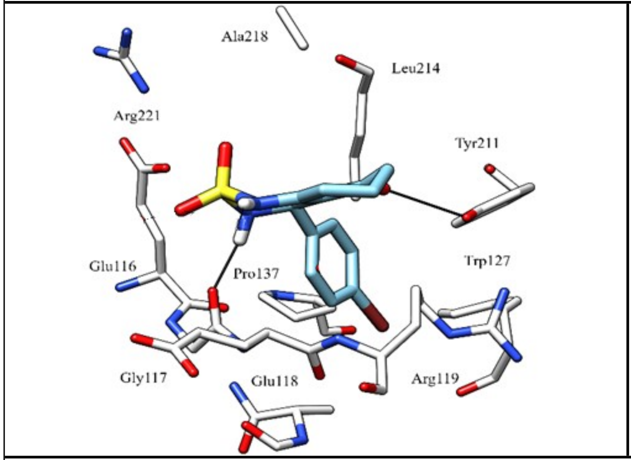

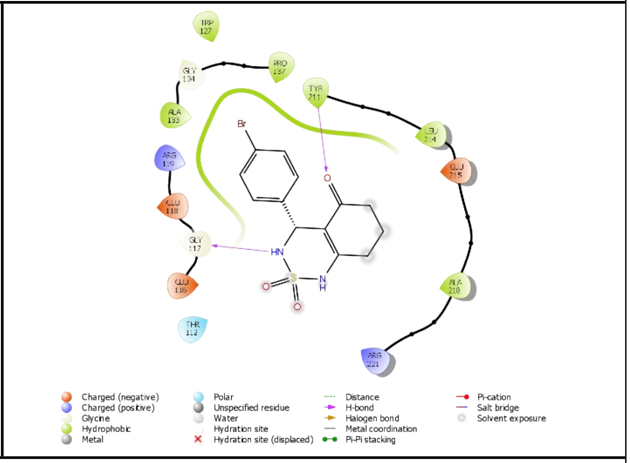

4-(4-bromophenyl)-4,6,7,8-tetrahydro-1H-benzo[c][1,2,6]thiadiazin-5(3H)-one 2,2-dioxide (7**a**)

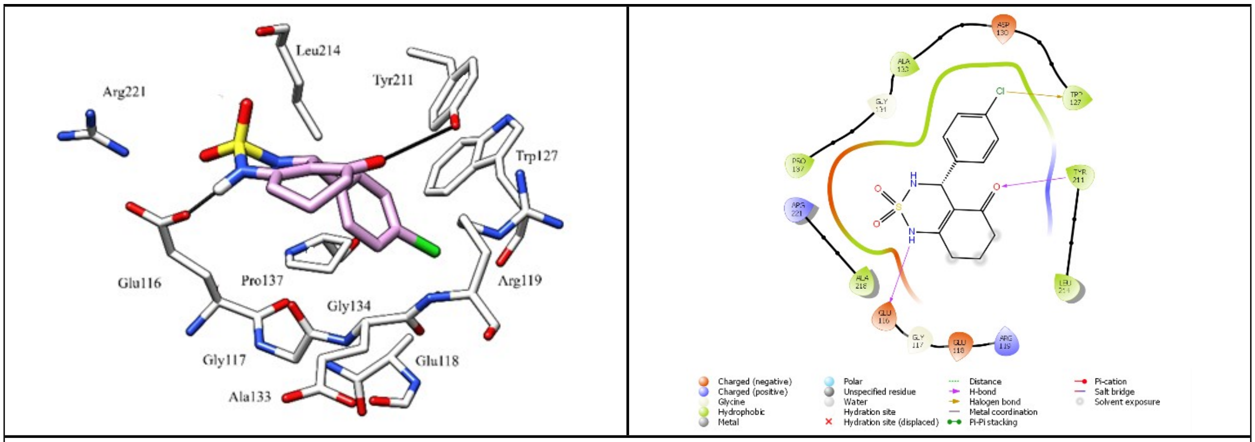

4-(4-chlorophenyl)-4,6,7,8-tetrahydro-1H-benzo[c][1,2,6]thiadiazin-5(3H)-one 2,2-dioxide (8**a**)

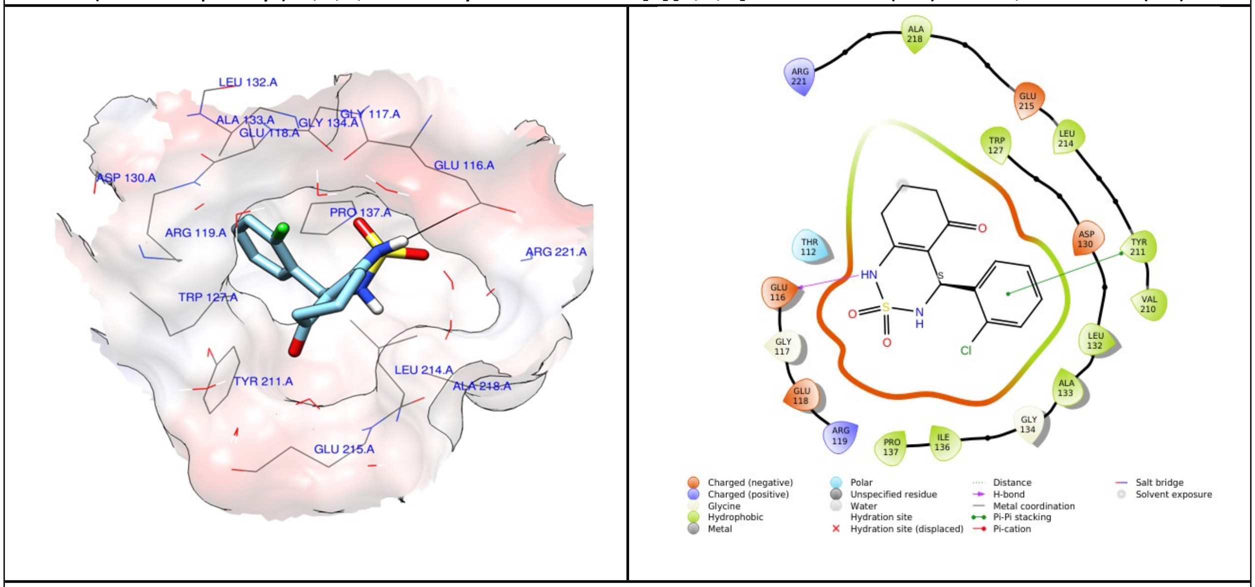

4-(2-chlorophenyl)-4,6,7,8-tetrahydro-1H-benzo[c][1,2,6]thiadiazin-5(3H)-one 2,2-dioxide (**9a**)

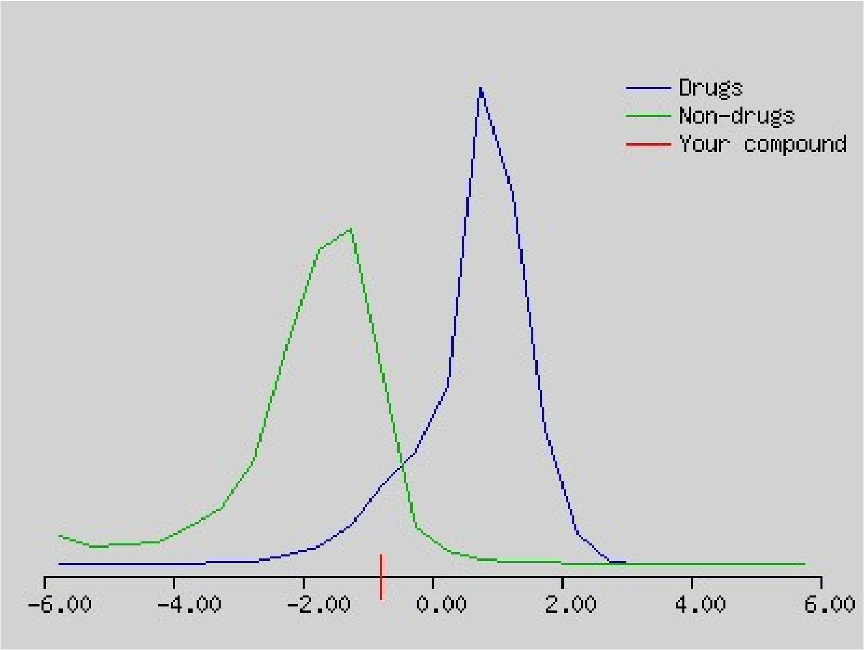

DLS of **1a**

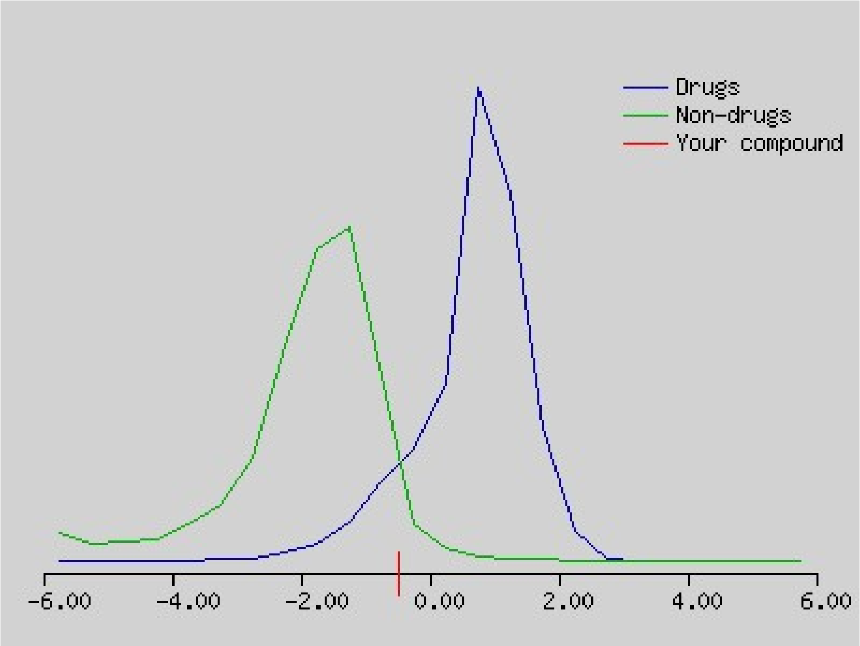

DLS of **2a**

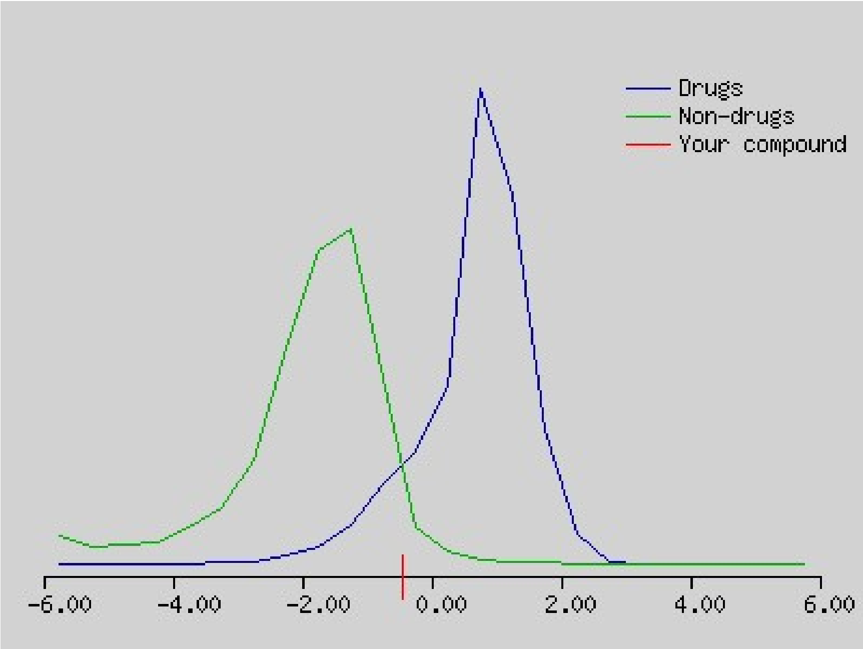

DLS of **3a**

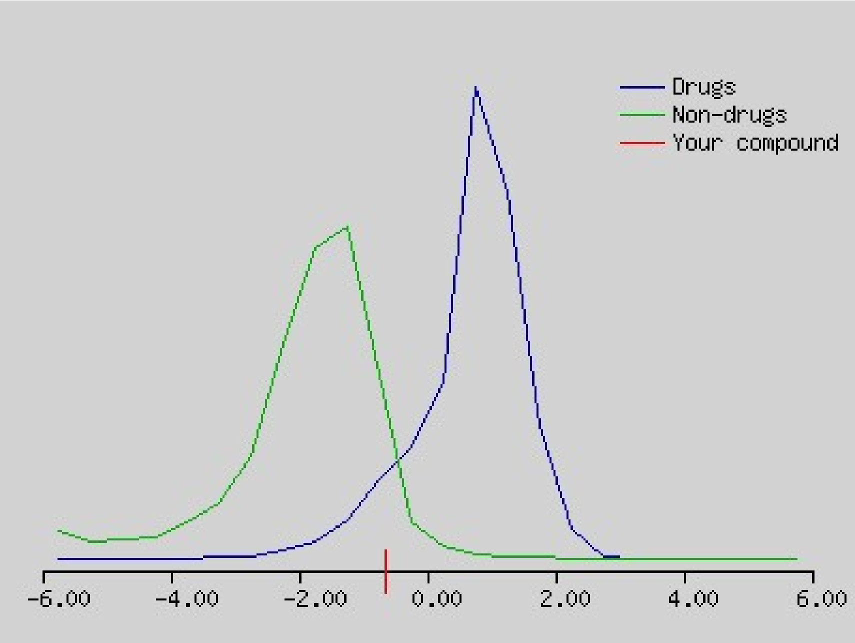

DLS of **4a**

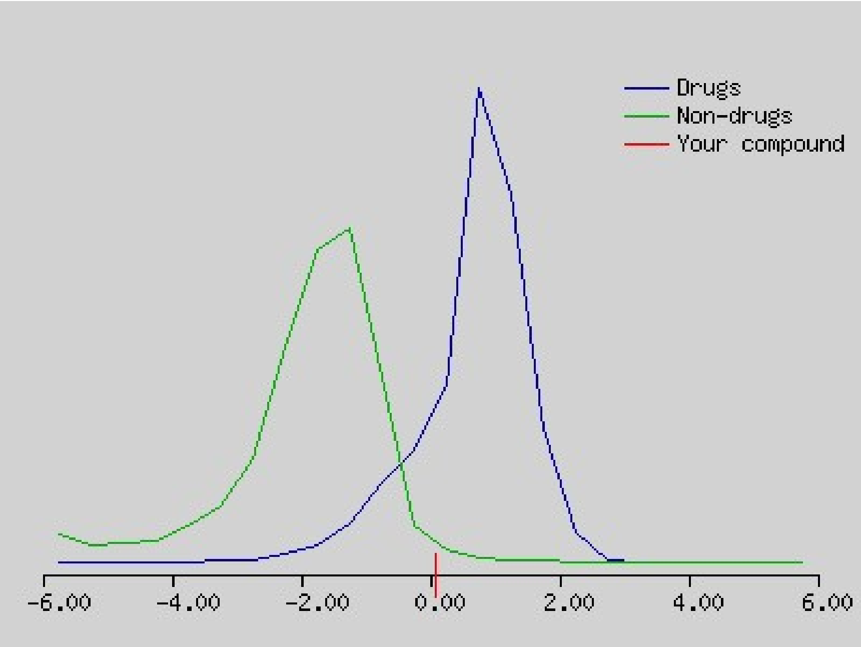

DLS of 6a

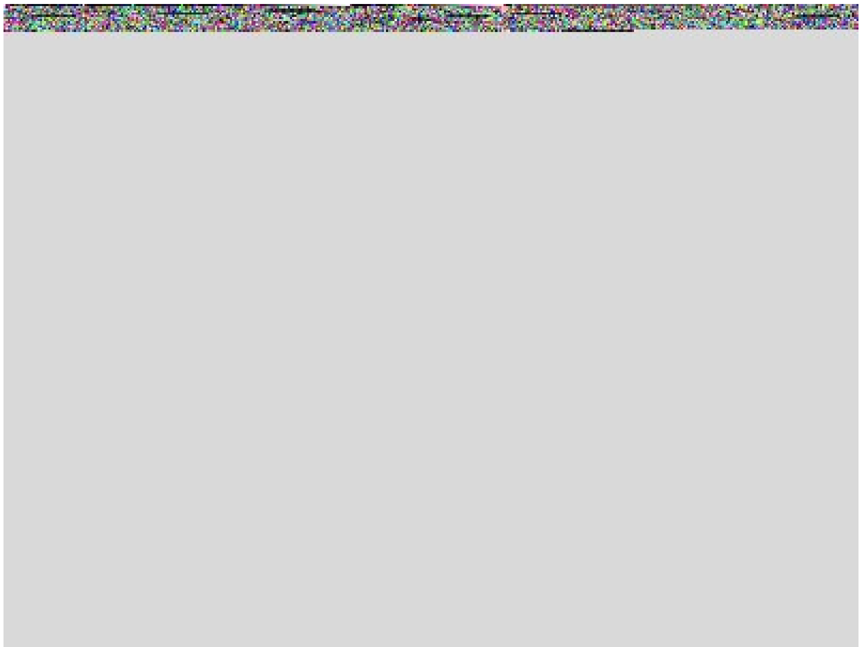

DLS of **7a**

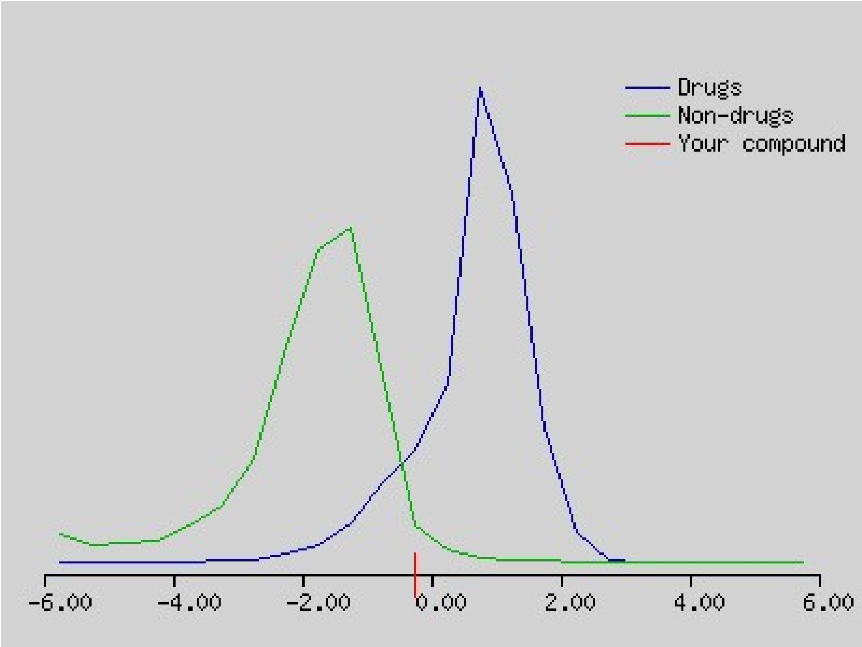

DLS of **8a**

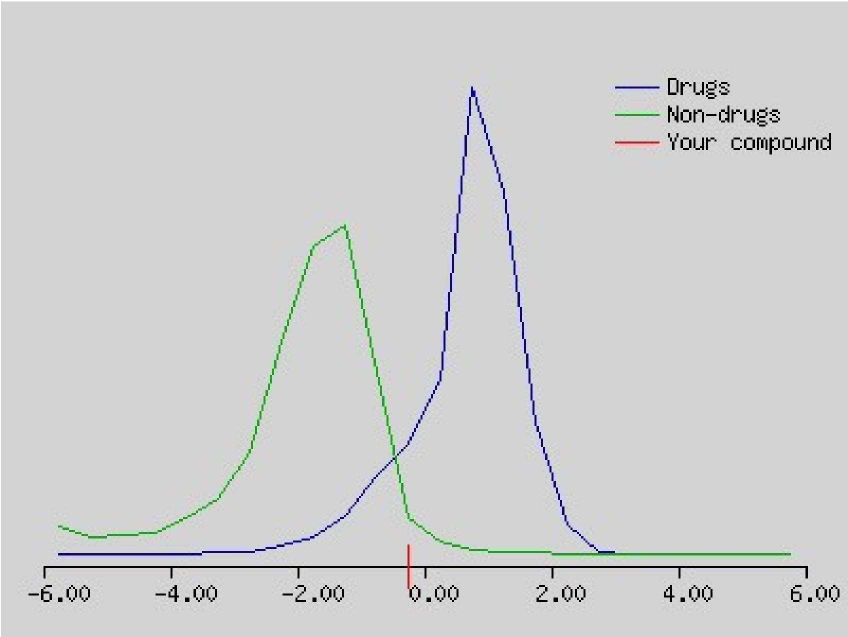

DLS of **9a**
